# Utilization of dietary mixed-linkage β-glucans by the Firmicute *Blautia producta*

**DOI:** 10.1016/j.jbc.2023.104806

**Published:** 2023-05-11

**Authors:** Ravindra Pal Singh, Jayashree Niharika, Raksha Thakur, Ben A. Wagstaff, Gulshan Kumar, Rikuya Kurata, Dhaval Patel, Colin W. Levy, Takatsugu Miyazaki, Robert A. Field

**Affiliations:** 1 Department of Industrial Biotechnology, Gujarat Biotechnology University, Near Gujarat International Finance Tec (GIFT)-City, Gandhinagar, Gujarat, India; 2 Division of Food and Nutritional Biotechnology, National Agri-Food Biotechnology Institute, SAS Nagar, Punjab, India; 3 Department of Chemistry and Manchester Institute of Biotechnology, The University of Manchester, Manchester, UK; 4 Department of Agriculture, Graduate School of Integrated Science and Technology, Shizuoka University, Shizuoka City, Shizuoka, Japan; 5 Research Institute of Green Science and Technology, Shizuoka University, Shizuoka City, Shizuoka, Japan

**Keywords:** mixed-linkage β-glucan (MLG), metabolism, *Blautia producta*, glucan hydrolase, glucan phosphorylase

## Abstract

The β-glucans are structurally varied, naturally occurring components of the cell walls, and storage materials of a variety of plant and microbial species. In the human diet, mixed-linkage glucans [MLG - β-(1,3/4)-glucans] influence the gut microbiome and the host immune system. Although consumed daily, the molecular mechanism by which human gut Gram-positive bacteria utilize MLG largely remains unknown. In this study, we used *Blautia producta* ATCC 27340 as a model organism to develop an understanding of MLG utilization. *B. producta* encodes a gene locus comprising a multi-modular cell-anchored endo-glucanase (*Bp*GH16_MLG_), an ABC transporter, and a glycoside phosphorylase (*Bp*GH94_MLG_) for utilizing MLG, as evidenced by the upregulation of expression of the enzyme- and solute binding protein (SBP)–encoding genes in this cluster when the organism is grown on MLG. We determined that recombinant *Bp*GH16_MLG_ cleaved various types of β-glucan, generating oligosaccharides suitable for cellular uptake by *B. producta*. Cytoplasmic digestion of these oligosaccharides is then performed by recombinant *Bp*GH94_MLG_ and β-glucosidases (*Bp*GH3-AR8_MLG_ and *Bp*GH3-X62_MLG_). Using targeted deletion, we demonstrated *Bp*SBP_MLG_ is essential for *B. producta* growth on barley β-glucan. Furthermore, we revealed that beneficial bacteria, such as *Roseburia faecis* JCM 17581^T^, *Bifidobacterium pseudocatenulatum* JCM 1200T, *Bifidobacterium adolescentis* JCM 1275T, and *Bifidobacterium bifidum* JCM 1254, can also utilize oligosaccharides resulting from the action of *Bp*GH16_MLG_. Disentangling the β-glucan utilizing the capability of *B. producta* provides a rational basis on which to consider the probiotic potential of this class of organism.

The β-glucans are components of the cell walls of marine macroalgae (1), yeasts (2), staple crops (3), and bacterial exopolysaccharides (4). These complex and heterogeneous materials possess a range of backbone linkages [as in β-(1,2)-, β-(1,3)-, β-(1,3/4)- and β-(1,6)- glucans], side chain composition, and degree of polymerizations (DP) (5). Cereal β-(1,3/4)-glucans (mixed linkage glucans [MLG]) are a significant component of the cell walls of barley, oats, and rye, and are present in minor quantities in wheat (6). Cereals have been staple food for humans for millennia and are also in widespread use for livestock feed. The consumption of grain rich in soluble MLG correlates with benefits for the cardiovascular system, giving rise to reduced risk of coronary heart disease (7). While efforts to determine the metabolism of MLG in Gram-negative bacteria (*e.g.*, *Bacteroides ovatus*) have been reported (8), the corresponding processes in Gram-positive bacteria remain to be established, as do the full details of routes to beneficial health impact.

The human genome encodes a modest repertoire of Carbohydrate-Active enZymes (CAZymes) (9), including those associated with the cleavage of α-linked glucans, such as starch (10). However, the MLGs are not recognized by human genome-encoded digestive enzymes, resulting in their passage through the digestive system to the lower gastrointestinal tract (GIT). These dietary MLGs become food for the human gut microbiota (HGM), influencing the composition of the microbial community and the metabolites that they produce (11). The HGM predominantly consists of bacteria belonging to *Bacteroidetes, Actinobacteria, Proteobacteria, and Firmicutes* (12). It is estimated that bacterial communities of these phyla collectively harbor ∼150-fold more genes than the host genome and that they have an underlying impact on host physiology (13). These bacterial genes comprise a variety of CAZymes and transporters, which play a crucial role in facilitating host metabolism through the digestion of complex glycans (14, 15). Among the downstream metabolites arising from dietary polysaccharide degradation, butyrate primarily provides energy to colonocytes (16), supporting an anti-carcinogenic, anti-inflammatory environment, and strengthening gut barrier functions (16, 17). In contrast, acetate primarily nourishes gut-beneficial bacteria (18), helping to maintain gut microbial homeostasis. It also crosses the blood–brain barrier and is a source of energy for the brain (19). Short- or long-term therapeutic intervention, for instance with antibiotics, can impact the gut microbiome, leading to dysbiosis and altering functional and metabolic activities (20). Improving understanding in this space, accompanied by a better molecular understanding of the mechanisms by which individual members of the microbiota utilize complex glycan, is expected to contribute substantially to the rational development of nutritional or therapeutic interventions that restore gut homeostasis and normal gut function.

The utilization of complex glycans by members of Gram-negative *Bacteroidetes* is well known, with associated organisms possessing gene clusters, so-called Polysaccharide Utilization Loci (PULs) (21, 22). A representative structure for barley MLG is shown in Figure 1*A*; other glucans with a β-1,3-linked backbone include curdlan (unbranched) (4), laminarin (β-1,6 linked single glucose as side chains) (1, 23), and yeast β-glucan (long β-1,6-linked glucan side chains)- such β-1,6-linked glucan side chains absent in cereals (8, 24, 25). An MLG-utilizing molecular mechanism has been identified in the Gram-negative bacterium *B. ovatus* ATCC 8483 (8), where a gene locus centered on tandem susC/susD homologs that code for the TonB-dependent transporter (TBDT or SusC), extracellular anchored endo-acting enzyme (*Bo*GH16_MLG_), periplasmic exo-acting enzyme (*Bo*GH3_MLG_) and the cell-surface glycan-binding protein (*Bo*SGBP-A and *Bo*SGBP-B) (Fig. 1*B*). *Bo*SGBP-B attaches to MLGs in the gut lumen and *Bo*GH16_MLG_ cleaves them into oligosaccharides, which are trapped by the SusD homolog (*Bo*SGBP-A) of a transporter and are brought into periplasmic space by a SusC transporter. Once they are inside the cell, *Bo*GH3_MLG_ cleaves these oligosaccharides into glucose, which is subsequently transported into the cytoplasm for further metabolism. In contrast, although Gram-positive *Firmicutes* phylum is dominant in the HGM, little is known about how members of this phylum utilize MLG. *Firmicutes* typically lack the repertoire and organization of genes present in *Bacteroides* PULs (26) and display different approaches for utilizing dietary glycans, as identified in *Roseburia* for mannan (27). In particular, Sheridan *et al.* (26) identified that Gram-positive PUL (gpPULs) of *Roseburia* and *Eubacterium rectale* typically consist of glycoside hydrolases, ABC transport proteins, and transcriptional regulator genes.

**Figure 1:**
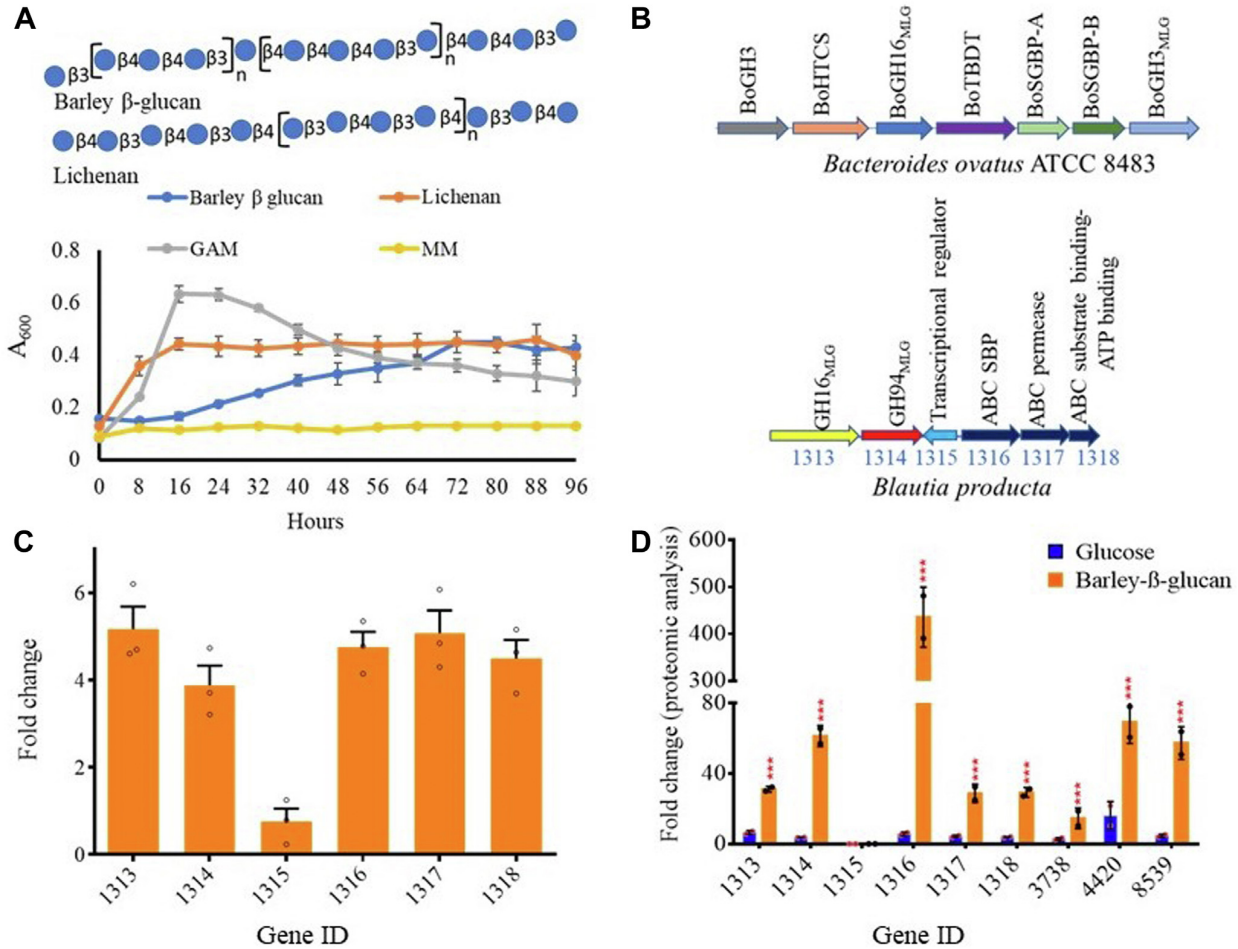
**Growth and regulation of a β-glucans utilization locus in *Blautia producta.****A*, representative structures of barley β-glucan and lichenan as per symbolic nomenclature for glycans (https://www.ncbi.nlm.nih.gov/glycans/snfg.html). Growth of *B. producta* was performed on a minimal medium (MM) containing 1% barley β-glucan or lichenan. *B*, β-glucan utilization locus in *B. producta* and the corresponding locus of *Bacteroides ovatus* ATCC 8483 (8). *C*, gene expression analysis of β-glucan utilization locus through RT-qPCR after growing cells of the *B. producta* on a MM containing 1% barley β-glucan. Changes in the expression level of these genes present in the locus were calculated as fold change and RT-qPCR data were normalized to 16S rRNA transcript levels. *D*, in addition to genes present in the locus, other cytoplasmic genes involved in the utilization of barley β-glucan were identified through proteomics. Proteomic data represent two replicates, and differences in expression levels between glucose and barley β-glucan grown cultures were analyzed by two-way ANOVA (∗∗∗*p* = 0.001). GAM, Gifu anaerobic medium. UniProt IDs for *bp3738, bp4420*, and *bp8539* are A0A6P1Z_5U5_, A0A6P1Z_AR8_, and A0A6P1Y_X62_ respectively.

Firmicute *Blautia producta* ATCC 27340 is an anaerobic cocci-shaped bacterium of the Lachnospiraceae family. It is commonly present in the human GIT, and it has recently been reported as a potential probiotic (28) by virtue of its ability to eliminate inflammatory and metabolic diseases (28) and restore colonization resistance against vancomycin-resistant *Enterococcus* (29, 30). While established as an important species in the GIT, the mechanistic basis of its nutritional impact remains elusive. Our previous study (31) showed that *B. producta* can grow on β-(1,3)-linked short-length glucans, but the mechanism by which it processes such glucans to glucose for further metabolism remains unknown. Here we use an interdisciplinary approach to unravel the molecular mechanism employed by *B. producta* to depolymerize MLG. Our findings show that the growth of *B. producta* on MLG is highly dependent on the expression of a gene locus encoding a multi-modular cell-anchored endo-glucanase, an ABC transporter, intracellular β-glucosidase, and intracellular phosphorylases. These proteins work in concert to effect complete degradation of MLG to glucose or/and glucose-1-phosphate. Through whole-cell experiments, we demonstrate that *B. producta* endo-glucanase can facilitate cross-feeding on MLG-derived β-glucans to various beneficial bacterial species. Overall, this study confirms the potential for developing targeted intervention strategies to improve human health by manipulating the gut microbiota with dietary mixed-linkage β-glucans.

## Results

### Growth of *B. producta* and identification of a β-glucan utilization locus

*B. producta* ATCC 27340 was able to grow on barley and lichenan MLG as well as Gifu anaerobic medium (GAM - used as a control) (Fig. 1*A*), and genes that may allow this to happen were identified using the JGI/IMG online server (32). An open reading frame encoding the β-(1,3/4)-glucanase of *B. ovatus* ATCC 8483 (*Bo*GH16_MLG_) (8) was chosen as a query sequence for BlastP analysis of the genome of *B. producta* (33). This led to the identification of a putative *B. producta* β-(1,3/4)-glucanase, with a predicted 33% amino acid similarity in its catalytic domain to the *B. ovatus* sequence. Distinct from its *B. ovatus* (∼30 kDa) counterpart, *Bp*GH16_MLG_ contains four CBM4 family carbohydrate-binding modules (http://www.cazy.org/), leading to a larger molecular weight of ∼130 kDa. Genomic analysis with this lead *B. producta* sequence identified a locus comprising genes for a putative endo β-glucanase (*bp1313*- *Bp*GH16_MLG_), cellobiose phosphorylase (*bp1314*), an AraC-type DNA-binding protein (*bp1315*), and genes for an ATP binding cassette (ABC) transporter (substrate-binding protein- SBP, permease protein and ABC subunits couple the binding/hydrolysis of ATP, *bp1316*–*bp1318*) (Fig. 1*B*).

To confirm the involvement of this gene locus in the utilization of MLG, *B. producta* was cultured on barley β-glucan, and RT-qPCR was employed to evaluate the transcriptional level of the locus-associated genes. The genes encoding *bp1313* and *bp1314* were upregulated by 5.2 and 3.9-fold, respectively, in the presence of barley β-glucan compared to growth on glucose. Phylogenetic analysis of Bp1313 and Bp1314 established them as a β-glucanase (GH16 family) and a glucan phosphorylase (GH94 family), hereafter referred to as *Bp*GH16_MLG_ and *Bp*GH94_MLG_ (Figs. S1 and S2). Expression of *bp1316, bp1317*, and *bp1318* was upregulated by 4.7-, 5-, and 4.5-fold for growth on barley MLG *versus* glucose, respectively (Fig. 1*C*). A 0.76-fold upregulation of transcriptional regulator *bp*1315 was observed for growth on barley β-glucan compared to glucose; a similarly modest change in expression of a transcriptional regulator was previously observed for the utilization of mannan (27). We next compared the proteomes of *B. producta* grown on barley β-glucan *versus* glucose: among CAZymes, five proteins were abundant (Fig. 1*D*). Bp1313 and Bp1314 were expressed in abundance by approximately 5- and 16-fold on barley β-glucan compared to glucose. Proteomic analyses did not detect membrane-bound Bp1315 under either set of growth conditions. The production of *Bp*SBP (Bp1316) increased by 77-fold, whereas Bp1317 and Bp1318 were expressed by about 7-fold each. Among these proteins, one protein (Bp3738) belongs to CAZy family GH18, and two proteins (Bp4420 and Bp8539) belong to GH3; they were up-regulated by 5.1, 3.4, and 12.4-fold, respectively. An NCBI Conserved Domain database search highlighted that Bp3738 may not act on β-glucan (34). Furthermore, this analysis suggested that Bp3738 may cleave chitin, as similar domain-containing enzymes have previously been characterized (http://www.cazy.org/GH18.html). Thus, further analysis was not performed on this protein.

### Cellular localization and enzymatic activity of *Bp*GH16_MLG_

#### Cellular localization

**Figure 2:**
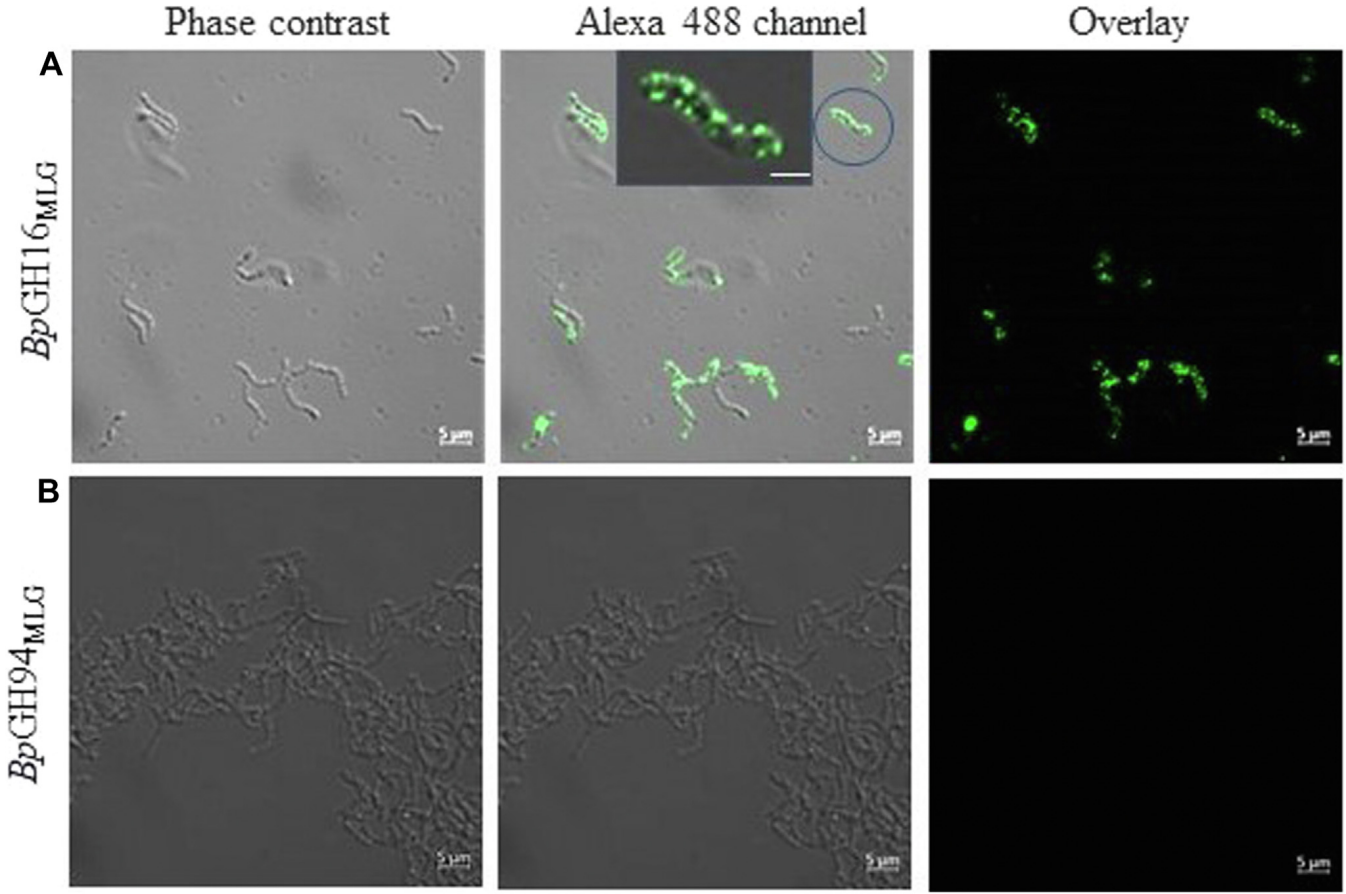
**Enzyme localization analysis.***A* and *B*, phase-contrast confocal microscopy, corresponding fluorescence microscopy, and merged images of *B. producta* cells grown on a minimal medium containing barley β-glucan as the sole carbon source. Cells were probed with custom polyclonal antibodies against rec*Bp*GH16_MLG_ (*A*) and rec*Bp*GH94_MLG_ (*B*). Insert shows zoomed-in view of the bacterial surface revealing that *Bp*GH16_MLG_ is present at the outer membrane of a cell. Scale bar for zoom image and all other images are 0.5 μm and 5 μm, respectively.

*Bp*GH16_MLG_ is implicated in the cell surface cleavage of MLG in *B. producta*, an essential first step for barley β-glucan utilization. Accordingly, LipoP 1.0 predicted a type I signal sequence (score = 14.00) in its N-terminal region (35). To confirm the prediction of cell surface association, *B. producta* cells grown in a minimal medium containing 1% barley β-glucan were subjected to immunolocalization studies with custom polyclonal antisera raised against rec*Bp*GH16_MLG_. The *Bp*GH16_MLG_ was clearly visualized on the outer surface of bacterial cells (Fig. 2*A*). Similar experiments using custom antisera raised against rec*Bp*GH94_MLG_ gave no fluorescent signal (Fig. 2*B*), consistent with the expected intracellular expression of *Bp*GH94_MLG_. Indeed, no signal peptide was predicted for this protein when analyzed with SignalP, v. 4.0 (36). Despite this, it has the potential for binding with *Bp*GH94_MLG_ as shown in Fig. S3.

#### Enzymatic activity of *Bp*GH16_MLG_

The optimum temperature and pH for recombinant *Bp*GH16_MLG_ were determined to be 37 °C and pH 5.7 (phosphate buffer), respectively (Fig. S4). In the initial screening, *Bp*GH16_MLG_ did not show activity with maltose [α-(1,4) -linked glucose], gentiobiose [β-(1,6) -linked glucose], cellobiose [β-(1,4)-linked glucose], cellotriose [β-(1,4) -linked glucose], laminaribiose [β-(1,3)-linked glucose], and sucrose [α-(1,2)-linked glucose and fructose] (Fig. S5*A*). It did cleave oat β-glucan, yeast β-glucan, lichenan, lentinan, laminaritriose, laminarin, and barley-β-glucan (Fig. S5, *B*–*E*), suggesting that it has a cleavage specificity for β-(1,3)-linked glucans and a minimum trisaccharide is required for its activity. Subsequent Michaelis-Menten kinetic analysis revealed a higher specificity constant (k_cat_/K_m_) for barley β-glucan and lichenan over laminarin and oat β-glucan, respectively (Table 1 and Fig. S6). Kinetic parameters and potent activity of *Bp*GH16_MLG_ on barley β-glucan and lichenan as compared to laminarin (Table 1 and Fig. S7, *A*–*E*) suggest that this enzyme is β-(1,3/4) glucanase with an ability to cleave β-(1,3)-linked glucans (37). Although *Bp*GH16_MLG_ is highly active on laminarin, it does not support the growth of *B. producta* on this (31), which may indicate that the structural features of laminarin are unable to activate the gpPUL.

**Table 1:** Kinetic parameters of native and mutant *B*_*P*_GH16 on various β glucans

Enzyme	Substrate	K_m_ (mg ml^−1^)	k_cat_ (s^−1^)	k_cat_/K_m_ (s^−1^ mg^−1^ ml)
*B*_*P*_GH16	Laminarin	0.24 ± 0.03	43 ± 0.6	179
	Lichenan	0.13 ± 0.02	36 ± 0.5	275
	Barley β-glucan	0.22 ± 0.04	62 ± 1.2	281
	Oat β-glucan	0.32 ± 0.05	46 ± 0.8	148

Mutant	Substrate	K_m_ (mg ml^−1^)	k_cat_ (s^−1^)	k_cat_/K_m_ (s^−1^ mg^−1^ ml)
R331A	Barley-β-glucan	0.35 ± 0.05	48 ± 0.8	137
L358A		0	0	0
E379A		0	0	0
D381A		0.17 ± 0.04	35.7 ± 1.1	210
E384A		0	0	0
W362A		0	0	0
H398A		0	0	0

Maltose was used to generate a standard curve. Kinetic parameters of mutant enzymes were determined with barley β-glucan in order to demonstrate the role of each residue in overall activity. Kinetic parameters were performed using DNS assay McKee (84).

**Figure 3:**
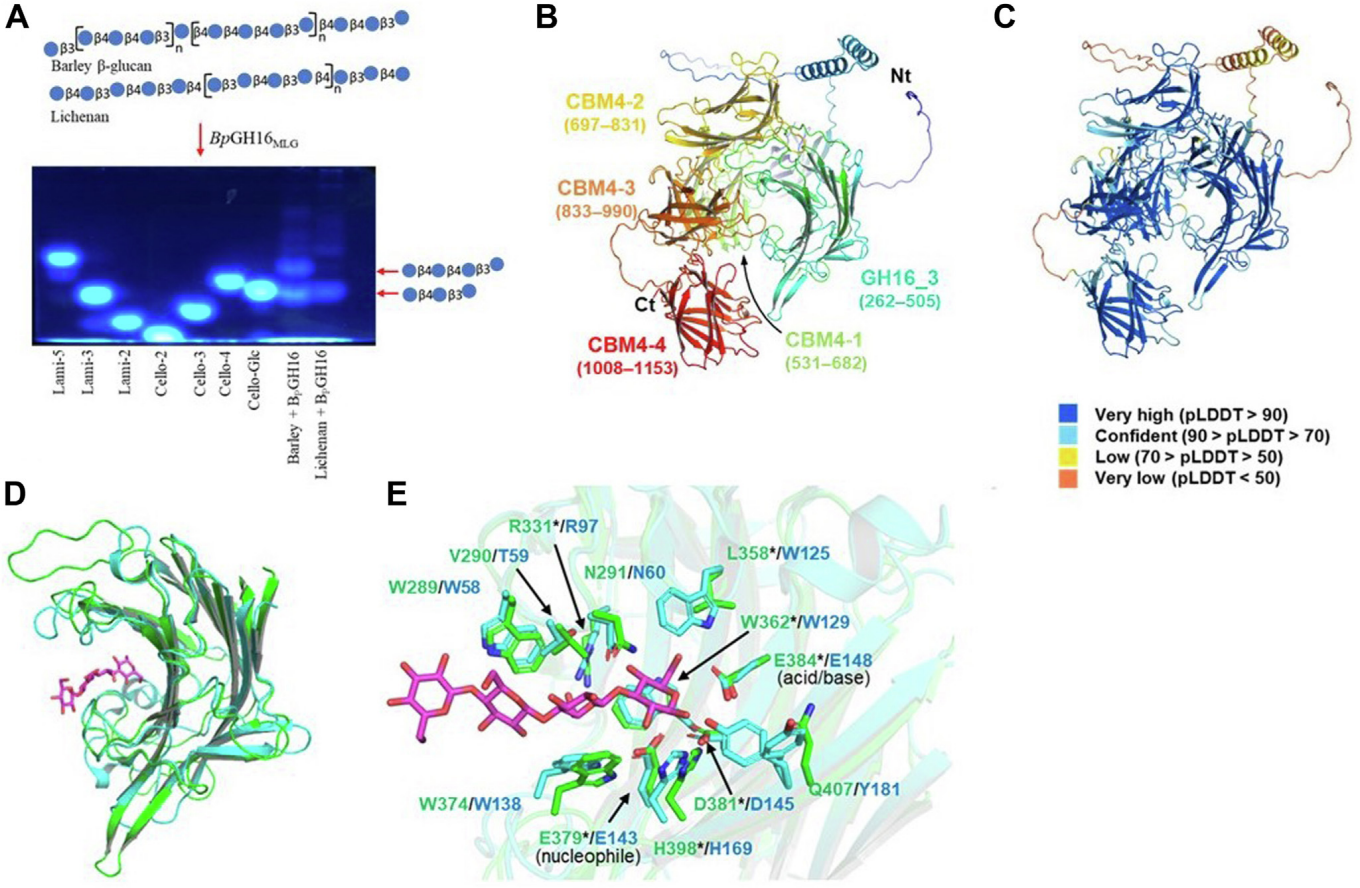
**AlphaFold2 structure prediction and identification of critical residues of *Bp*GH16**_**MLG**_**from *B. producta* ATCC 27340.***A*, oligosaccharides obtained from the *Bp*GH16_MLG_ hydrolyzed barley-β-glucan and lichenan were fluorescently tagged and run onto a 37% polyacrylamide gel electrophoresis. *B*, the AlphaFold2 model of full-length *Bp*GH16_MLG_. The ribbon model is colored in a rainbow from N terminus (Nt, *blue*) to C terminus (Ct, *red*). The GH16 subfamily three catalytic domains (GH16_3) and four CBM4 domains (CBM4-1 to CBM4-4) are indicated with residue numbers in parenthesis. *C*, the ribbon model is colored based on pLDDT score. *D*, superimposition of *Bp*GH16_MLG_ (*green*) on *Bo*GH16_MLG_ (*cyan*) in complex with G4G4G3G (PDB 5NBP, *magenta stick*). *E*, comparison of active sites of *Bp*GH16_MLG_ (*green*) and *Bo*GH16_MLG_ (*cyan*). The residues mutated to alanine in this study are labeled with asterisk. G4G4G3G is shown in *magenta*.

Generation of a limit digest for barley β-glucan with *Bp*GH16_MLG_ yielded tri- and tetra-saccharide products (Fig. 3*A*), as evidenced by TLC and MALDI-TOF-MS analyses (Fig. S7, *C* and *D*). Using a combination of fluorophore-assisted carbohydrate electrophoresis (FACE) (Fig. S8*A*), MALDI-TOF-MS (Fig. S8, *B* and *C*) and NMR analysis (Fig. S8*D*), these glucans were confirmed to be Glc-β-(1,4)-Glc-β-(1,4)-Glc-β-(1,3)-Glc)- [hereafter G4G4G3G] and Glc-β-(1,4)-Glc-β-(1,3)-Glc)- [hereafter G4G3G]. The same glucans were previously identified as the limit cleavage from the action of *B. ovatus Bo*GH16_MLG_ (8), which is to be expected based on the sequence similarity of the catalytic domains of the two enzymes. Further analysis suggested that the catalytic domain of *Bp*GH16_MLG_ belongs to subfamily GH16_3 (38), along with *Bo*GH16 (Fig. S1).

Structure modeling of *Bp*GH16_MLG_ was performed using AlphaFold2 (Fig. 3*B*) and predicted local distance difference test (pLDDT- Figs. 3*C* and S9) (39, 40). The model structure of *Bp*GH16_MLG_ (green) was superimposed on *Bo*GH16_MLG_ (cyan) in complex with G4G4G3G (PDB 5NBP, magenta stick) in order to identify essential catalytic residues and to provide insight into *Bp*GH16_MLG_ substrate binding (Fig. 3*D*). A signature sequence, EXDXXE, is generally present in the active site of mixed-linkage endo-β-glucanases belonging to GH16 (41). This signature sequence is also present in the active site of the *Bp*GH16_MLG_, where the first and last 'E' and D represent E379, E384, and D381, respectively (Fig. 3*E*). Alanine scanning mutagenesis on these and other residues present at the putative active site was performed, based on structural alignment (Fig. 3*E*). As expected, mutation of the catalytic nucleophile (E379A) or the catalytic base (E384A) of the EXDXXE motif gave rise to inactive enzymes. In contrast, mutation of the electrostatic helper (D381A) resulted in a 1.7-fold reduction in k_cat_, accounting for an overall 1.34-fold loss in activity on barley β-glucan (Table 1). For other active site residues (Fig. 3*E*): R331A had a modest impact on K_m_ (<2-fold increase) and k_cat_ (∼1.3-fold reduction); L358A, W362A, and H398A led to inactivation of the enzyme.

As noted above, *Bp*GH16_MLG_ contains four CBM4 family carbohydrate-binding modules following the GH16 catalytic domain (residues 262–505): CBM4_1 (531–682), CBM4_2 (697–831), CBM4_3 (833–990), and CBM4_4 (1008–1153) (Fig. 3*B*) as predicted with DeepTMHMM (42). A similar arrangement of four homologous CBMs was previously reported in the lichenase A/laminarinase (LicA) from *Clostridium thermocellus* (*Ct*CDP) (43). The *Bp*GH16_MLG_ CBMs show little to no sequence similarity to each other: while *Bp*CBM4_1 showed 25% sequence similarity to *Bp*CBM4_4, *Bp*CBM4_2, and *Bp*CBM4_3 do not show any sequence similarity with other *B. producta* CBM4s. However, *Bp*CBM4_1, *Bp*CBM4_2, *Bp*CBM4_3, and *Bp*CBM4_4 revealed 33%, 28%, 40%, and 41% sequence similarity with *Ct*CBM4_1, *Ct*CBM4_2, *Ct*CBM4_3 and *Ct*CBM4_4, respectively. Interestingly, the kinetic parameters for *Bp*GH16_MLG_ and *Bo*GH16_MLG_ with barley MLG are rather similar, with K_m_ 0.22 ± 0.04 and 0.364 ± 0.051, and k_cat_ 62 ± 1.23 and 85.8 ± 4.6, respectively, suggesting that the CBMs afford no direct kinetic advantage to the *Blautia* enzyme. Although the CBMs appear not to matter for the hydrolysis of MLG in dilute solution under the assay conditions, they might play an important role in binding to insoluble plant cell wall digestion in the complex environment of the GIT.

### Recognition and uptake of oligosaccharides released by *Bp*GH16_MLG_

Next, we focused on the solute binding protein (SBP) of the ABC transporter which was expected to have a carbohydrate-binding role. Gram-positive bacteria use SBPs to capture oligosaccharides from the extracellular environment for carbon utilization (44, 45, 46). Oligosaccharides captured by SBPs are released into the permease of the ABC transporter, which is formed of two transmembrane domains where the translocation of oligosaccharides is coupled with ATP hydrolysis by the cytoplasmic nucleotide-binding domain (47). Although functions of SBPs for utilization of some oligosaccharides in other members of the Lachnospiraceae are known (*e.g.*, *Roseburia*) (27), utilization of β-glucans has not been studied to date.

The LipoP-1.0 and SignalP, v. 4.0 servers suggested that *Bp*SBP_MLG_ has an N-terminal lipid-anchored signal peptide sequence (score = 17.4). Consequently, *Bp*SBP_MLG_ without signal sequence was cloned into a pET28a(+) and the protein was expressed in *Escherichia coli*, giving high levels of recombinant protein (∼80 mg/L). The thermodynamic parameters for binding of various oligosaccharides by *Bp*SBP_MLG_ were evaluated by ITC (Table 2 and Fig. S10). *Bp*SBP_MLG_ showed no detectable affinity for maltose, gentiobiose or lactose, but displayed some affinity towards cellobiose and laminaribiose, while the corresponding trisaccharides and mixed linkage G4G3G were bound 9 to 30 times more tightly (Table 2 and Fig. S10). Additionally, the *Bp*SBP_MLG_ could also accommodate cellotetraose (K_d_ = 0.75 μM), G4G4G3G (K_d_ = 5.14 μM), and laminaritetraose (K_d_ = 5.6 mM), indicating a preference for β-(1,4)- linked-oligosaccharides. In terms of binding stoichiometry (n), G4G4G3G showed n = 0.77 when compared to 0.45 for cellotetraose, suggesting a preference for linear tetrasaccharide binding (Table 2). The β-mannooligosaccharide binding protein of *Roseburia* displayed similar binding affinity to mannotetraose with K_d_ and n values of 3.89 μM and 0.7, respectively (27).

**Table 2:** Thermodynamics of binding of oligosaccharides to SBP_MLG_ measured by ITC at 25 °C in 10 mM in sodium phosphate (pH 7.0)

Ligand	Represntative images	K_d_ (M)	ΔG^0^ (kcal mol^−1^)	ΔH (kcal mol^−1^)	-TΔS^0^ (kcal mol^−1^)	n
G4G3G		(1.01 ± 0.1) × 10^−6^	−8.18	−12.6 ± 0.32	4.4	0.42 ± 0.01
G4G4G3G		(5.14 ± 0.01) × 10^−6^	−7.22	−12.9 ± 0.85	5.7	0.77 ± 0.02
Cellobiose		(30.6 ± 7.09) × 10^−6^	−6.2	−7.0 ± 2.2	0.8	0.78± 0.18
Cellotriose		(1.74 ± 0.33) × 10^−6^	−7.9	−7.1 ± 0.31	0.8	0.77 ± 0.02
Cellotetraose		(0.75 ± 0.03) × 10^−6^	−8.35	−16.6 ± 0.75	8.25	0.45 ± 0.02
Laminaribiose		(22.1 ± 0.93) × 10^−6^	−6.35	−3.14 ± 0.16	2.94	1.03 ± 0.02
Laminaritriose		(3.41 ± 0.01) × 10^−6^	−7.46	−3.66 ± 0.02	3.8	0.9 ± 0.01
Laminaritetraose		(5.6 ± 2.12) × 10^−3^	−5.43	−0.15 ± 0.12	5.29	ND

G4G3G: Glc-β-1-4-Glc-β-1-3-Glc, and G4G4G3G: Glc-β-1-4-Glc-β-1-4-Glc-β-1-3-Glc. ND-not determined. The *Bp*SBP_MLG_ did not bind with maltose, gentiobiose and lactose.

**Figure 4:**
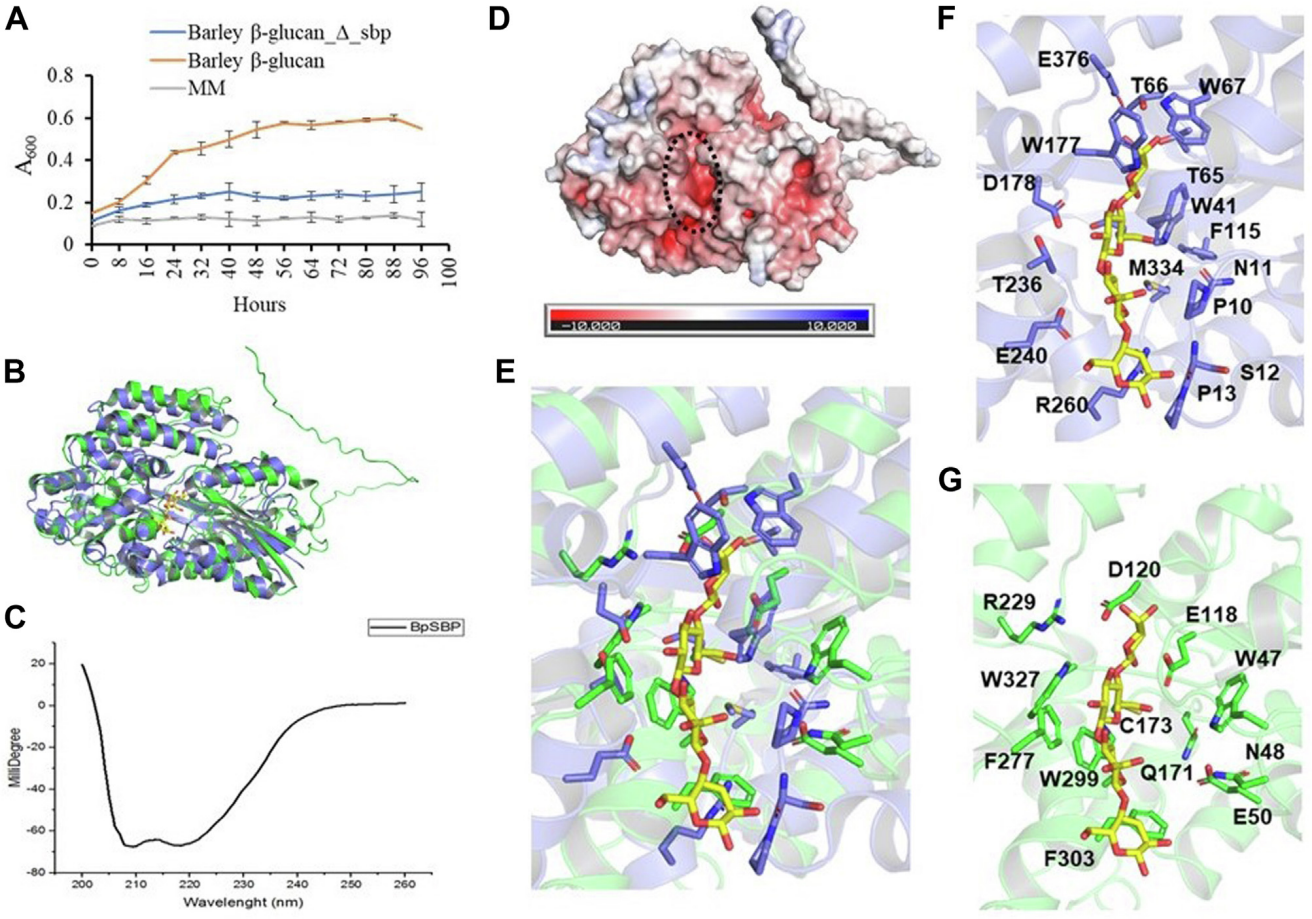
**Growth of mutant *B. producta*_Δsbp on barley β-glucan and structure prediction of *Bp*SBP**_**MLG**_**.***A*, the growth curve of *B. producta* and mutant *B. producta_*Δ*sbp* on a minimal medium containing 1% barley β-glucan. *B*, structural comparison of the *Bp*SBP_MLG_ model (*green*) and the crystal structure of *Thermus thermophilus* β-glucoside-binding protein βGlyBP (sequence similarity, 20%) in complex with cellotetraose (PDB 7C68, *slate blue*). Cellotetraose is shown as a *yellow stick* model. *C*, circular dichroism spectrometry of *Bp*SBP_MLG_. *D*, electrostatic potential of surface model of *Bp*SBP_MLG_ generated by AlphaFold2. The putative sugar-binding site of *Bp*SBP is indicated as a *dotted ellipse*. *E*, overlay structure sugar-binding sites of βGlyBP and *Bp*SBP_MLG_. *E–G*, the sugar-binding sites of βGlyBP (*F*) and *Bp*SBP_MLG_ (*G*) and their superposition (*E*). cellotetraose (*thin yellow stick*) of PDB 7C68 is superimposed to *Bp*SBP_MLG_.

In order to better understand the importance of the *Bp*SBP_MLG_ in the utilization of β-linked oligosaccharides, a targeted gene deletion strategy was first time employed for *B. producta*. Adapting the strategy previously developed for *Bifidobacterium longum* (48) (Fig. S11), we used a double-crossover markerless system for the deletion of the *Bp*SBP_MLG_-encoding *bp1316* gene in the *B. producta* genome. Following confirmation of gene deletion, we observed that *B. producta* ΔSBP showed significant retardation in growth on a minimal medium containing 1% barley β-glucan as compared to the wild-type strain (Fig. 4*A*). The data confirm the central role of *Bp*SBP_MLG_ in enabling *B. producta* growth on MLG.

Various attempts to crystallize the recombinant *Bp*SBP_MLG_ were performed in order to determine the molecular basis for the oligosaccharide binding as mentioned in the Supplementary file. However, no crystals were obtained, even after truncation of the flexible N-terminal region of the protein. Instead, AlphaFold2 was employed to predict the three-dimensional structure of *Bp*SBP_MLG_ (31, 39, 40). The generated model adopted a typical SBP fold that consists of two α/β folds (Fig. 4*B*) and was similar to the structures of known sugar-binding proteins classified into cluster D class I (D-I subcluster) (49). The three-dimensional structures of some β-glucoside-binding proteins have been reported (50, 51, 52), and they are classified into the C-II or D-I subclusters. The CD spectrum also supported that this protein contains predominantly α-helices (Fig. 4*C*). The β-glucoside-binding protein (β-GlyBP) of *Thermus thermophilus* belongs to the D-I subcluster and binds β-(1,2), β-(1,3), β-(1,4) and β-(1,6)-linked glucooligosaccharides (52). Based on the surface electrostatic potential, the cleft between two α/β domains is negatively charged and is predicted to be the ligand-binding site (Fig. 4*D*). Although *Bp*SBP_MLG_ has only 20% sequence similarity to βGlyBP, the overall structures and positions of the sugar-binding sites are similar (Fig. 4, *B* and *D*). The amino acid residues that bind the ligands in βGlyBP were not conserved in *Bp*SBP_MLG_, but several aromatic residues were found in the putative binding site of *Bp*SBP_MLG_ (Fig. 4, *E* and *G*). The amino acid residues in the predicted sugar-binding site are highly conserved between *Bp*SBP_MLG_ and its relatively close orthologues (Fig. S12).

### Cytoplasmic digestion of mixed-linkage oligosaccharides by phosphorylases

Next, we focused on the cytoplasmic degradation of oligosaccharides trapped and imported by the*Bp*SBP_MLG_ and its associated ABC transporter. Locus-associated gene *bp1314* was predicted to encode a cellodextrin phosphorylase (CDP) belonging to CAZy family GH94. The recombinant *Bp*GH94_MLG_ showed maximum activity between pH 6.5 to 7.4 in HEPES buffer at 37 °C using 10 mM inorganic phosphate and cellotriose (Fig. S13). The *Bp*GH94_MLG_ did not show any phosphorolysis activity against laminaribiose, laminaritriose, cellobiose, gentiobiose, maltose, and sucrose (Fig. S14, *A*–*C*). The *Bp*GH94_MLG_ cleaved cellotriose and G4G3G, suggesting that it is a β-(1,4)-glucose phosphorylase (Fig. S14, *A* and *B*). It catalyzed phosphorolysis of barley β-glucan and mainly produced laminaribiose, cellobiose, and glucose-1-phosphate (Glc-1-P) (Fig. S14, *D*–*E*).

**Figure 5:**
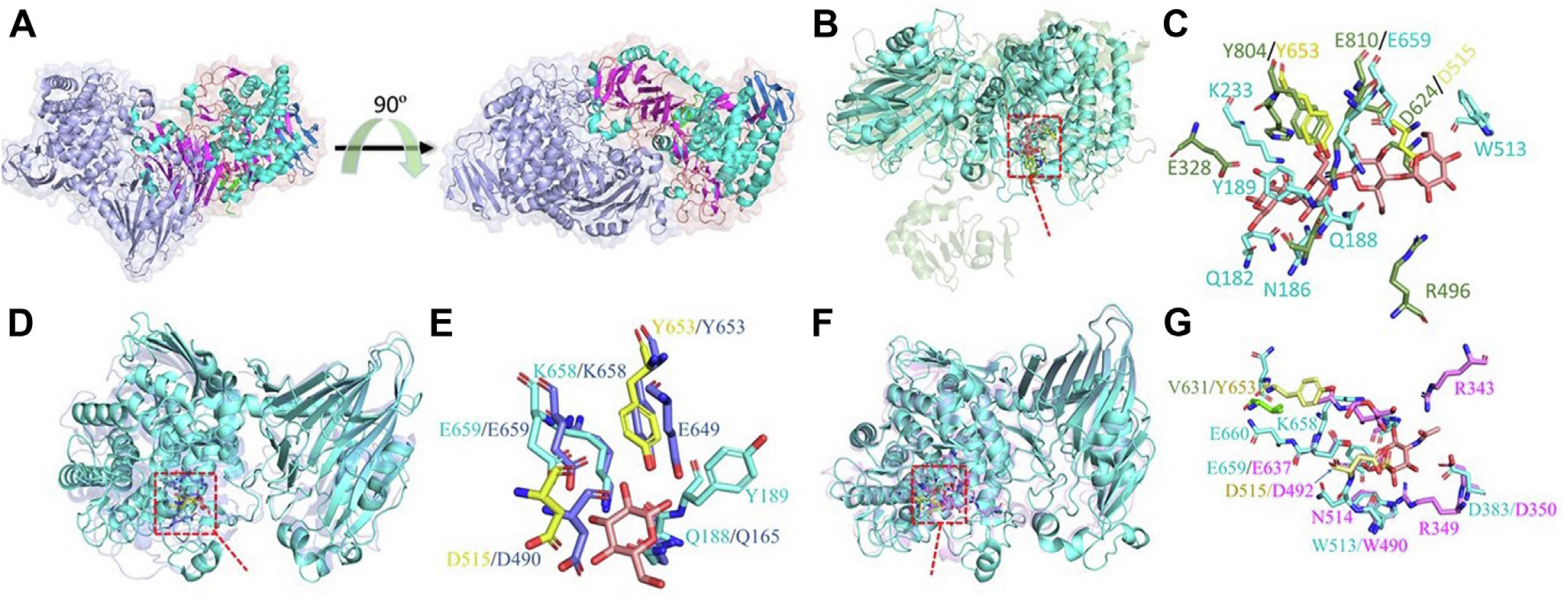
**Crystal structure of *Bp*GH94**_**MLG**_**and comparison between other GH94.***A*, the crystal structure of *Bp*GH94_MLG_ was determined to a resolution of 2.3 Å revealing two copies of the protein per asymmetric unit forming a clear dimer (N-terminal - *green*; loop- *orange*; α-helical- *cyan*; β-sheet- *magenta*; C-terminal- *blue*). *B*, superimposed *Bp*GH94_MLG_ on cellodextrin phosphorylase (PDB id: 5NZ8) and a *red box* highlighting catalytical domain (*Bp*GH94_MLG_ - *cyan* and 5NZ8-*green*). *C*, identifying active residues of the *Bp*GH94_MLG_ by comparing residues of the active site of 5NZ8 present around ligand (cellotetraose) in 3 Å vicinity (*Bp*GH94_MLG_ - *cyan*, 5NZ8-*green*, D515 and Y653 of *Bp*GH94_MLG_ - *yellow* and *ligand-red*). *D*, superimposed *Bp*GH94_MLG_ on cellobiose phosphorylase (PDB id: 2CQS), and a *red box* highlighting catalytical domain (*Bp*GH94_MLG_ - *cyan* and 2CQS- *blue*). *E*, identifying active residues of the *Bp*GH94_MLG_ by comparing residues of the active site of 2CQS present around ligand (glucose) in 3 Å vicinity (*Bp*GH94_MLG_ - *cyan*, 2CQS- *blue*, D515 and Y653 of *Bp*GH94_MLG_ - *yellow* and *ligand-light red*). *F*, superimposed *Bp*GH94_MLG_ on chitobiose phosphorylase (PDB id: 1V7W), and a *red box* highlighting catalytical domain (*Bp*GH94_MLG_ - *cyan* and 1V7W- *magenta*). *G*, identifying active residues of the *Bp*GH94_MLG_ by comparing residues of the active site of 1V7W present around ligand (N-acetylglucosamine) in 3 Å vicinity (*Bp*GH94- *cyan*, 1V7W- *magenta*, and *ligand-light red*). These structures were processed in PyMol software.

Kinetic parameters for phosphorolysis of cellotriose, G4G3G, and G4G4G3G showed that K_m_ was about 1.85-fold lower and 0.75-fold higher for G4G3G and G4G4G3G, respectively, when compared to cellotriose (Fig. S15 and Table 3). Higher k_cat_/K_m_ was observed for G4G4G3G by 2.4 and 1.6-fold when compared to cellotriose and G4G3G, respectively. Similarly, k_cat_ and K_m_ values of the *Thermosipho africanus* TCF52 B (*Ta*CDP) for cellotriose were reported as 0.28 s^−1^ and 0.094 mM, respectively (53). Compared to *Bp*GH94_MLG_, low phosphorolysis kinetic efficiency was reported for the CDP of *Ruminiclostridium thermocellum* YM4, *Rt*CDP (54), and *Ruminococcus albus, Ra*CDP (55) (Figs. 5 and S2). None of these CDPs cleave cellobiose, but they can generate Glc-1-P from cellotriose. They also accept β-(1,4)-linked glucans with DP ≥2 as acceptors for Glc-1-P-based synthesis (55, 56). Such actions were discrete from characterized β-(1,3)-glucan phosphorylases belonging to GH149, which work on acceptors with DP ≥1 (57). The phosphorolysis kinetic efficiency supports its role in the utilization of oligosaccharides derived from mixed linkage β-glucans.

**Table 3:** Kinetic parameters of oligosaccharides phosphorolytic reaction by *Bp*GH94_MLG_ compared with previously calculated kinetic parameters (∗) of known CDPs, such as *Thermosipho africanus* TCF52B (*Ta*CDP) (53); *Ruminiclostridium thermocellum* YM4 (*Rt*CDP) (54), and *Ruminococcus albus* (*Ra*CDP) (55)

Substrate	K_m_ (mM)	k_cat_ (s^−1^)	k_cat_/K_m_ (s^−1^ mM^−1^)	K_m_ (mM)	k_cat_ (s^−1^)	k_cat_/K_m_ (s^−1^ mM^−1^)	K_m_ (mM)	k_cat_ (s^−1^)	k_cat_/K_m_ (s^−1^ mM^−1^)	Km (mM)	k_cat_ (s^−1^)	k_cat_/K_m_ (s^−1^ mM^−1^)
	*Bp*GH94_MLG_			*Ta*CDP∗			*Rt/Ct*CDP∗			RaCDP∗		
Cellobiose		NA		0.120	0.23	1.1		NA			NA	
Cellotriose	0.028 ± 0.004	0.67 ± 0.021	23.9	0.094	0.28	2.9	0.81	4	5	6.06	76.2	12.6
G4G3G	0.052 ± 0.005	1.86 ± 0.022	35.7		NA			NA			NA	
G4G4G3G	0.021 ± 0.002	1.21 ± 0.014	57.6		NA			NA			NA

The crystal structure of *Bp*GH94_MLG_ was determined to a resolution of 2.3 Å (Table S1), revealing two copies of the protein molecules per asymmetric unit, forming a clear dimer (Fig. 5*A*). It contains a complex four-domain architecture, a β-sandwich domain extends into a smaller two α-helical linker domain connecting to an (α/α)_6_-barrel domain before terminating in a smaller β-sandwich domain (Fig. 5*A*). The top hit from a DALI analysis indicates a few close structural homologous in the PDB, giving Z scores in the range of 44.6 to 30.7 (58). For instance, chitobiose phosphorylase, ChBP, (PDB id- 1V7V/1V7W, Z scores 44.6), cellobiose phosphorylase, CBP (PDB ID- 2CQS, Z scores 43.4), laminaribiose phosphorylase, LBP (PDB ID- 6GGY, Z scores 32.6), and cellodextrin phosphorylase, *Rt*CDP (PDB ID- 5NZ7/5NZ8, Z scores 30.7) shared 36%, 33%, 18%, and 23% sequence identify to *Bp*GH94_MLG,_ respectively.

In contrast to *Rt*CDP (Fig. 5*B*), *Bp*GH94_MLG_ lacks an additional domain at the N-terminus end that is made up of mixed αβ-structures (56). Although amino acid sequence similarities between *Rt*CDP, ChBP, and CBP are limited, these can be structurally aligned with *Bp*GH94_MLG_. Structural alignment with *Rt*CDP, CBP, and ChBP suggested that the active site of *Bp*GH94_MLG_ is present at one end of the (α/α)_6_-barrel of the catalytic domain (Fig. 5, *C*, *E*, and G). Similar to *Rt*CDP, CBP, and ChBP, a conserved residue, Asp515, is expected to act as a general acid catalyst. Comparing these phosphorylases highlights that *Bp*GH94_MLG_ has a Y653 residue corresponding to Y804, and Y653 of *Rt*CDP, and CBP, respectively (Fig. 5, *D* and *F*). This tyrosine is replaced with valine (V631) in the ChBP, a crucial residue for determining substrate specificity for chitobiose (59). The architecture of the active site pocket of *Bp*GH94_MLG_ is very similar to *Rt*CDP, further suggesting that this enzyme is suitable for cleaving β-(1,4) linkages. Furthermore, a comparison of the active pocket of *Bp*GH94_MLG_ and other GH94 suggested that *Bp*GH94_MLG_ has an open active site pocket when compared to ChBP and CBP for accommodating short and longer chain substrates, such as G4G3G and G4G4G3G (Fig. S16).

### Cytoplasmic digestion of mixed-linkage oligosaccharides by glycoside hydrolases

The human gut is a very complex system where redundancy for expressing a similar type of enzyme within a bacterium is frequently observed for glycan utilization. Therefore, we performed a proteomic analysis in order to identify enzymes that may be involved in the utilization of oligosaccharides produced by *Bp*GH16_MLG_. Through proteomics, we identified two GH3s (*Bp*4420/*Bp*GH3-AR8_MLG_ and *Bp*8539/*Bp*GH3-X62_MLG_) that were upregulated when *B. producta* was grown on barley β-glucan (Fig. 1*D*). Both enzymes contain no signal peptide when analyzed with SignalP, v. 4.0, suggesting that they are cytoplasm-localized. Amino acid sequences were analyzed with NCBI Conserved Domain, suggesting that AR8_MLG_ and X62_MLG_ belong to the GH3 family (34).

*Bp*GH3-AR8_MLG_ and *Bp*GH3-X62_MLG_ both showed maximum activity in a phosphate buffer of pH 7.4 at 37 °C using pNP-β-D-Glc as a substrate (Fig. S17). Both enzymes cleaved β-(1,4), β-(1,3), and β-(1,6)-linked glucose from various oligosaccharides, including G4G3G and G4G4G3G (Fig. S18), with very similar kinetics parameters for each of these oligosaccharides (Table 4 and Fig. S19). The K_m_ values of both enzymes increased with increasing DP of cello-oligosaccharides and MLG-oligosaccharide, while those parameters remained similar with increasing DP of laminari-oligosaccharides. TLC profiles of enzyme-degraded products demonstrated that both enzymes sequentially cleaved glucose from the non-reducing end of cello-oligosaccharides, laminari-oligosaccharides, and MLG-oligosaccharide (Figs. S20 and S21). Both enzymes showed somewhat higher catalytic efficiency than recently reported for the MLG-degrading *Bo*GH3_MLG_ (8). For example, *Bp*GH3-AR8_MLG_ and *Bp*GH3-X62_MLG_ show k_cat_/K_m_ 71 and 67, 73 and 73.6 s^−1^ mM^−1^ for G4G3G and G4G4G3G, as compared to k_cat_/K_m_ 47.4 and 41.8 s^−1^ mM^−1^ for G4G3G and G4G4G3G for *Bo*GH3_MLG_, respectively. *B. ovatus* producing *Bo*GH3_MLG_ (BACOVA_02745) shared 27% sequence similarity with *Bp*GH3-X62_MLG_ and *Bp*GH3-AR62_MLG_.

**Table 4:** Kinetics parameters for hydrolyzing various natural substrates by *Bp*GH3-AR8_MLG_ and *Bp*GH3-X62_MLG_

Substrate	Kinetics parameters for *Bp*GH3-AR8_MLG_			Kinetics parameters for *Bp*GH3-X62_MLG_		
	K_m_ (mM)	k_cat_ (s^−1^)	k_cat_/K_m_ (s^−1^ mM^−1^) PAHBAH assay	K_m_ (mM)	k_cat_ (s^−1^)	k_cat_/K_m_ (s^−1^ mM^−1^) PAHBAH assay
G4G3G	0.24 ± 0.04	17 ± 0.3	71	0.23 ± 0.04	17 ± 0.3	73
G4G4G3G	0.3 ± 0.04	20 ± 0.3	67	0.25 ± 0.05	18 ± 0.3	74
Cellobiose	0.20 ± 0.03	8 ± 0.1	37	0.2 ± 0.03	8 ± 0.01	38
Cellotriose	0.26 ± 0.03	15 ± 0.2	58	0.26 ± 0.03	15 ± 0.2	59
Cellotetraose	0.47 ± 0.06	20 ± 0.2	42	0.45 ± 0.06	19 ± 0.3	43
Laminaribiose	0.24 ± 0.03	12 ± 0.2	48	0.24 ± 0.02	12 ± 0.2	48
Laminaritriose	0.23 ± 0.05	12 ± 0.2	52	0.24 ± 0.04	12 ± 0.2	51
Gentiobiose	0.24 ± 0.04	14 ± 0.2	58	0.24 ± 0.03	14 ± 0.2	58

Blanks were applied to all reaction mixtures (substrate and buffer without enzyme) and processed similarly to test samples. The blank value was subtracted from the test value in PAHBAH reducing sugar assay.

Abbreviation: PAHBAH, *p*-hydroxybenzoic acid hydrazide.

### Bioinformatics analysis

**Figure 6:**
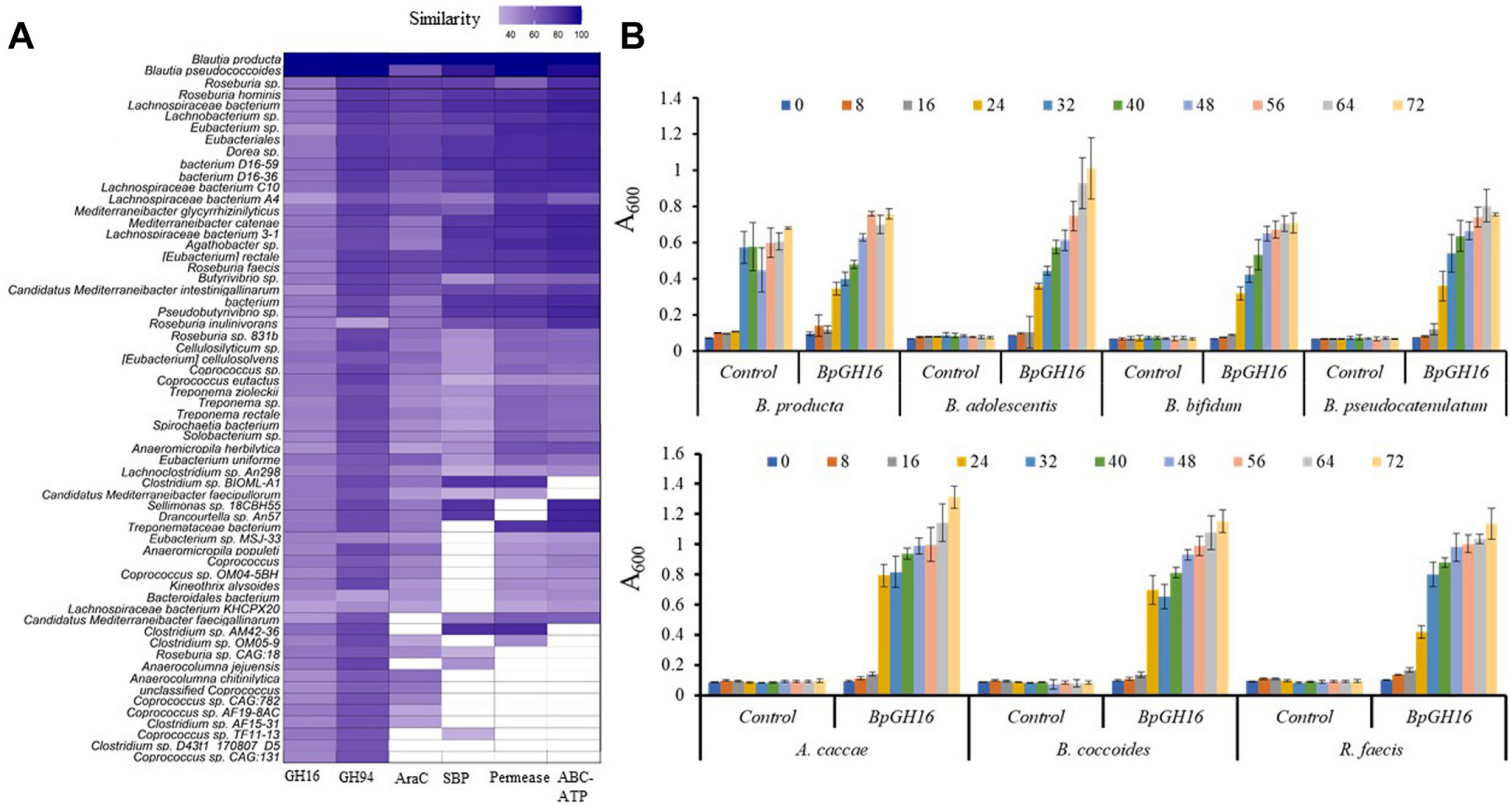
**Prevalence of barley β-glucan utilizing locus across human gut microbiota and Growth patterns of some human gut bacteria.***A*, prevalence of all the six proteins of *B. producta* shown by histogram. The X-axis includes all six proteins and Y-axis includes all species identified from the sequence homology searches. The color of each *rectangular* for each member is corresponding to the percentage similarity. The image generated using the R script was further rearranged manually based on protein occurrence and similarity. *B*, growth patterns of different human gut bacteria on barley β-glucan. Bacteria were grown on a minimal medium containing 1% barley β-glucan in the absence or presence of *Bp*GH16_MLG_. Growth patterns of all human gut bacteria were determined in a period of 72 h, and growth was measured in an alternative of 8 h spectrophotometrically. *B. producta* only grew on a minimal medium containing 1% barley β-glucan in the absence of *Bp*GH16_MLG_, while other bacterial strains showed dependency on *Bp*GH16_MLG_ for generating oligosaccharides. The generated oligosaccharides seem to be appropriate carbon sources for all six selected strains.

The prevalence of orthologues of the six proteins encoded by *B. producta* MLG degradation locus was assessed in other bacteria. Homology searches of sequences available in the NCBI NR database with the criteria of having at least 75% query coverage with at least 30% sequence similarity for each of the six proteins are presented in Figure 6*A*. It is evident that the locus in question is widely found among different genera of the Lachnospiraceae family, such as *Roseburia* and *Clostridium* sp. Furthermore, we identified homologues but variable similarity of all the six proteins in some species of *Butyrivibrio* sp., *Bacterium* D16-59*, Bacterium* D16-36*, Agathobacter* sp.*, Candidatus Mediterraneibacter intestinigallinarum, Cellulosilyticum* sp.*, Coprococcus* sp.*, Dorea* sp*., Eubacterium uniforme, Eubacterium* sp. MSL-33, *Eubacteriales*, *Roseburia* sp., and *Treponema* sp. (Fig. 6*A*). *Blautia pseudococcoides* has homologs with ∼90% sequence similarity for five proteins and 67% query coverage with AraC family protein. The comparative analysis revealed that a total of 37 out of 62 species have homologs of all six proteins. Interestingly, *Bp*SBP_MLG_ and *Bp*GH94_MLG_ in many bacteria showed high sequence identities, suggesting that members of Lachnospiraceae are evolutionarily adapted to utilize MLG oligosaccharides in order to survive in the nutrient-competitive environment of the gut.

### Utilization of *Bp*GH16_MLG_-released oligosaccharides by other beneficial bacteria

We next investigated the importance of *Bp*GH16_MLG_ in supporting the growth of human gut-beneficial bacteria on oligosaccharides generated from barley β-glucan. Some human gut Gram-positive bacteria belonging to the Lachnospiraceae were used for this analysis as it was observed from bioinformatic analysis that the locus of *B. producta* has a commonality with these bacteria. As a representative of commonality and other members of Lachnospiraceae, we selected *Roseburia faecis* JCM 17581^T^/M72, and *Blautia coccoides* JCM 1395^T^ and *Anaerostipes caccae* JCM 13470^T^, respectively. Additionally, we used species of *Bifidobacterium* (*Bifidobacterium pseudocatenulatum* JCM 1200^T^, *Bifidobacterium adolescentis* JCM 1275^T^, and *Bifidobacterium bifidum* JCM 1254) that are commonly associated with health promotion (60, 61). All selected bacterial members started to grow and reached the stationary phase after 16 h and 56 h of incubation. Overall, all members showed robust growth on oligosaccharides generated by *Bp*GH16_MLG_ from barley β-glucan and compared to the parent polysaccharide (Fig. 6*B*). Only *B. producta* showed growth on barley β-glucan (Fig. 6*B*) as compared to other bacteria (Fig. 6*B*). We visited the genomes of these selected strains where *R. faecis* showed 40 to 62% protein sequence similarity with locus-encoded genes (Fig. 6*A*). Despite having these sequence similarities, the strain could not grow on a minimal medium with 1% barley β-glucan, suggesting that *R. faecis* encoded GH16 may have different functional properties. *B. pseudocatenulatum* JCM 1200^T^ has 40% (gene id 2562617145) and 46% (gene id 2563201727) homolog with *Bp*GH3-X62_MLG_ and *Bp*GH3-AR62_MLG,_ respectively. *B. adolescentis* JCM 1275^T^ has 41% (gene id 639633010) and 47% (gene id 639764571) homolog with *Bp*GH3-X62_MLG_ and *Bp*GH3-AR62_MLG,_ respectively. *B. coccoides* JCM 1395^T^ showed protein sequence similarity with 99% (gene id 2799456949 and 2799455541 with *Bp*GH3-X62_MLG_ and *Bp*GH3-AR62_MLG,_ respectively). *B. bifidum* JCM 1254 and *A. caccae* JCM 13470^T^ do not have homologous MLG utilization proteins to *B. producta* but possess β-glucosidases belonging to the GH1, GH2 and GH3 families.

Since *B. producta* grew on barley β-glucan, it was thought that it can produce short-chain fatty acids (SCFAs) after fermenting digested barley β-glucan. Thus, they were grown in 96 well plates containing minimal medium with 1% barley β-glucan, and produced SCFAs from culture supernatant were measured through HPLC. Peaks were confirmed by the retention time of standards. Acetate, propionate, and butyrate were present in the culture supernatant in the ratio of 6.5: 2.5:1, respectively (Fig. S22). In general, acetate, propionate, and butyrate are present in a molar ratio of 60:20:20 in the GIT, accounting for >95% of the SCFA content (62). However, this ratio varies based on the carbon source available to microbial communities (63).

## Discussion

Gram-negative bacteria, such as *B. ovatus* ATCC 8483, encode all necessary genes for MLG-utilization in a single locus, including those for periplasmic utilization of released and imported oligosaccharides (8). In contrast, in Gram-positive bacteria such as *B. producta* and other strains only some of the necessary genes for MLG utilization are co-located (Fig. 6*A*). The gpPUL lacks the SusC and SusD proteins as present on the Gram-negative PULs paradigm. The gpPUL also deprives of SGBP (as it is present in Gram-negative bacteria, such as *B. ovatus* ATCC 8483) and a function of SGBP is often replaced by a CBM, associated with endoacting GHs linked to gpPULs (26, 27). The present study further extends the finding of Sheridan *et al.* (26), who predominantly identified the gpPULs in *Roseburia/E. rectale* pangenome, by virtue of identifying gpPUL in various genera of Gram-positive bacteria (6A). As per definition of gpPUL, *B. producta* MLG-PUL consisted of a transcriptional regulator, one polysaccharide-degrading enzyme and a carbohydrate transport system.

**Figure 7:**
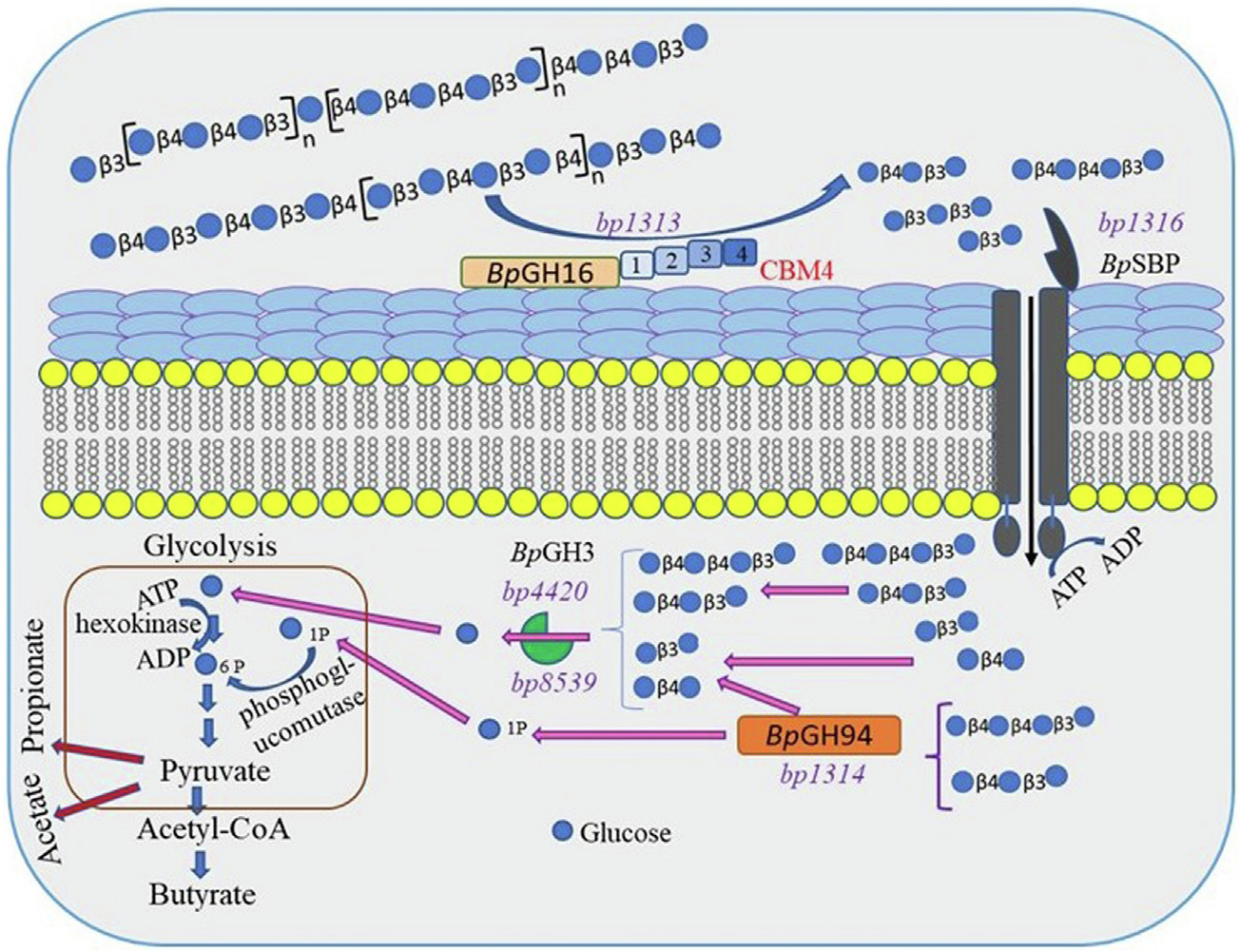
**Overall digestion of barley β-glucan and lichenan by the *B. producta*.** The *Bp*GH16_MLG_ digests barley β-glucan and lichenan in the gut environment and releases various oligosaccharides. The resulting oligosaccharides are sensed by *Bp*SBP_MLG_ and transported into the cytoplasm by ABP transporter component expressed by the locus. Imported oligosaccharides can be processed through *Bp*GH94_MLG_. Biochemical and crystal investigations suggested that *Bp*GH94_MLG_ produced disaccharides and α-D-Glc-1-P as limit digest products from imported oligosaccharides. Proteomics analysis on barley β-glucan grown *B. producta* culture identified *Bp*GH3-AR8_MLG_ and *Bp*GH3-X62_MLG._ This analysis suggested that those generated disaccharides or/and imported mixed-linkage oligosaccharides can be digested by *Bp*GH3-AR8_MLG_ and *Bp*GH3-X62_MLG_ and converted into glucose. Biochemical analyses revealed that these enzymes are highly efficient in utilizing β-(1,3) (1,4), and (1,6) linked oligosaccharides. Produced glucose and glucose-1 phosphate enter into metabolic pathways that ferment into acetate, butyrate, and propionate. These SCFAs are analyzed by HPLC using a Hi-Plex H column (300 × 7.7 mm; 8 μm particle size, Agilent Tech).

Similar to *Bo*GH16_MLG_ (8) and other organisms with PULs associated GH16s (23, 31, 64), *Bp*GH16_MLG_ is present on the outer cell membrane and cleaves different structural types of β-glucans (Fig. 7). When fed barley MLG, *Bp*GH16_MLG_ produces G4G3G and G4G4G3G as limit digest products, as does the corresponding *B. ovatus* enzyme, *Bo*GH16_MLG_. SGBP provides an access for barley MLG to *Bo*GH16_MLG_ (∼30 kDa). In the case of Gram-positive bacteria, CBM4 domains are known to bind β- (1, 3) and β-(1,3/4) glucans (65). Therefore, four CBM4 domains of *Bp*GH16_MLG_ may collectively offer an advantage for binding solid plant material in the gut environment.

*Bp*SBP_MLG_ is identified as a bespoke transporter to trap β-(1,3)- and β-(1,4)-linked oligosaccharides in a gut environment. Mannan-binding SBP (*Ri*MnBP) of *Roseburia intestinalis* has been reported to bind mannan-derived oligosaccharides at μM levels (27). The primary parameters influencing the selective absorption of oligosaccharides by Gram-positive bacteria are SBPs of ABC transporters (46, 66, 67). The ABC transporter of *Roseburia inulinivorans* was previously observed to overexpress by about ten fold in the presence of inulin as compared to fructose (68). *Ri*MnBP was approximately overexpressed by sixfold in the presence of konjac glucomannan and spruce acetylated galactoglucomannan (AcGGM) when compared to glucose in transcriptomic analysis (27). *Ri*MnBP was highly abundant (34-fold) in proteomic data of AcGGM grown culture of *R. intestinalis.* In line with these previous studies, *Bp*SBP_MLG_ was highly abundant in proteomic data -76-fold higher in barley β-glucan grown *B. producta* culture as compared to glucose grown culture. The importance of *Bp*SBP_MLG_ was confirmed by genetic deletion, where the mutant bacterium showed retarded growth on barley β-glucan.

Further, the oligosaccharide specificity of *Bp*SBP_MLG_ is aligned with cytoplasmic *Bp*GH94_MLG_ and GH3 (*Bp*GH3-AR8_MLG_ and *Bp*GH3-X62_MLG_). It highlights that *B. producta* ATCC 27340 can employ two distinct mechanisms (Fig. 7) given that *Bp*GH94_MLG,_ *Bp*GH3-AR8_MLG_ and *Bp*GH3-X62_MLG_ have approximately similar kinetic efficiencies for utilizing MLG-derived oligosaccharides (Tables 3 and 4; Fig. 5). In the first pathway, *Bp*GH94_MLG_ phosphorolyses β-(1,4)-linked oligosaccharides in the cytoplasm and produces cellobiose, laminaribiose and Glc-1-P as final products from G4G3G and G4G4G3G (Fig. 7). The resulting Glc-1-P directly enters into glycolysis, saving a molecule of one ATP overall. Alternatively, imported oligosaccharides can be hydrolyzed to monosaccharides by *Bp*GH3-AR8_MLG_ and *Bp*GH3-X62_MLG_, forming a second efficient pathway (Fig. 7). Orthologues of *Bp*GH3-AR8_MLG_ and *Bp*GH3-X62_MLG_ were observed in our previous work relating to the utilization of mixed linkage β-glucans in *Bacteroides uniformis* JCM 13288 (69).

Previously, Biddle, Stewart, Blanchard, and Leschine (70) performed a survey of the fibrinolytic potential among Lachnospiraceae and found the presence of various CAZymes families, CBM and ABC transporters for degrading pectin and hemicellulose. Indeed, our bioinformatic analysis highlights that barley β-glucan utilizing capability is prevalent among Lachnospiraceae and *Treponema* in the HGM (Fig. 6*A*). In particular, homologs of *Bp*GH94_MLG_ and *Bp*SBP_MLG_ are commonly present among members of Lachnospiraceae, the most abundant family of *Firmicutes* in the human colon, suggesting that they are evolutionarily adapted to use oligosaccharides generated in the gut environment.

We further explored the possible roles of *Bp*GH16_MLG_ in cross-feeding systems with different glucans. We indeed demonstrated that oligosaccharides generated from barley β-glucan supported the growth of beneficial human gut bacterial species (Fig. 6*B*), which are normally associated with a healthy gut (71). For instance, *Bifidobacteria* demonstrate the ability to prevent and/or treat colorectal cancer and inflammatory bowel disease (61), while *B. bifidum* or *B. adolescentis* are known to alleviate low blood glucose concentration and gut microbiota disorders (72, 73) and the adult-dominant species *B. pseudocatenulatum* may prevent type 2 diabetes (74, 75). *B. coccoides* JCM1395^T^ is recently suggested to be used for tumor-targeted live bacterial therapeutics (76). Bacterial metabolites such as SCFAs are essential nutrients for human survival and mitigating various diseases in the human gut, including inflammatory bowel diseases (77). *Roseburia* species belong to butyrate-producing bacteria and can ameliorate alcoholic fatty liver (78). *A. caccae* has been recognized as butyrate-producing bacteria (79), thus enhancing the production of butyrate. Similarly, *B. producta* ATCC 27340 produces acetate, propionate, and butyrate, suggesting that the strain not only supports beneficial bacteria growth through a cross-feeding system but also promotes the health status of the host through producing SCFAs. Together with our observations, this suggests that *Bp*GH16_MLG_ and *B. producta* itself have the potential to support the growth of beneficial bacteria *in vivo*, thus improving human health.

Although MLG positively affects microbial composition by increasing specific bacterial populations and maintaining gut-bacterial homeostasis for healthy individuals (80), much less is known about how beneficial bacteria utilize MLG at the molecular level. The GIT harbors a multifaceted and dynamic population of microorganisms that exert a significant effect on human health. The diet strongly influences the composition of the colonic microbiota and the balance of its metabolic products. Thus, diet presents promising opportunities for maintaining HGM homeostasis and preventing non-communicable diseases (81). In particular, genes of bacterial communities encode highly diverse enzymes for utilizing dietary glycans and act as a secondary genome of the host. A better understanding of the molecular mechanisms supported by these genes offers novel opportunities for intervention to address the dysbiosis of the HGM and prevent associated chronic disorders. Gram-positive bacteria are under-explored in this regard. Herein, we have addressed this gap through a detailed analysis of *B. producta* ATCC 27340 MLG metabolism.

## Experimental procedures

Lichenan from Icelandic moss, barley β-glucan, laminaribiose, laminaritriose, laminaritetraose, laminaripentaose, cellotriose, cellobiosyl-β-D-1,3-glucose (G4G3G) were purchased from Megazyme, Ireland. Gentiobiose and laminarin were purchased from Sigma Aldrich. All NMR analyses were performed at Panjab University, Chandigarh, India. All kinetic parameters were performed with GraphPad Prism.

### Assessment bacterial growth on β-glucans

Active culture of *B. producta* and culture conditions were maintained following the recently reported procedure (31). Once *B. producta* grew, cells were centrifuged, and the obtained supernatant was decanted. A pellet of cells was then washed three times with phosphate-buffered saline (PBS) and washed cells were suspended in 2 ml PBS. 5 μl of cell suspended volume was inoculated in 200 μl of minimal medium containing 1% barley β-glucan or lichenan on 96 well plates. The growth assessment experiments were performed for 96 h, following our recently established protocol (82).

### Total RNA extraction and gene expression analysis

Changes in the transcript expression levels of the β-glucan utilization locus of *B. producta* were performed by RT-qPCR. *B. producta* was initially cultured in 5 ml of GAM for 24 h and cells were spun down by centrifugation and washed three times with sterile PBS. Afterward, 20 μl of 1 ml suspension of bacterial cells was cultured in a 10 ml minimal medium containing 1% (w/v) barley β-glucan, 3 ml of bacterial cultures were collected at mid-log phase (A600 about 0.6) by centrifugation at 15,000*g* and resuspended in 300 μl of a solution consisting of 1 mM EDTA, 0.5% SDS and 0.02 M sodium acetate (pH 5.5). Immediately, 300 μl saturated phenol with citrate buffer (pH 4.3, P4682- Sigma Aldrich) was added, and the mixed solution was incubated at 60 °C for 5 min with mild shaking. After that, it was centrifuged at 15,000 *g* for 5 min, and the upper aqueous phase was transferred into a new Eppendorf tube. It was mixed with three volumes of chilled ethanol and incubated for 30 min at −80 °C. The precipitate was then collected by centrifugation at the same speed, the pellet was washed with 70% ethanol twice, and the final white residue was dissolved in 100 to 500 μl of distilled water. The concentration of total RNA was calculated by a spectrophotometer at 260 nm. If a residual amount of DNA was observed, DNase-1 treatment was also given, following the manufacturer's instructions (AMPD1, Sigma Aldrich).

About 2 μg total RNA was immediately used for reverse transcription using AccuScript High Fidelity cDNA Synthesis Kit (Agilent). RT-qPCR was performed in a 96-well plate on a CFX96 qPCR system (BioRad) using SYBR Green Master (BioRad), using the primers mentioned in Table S2. The qPCR reactions were executed in 10 μl, consisting of 5 μl of SYBR green mix, 30 ng of cDNA, and one pmol of each primer of a gene of the locus or 16S rRNA (Table S2). The reaction condition was 95 °C for 10 min, followed by 40 cycles of 95 °C for 10 s, 55 °C for 10 s, and 72 °C for 10 s. The 2^ˆ^-ΔΔCt method calculated the expression of each gene and normalized data to 16S rRNA transcript level. Finally, the expression level of each gene was calculated as fold change compared with cultures of a minimal medium containing 1% glucose. Three technical replicates were used for bar generation of each gene expression; each bar represents average values and error bars indicate the standard errors of three technical replicates.

### Proteomics

*B. producta* ATCC 27340 was anaerobically grown at 37 °C overnight, cells were collected by centrifugation (3000*g* at room temperature, RT) and washed thrice with 5 ml PBS. After that, cells were resuspended into 2 ml PBS and 5 μl of it was inoculated into filtrated (0.2 μm filters) 10 ml minimal medium containing 1% barley β-glucan or glucose. The strain was grown to the mid-late exponential phase (A595 ∼ 0.5–0.6) in two biological replicates. Cells were collected by centrifugation (5000*g*) for 5 min at 4 °C, washed twice with ice-cold PBS, and resuspended in a 2 ml lysis buffer (50 mM HEPES containing protease inhibitors, pH 6.5). For proteome analysis, cells were lysed by a bead beater (three cycles of 60 s on MP Biomedicals, CA) using glass beads (acid washed ≤ 106 μm). The lysate was centrifuged (15,000*g*, 15 min at 4 °C), and the supernatant was collected. Procedures for the precipitation of protein, trypsin digestion, and analysis of generated peptides have been mentioned in the Supplementary file.

### Cloning of identified genes and recombinant production of the corresponding enzymes

A predicted β-glucan utilization locus of *B. producta* was identified from the genome (33), and genes of the locus were retrieved based on gene ID- 2515951313 to 2515951318 of IMG genome ID- 2515154176 using the Integrated Microbial Genomes (IMG) and Metagenomes (IMG/M) portal. Cellular location based on the signal peptide of proteins encoded by β-glucan utilization locus was predicted using SignalP, v. 4.0 online server (36). Subsequently, appropriate primers were designed (Table S3) after excluding lipidated and signal peptide sequences. The lipidated amino acid of a protein was screened with the LipoP 1.0 server (35). Restriction sites of primers were wisely selected after investigating that no restriction sites of selected restriction enzymes were present within the gene. As previously reported, the amplification of genes was achieved by PCR, and products were purified (31). Then, purified PCR products were cloned into pET28a(+) plasmid, having N-terminal (His)6-tag. Cloned constructs were transformed into *E. coli* TOP10 cells. Screening for positive clones and plasmid preparation was performed. After checking the genetic integrity of all clones, cloned constructs were transformed into *E. coli* BL21 (DE3) for expression of proteins. Genes of ID- 2515951313 and ID- 2515951314 were synthesized and inserted into pET28a(+) by the GenScript Biotech PTE.LTD

### Expression and purification of enzymes

*E. coli* BL21 cells were cultured in the Terrific broth (TB) (for *Bp*GH16_MLG_) or Luria-Bertani (LB) medium (for all other proteins) containing kanamycin (50 μg/ml) to absorption ∼1.2 or mid-log phase (A600 nm of ∼ 0.6) at 37 °C respectively. Afterward, the expression of proteins was induced by adding 0.4 or 0.5 mM isopropyl β-D-1-thiogalactopyranoside (IPTG) to bacterial cultures. LB medium was further incubated at 22 °C for 16 h. In the case of the TB medium, the protein was induced by incubating at 18 °C for 16 h. Post incubation, bacterial cells were harvested by spinning at 4000*g* and then resuspended in 50 mM HEPES buffer (pH 7.5) with an appropriate EDTA-free protease inhibitor cocktail (Sigma Aldrich, Cat. No. 4693159001). Cells were lysed by ultrasonication, and the supernatant was then obtained by centrifugation at 16,000*g* for 30 min at 4 °C. Obtained supernatant was initially concentrated through Amicon Ultra-15 Centrifugal Filter Unit (10 kDa, Merck). It was then mixed with 20 ml suspension of nickel-nitrilotriacetate (Ni-NTA) resin and incubated for 15 min at 4 °C. Followed by entire resin was filled in a column, and different gradients (10, 50, 100, 150, 200, and 500 mM) of imidazole containing 500 mM NaCl were applied in a column to get a purified form of a protein. Intended proteins were eluted in 50 to 150 mM imidazole, and imidazole was then replaced with 50 mM HEPES buffer (pH 6.5) by Amicon Ultra-15 Centrifugal Filter Unit (10 kDa). A 10% sodium dodecyl sulfate–polyacrylamide gel electrophoresis (SDS-PAGE) was performed to determine the desired protein's molecular weight (Fig. S23). A native-PAGE was also used to assess the dimerization of *Bp*GH94_MLG_ (Fig. S24). The concentration of purified proteins was finally determined by a spectrophotometer using Bradford reagent, and all proteins were then stored at −80 °C till further analysis, having a final concentration of 1 mg/ml.

### Site-directed mutagenesis

The residues R331, L358, E379, D381, E384, W362, and H398 of *Bp*GH16_MLG_ were mutated to Ala by the Q5Site-Directed Mutagenesis Kit (New England Biolabs, UK, catalogue no. E0554S). Wild-type plasmid *Bp*GH16-pET28a(+) was used as a template, and mutagenic forward and reverse primers were developed using NEBaseChanger with subtle modifications (Table S4). A 25 μl PCR cocktail was prepared for mutating each residue. The final concentration of Q5 Hot Start High-Fidelity 2X Master Mix was 1X, each primer was at 0.5 μM, the amount of used template was 20 ng, and the rest of the volume was made up of nuclease-free water. PCR was executed using the following conditions-initial denaturation was performed at 98 °C for 1 min, for each cycle-denaturation, annealing, and extension (72 °C) were set for 10 s, 20 s, and 7 min, respectively. A total of 25 cycles were performed, and the final extension was for 5 min 5 μl PCR products were loaded on 0.8% agarose gel to conform to the amplification of the mutagenic gene. Ligation of PCR products and cleavage of methylated template DNA was then performed using 5 μl KLD reaction buffer and 1 μl KLD enzyme cocktail at RT for 1 h. It was then transformed into *E. coli* TOP-10 cells and plasmids were isolated. The obtained plasmids were sequenced to confirm the mutation at the desired location. Once mutation was confirmed in a gene, the mutant plasmid was transformed into Rosetta (DE3) cells for protein expression. The mutant protein was then expressed as per the protocol mentioned earlier, and kinetics were performed with barley β-glucan.

### Evaluating biochemical properties of enzymes

The optimum pH of *Bp*GH16_MLG_, *Bp*GH94_MLG_, *Bp*GH3-AR8_MLG_, and *Bp*GH3-X62_MLG_ was accomplished in various buffers by incubating reactions at 37 °C for 15 min in a standard reaction mixture volume (100 μl), consisting of 10 μl of 10 mM substrate and recombinant enzyme at a final concentration of 0.1 mg/ml. *p*NP-β-laminaribioside, and *p*-nitrophenyl-β-D-glucopyranoside (*p*NP-β-Glc) were used as a substrate for *Bp*GH16_MLG_ and *Bp*GH3-AR8_MLG_/*Bp*GH3-X62_MLG_, respectively. Different buffers of 50 mM concentration, such as citrate (pH 3–5.5), sodium phosphate (pH 5.7–8), sodium acetate (pH 4–6), Tris-HCl (pH 7–9), and HEPES (pH to 5.1–9) were used. All reactions were terminated by adding 6 volumes of 1 M Na_2_CO_3._ The released amount of *p*-nitrophenol was determined by measuring the absorbance at 410 nm using NUNC 96 well plates on the SpectraMax i3x multi-mode microplate reader. The concentration of released *p*-nitrophenol was calculated accordingly to Beer Lambert law (A = εcl, where ε is the molar extinction coefficient of *p*-nitrophenol at 410 nm, c is the concentration and l is the optical path length in cm) as reported previously (83).

A reaction mixture consisting of 10 μl Glc-1-P (100 mM), recombinant enzyme (final concentration at 0.1 mg/ml), 10 μl cellobiose (100 mM), and 20 μl sodium molybdate (1 M) was used for determining optimum pH for *Bp*GH94_MLG_. For determining the optimal temperature of *Bp*GH94_MLG_, a reaction mixture consisting of 10 μl Glc-1-P (100 mM), recombinant enzyme (final concentration at 0.1 mg/ml), 10 μl cellobiose (100 mM), 20 μl sodium molybdate (1 M), was incubated at 15, 22, 30, 37, 45 and 55 °C for 30 min. Then, 25 μl of the reaction mixture was mixed with 75 μl of a color solution (0.24% [w/v] sodium ascorbate dissolved in 0.1 N HCl solution), and it was incubated at RT until color developed. Once the color was developed, the reaction was terminated by adding 75 μl stop solution (2% [w/v] sodium citrate tribasic dihydrate in 2% [v/v] acetic acid). Released inorganic phosphate concentration was determined by determining color absorbance at 620 nm using NUNC 96 well plates. A phosphate release assay was used to measure released inorganic phosphate (84). A standard curve of KH_2_PO_4_ (0–2.5 mM) was made to determine the released amount of inorganic phosphate upon enzymatic reactions.

For determining the optimal temperature of *Bp*GH16_MLG_, standard reactions (100 μl) having recombinant enzyme (final concentration at 0.1 mg/ml) and 10 μl of 10 mM *p*NP-β-laminaribioside were performed at different temperatures (15, 22, 30, 37, 45 and 55 °C) for 15 min using optimized pH. For determining the optimal temperature of *Bp*GH3-AR8_MLG_ and *Bp*GH3-X62_MLG_, standard reactions (100 μl) having recombinant enzyme (final concentration at 0.1 mg/ml) and 10 μl of 10 mM *p*NP-β-Glc were performed at different temperatures (15, 20, 25, 30, 37, 42, 48 and 55 °C) for 30 min using optimized pH.

Initial screening of *Bp*GH16_MLG_ was performed in 100 μl reaction volume containing 10 μl of 10 mM of various oligosaccharides (such as sucrose, maltose, gentiobiose, cellobiose, cellotriose, laminaribiose, laminaritriose, laminaripentaose, and G4G3G) and 10 μl of 10 mg of various polysaccharides (laminarin, lichenan, lentinan, oat β glucan, and barley β-glucan) and recombinant enzyme (final concentration at 0.1 mg/ml) in optimized buffer (pH 5.7) for 2 h at 37 °C. After the appropriate incubation time, the reaction was terminated by boiling (5 min), and generated oligosaccharides were analyzed by thin layer chromatography (TLC). Typically, 1 μl of the reaction mixture was used for TLC analysis. TLC analysis was performed on Silica Gel 60 F254 (Merck), and produced sugars were visualized by spraying TLC with 5% H_2_SO_4_ in ethanol, followed by heating. For determining limit digest products of laminarin, barley β-glucan, and lichenan by the action of the *Bp*GH16_MLG_, reactions were executed at different intervals (2, 4, 8, and 24 h), and products were analyzed by TLC and the matrix-assisted laser desorption ionization time-of-flight mass spectrometry (MALDI-TOF-MS).

Michaelis–Menten kinetic parameters (K_m_, V_max_, and k_cat_) of the *Bp*GH16_MLG_ were determined using a range of concentrations (0–1 mg/ml) of a substrate, such as laminarin, lichenan, oat β-glucan, and barley β-glucan. Reactions were performed in 100 μl volume mixture using recombinant enzyme (final concentration at 0.1 mg/ml) in 50 mM sodium phosphate buffer (pH 5.7) at 37 °C for 1 h of incubation. Then, reactions were terminated by boiling for 5 min. Enzymatic assays were determined with 3,5- dinitrosalicylic acid (DNS) assay (85). In brief, 0.05 g Na_2_SO_3_ and 1 g DNS were mixed in a 50 ml water-containing reagent bottle and stirred about 6 h using a magnetic stirrer. After that, 200 μl phenol was added and made up to 100 ml. A working solution of DNS was prepared by adding 50 μl glucose (20% (w/v) stock) in prepared 100 ml of reagent mixtures and immediately used for experiments without adding NaOH. 80 μl DNS reagent was added to the reaction mixtures and incubated at 100 °C for 10 min, followed by ice-cooling for 10 min. Consequently, recorded the developed color spectrophotometrically at 575 nm (85). A standard curve generated by different concentrations of maltose was used for determining released reducing sugars in each case.

The phosphorolysis activity of *Bp*GH94_MLG_ was determined with various oligosaccharides and MLG (barley β-glucan and lichenan) at the concentration of 10 mM using 100 mM inorganic phosphate and recombinant enzyme (final concentration at 0.2 mg/ml) in 100 mM HEPES buffer (pH 6.5) at 37 °C and various incubation time (2, 4, 8, or 20 h). TLC was used for analyzing enzymatic actions. MALDI-TOF-MS determined the limit-digest products of barley β-glucan and lichenan with *Bp*GH94_MLG_. Kinetics parameters for *Bp*GH94_MLG_ were determined with G4G3G, G4G4G3G and cellotriose, following a *p*-hydroxybenzoic acid hydrazide (PAHBAH) reducing sugar assay (69).

Kinetics parameters for*Bp*GH3-AR8_MLG_ and *Bp*GH3-X62_MLG_ were also determined with natural oligosaccharides, such as cellobiose, cellotriose, cellotetraose, laminaribiose, laminaritriose, gentiobiose, G4G3G and G4G4G3G. Recently reported PAHBAH reducing sugar assay used for kinetic parameters of these enzymes (69). In brief, different concentrations of these oligosaccharides (2 μM–1 mM) were incubated with recombinant *Bp*GH3-AR8_MLG_ and *Bp*GH3-X62_MLG_ (final concentration at 0.1 mg/ml) for 5 to 30 min in 100 mM HEPES buffer (pH 6.5) at 37 °C. 150 μl of a freshly prepared 8:2 mixture of reagent A (50 mM sodium sulfite, 0.3 M 4-hydroxybenzhydrazide and 0.6 M HCl) and reagent B (50 mM trisodium citrate, 10 mM CaCl_2_, 0.5 M NaOH) were added to 50 μl sample. The reaction cocktail was boiled for 10 min at 100 °C. After the reaction was cooled down, the absorbance was recorded at 410 nm using the SpectraMax i3x multi-mode microplate reader. A calibration curve with different concentrations of glucose (0, 2, 4, 8, 16, 40, 80, 100, 200, 300, and 500 μM) was employed for each experiment to calculate the released amount of glucose (reducing end equivalents). The same samples were also used for control measurement, and the control value was subtracted from test reading prior to calculate kinetics parameters. In all cases, the K_m_, V_max_, and k_cat_ were analyzed on GraphPad Prism. Three independent replicates were used for each test. TLC analyses were used for understanding the enzymatic degradation profiles of various oligosaccharides.

### NMR analysis of limit-digest products of *Bp*GH16_MLG_

Barley β-glucan limit digest products of *Bp*GH16_MLG_ were analyzed with ^13^C-DEPT- NMR to confirm the type of linkages in their digested products. 200 mg barley β-glucan was digested by the *Bp*GH16_MLG_ overnight at 37 °C. All generated products were purified with size exclusion chromatography using Toyopearl resin (HW-40) filled column (100 × 25 mm). All eluted products were checked with TLC for chromatographic patterns and were freeze-dried for analysis. NMR was performed on a Bruker Avance Neo 500 MHz spectrometer equipped with a broadband BBFO probe. Chemical shifts (*δ*) were defined in parts per million (ppm) in which the residual solvent signal was demarcated as a reference point for assigning sample peaks. The data processing and analysis were performed with the MestreNova software.

### Circular dichroism spectroscopy

The secondary structure prediction of the solute binding protein (*Bp*SBP_MLG_) was made on a 410 model CD spectrometer (Biologic Science Instrument, AL X 300, MOS 500) using 1 ml cuvette with a 5 mm path length. For all measurements, the *Bp*SBP_MLG_ was diluted to 5 μM in a 10 mM phosphate buffer (pH 7) having 150 mM KCl. Spectra were recorded from 200 to 260 nm at 25 °C. Temperature scans were recorded at a wavelength of 220 nm. Data were fitted to a 2-state unfolding model.

### Isothermal titration calorimetry analysis

The binding of β-(1,4), β-(1,3), and MLG-oligosaccharides to *Bp*SBP_MLG_ was measured at 25 °C in 10 mM sodium phosphate buffer (pH 7) using a MicroCal PEAQ-ITC (Malvern). The protein was extensively dialyzed against 10 mM sodium phosphate and all glycan ligands were also dissolved in the same buffer to minimize the heat of dilution. 13 injections of an oligosaccharide were titrated on *Bp*SBP_MLG_ present in the sample cell. Various preliminary titration setups were tried by changing amounts of protein (5–100 μM) and ligand (50 μM–10 mM) to get the best experimental conditions. Thermodynamic binding parameters [calculates binding affinity (K_d_), stoichiometry (n), enthalpy (ΔH), Gibbs free energy change (ΔG), and entropy (ΔS)] were determined using the MicroCal PEAQ-ITC analysis software.

### Bioinformatic analysis

The six protein sequences of a barley-β-glucan utilizing locus belonging to *B. producta* were downloaded from JGI/IMG/M in fasta format: *Bp*GH16_MLG_ (ID-2515951313), *Bp*GH94_MLG_ (ID-2515951314), AraC family (ID-2515951315), *Bp*SBP_MLG_ (ID-2515951316), ABC permease (ID-2515951317) and ABC ATP binding (ID-2515951318). To check the prevalence and identify homologs of these six proteins, we performed a BLAST sequence similarity search using NCBI BLAST (86). A detected homolog in different bacterial species was considered with the criteria of having at least 75% query coverage with at least 30% sequence similarity, and *Bp*GH16_MLG_ and *Bp*GH94_MLG_ should be present. Given that *Bp*GH16_MLG_ shared 33% sequence similarity with *Bo*GH16_MLG_ and both proteins were functional, these selection criteria were selected. All the output data was imported and filtered using in-house PERL and Shell scripts and sheets. The prevalence of hit distribution for all six proteins of a locus with similarity amongst all other species was plotted using a bubble plot in the R program using in-house scripts.

### Growth of Gram-positive bacteria using exogenous *Bp*GH16_MLG_

*R. faecis* JCM 31261, *B. pseudocatenulatum* JCM 1200^T^, *B. adolescentis* JCM 1275^T^, *B. coccoides* JCM 1395^T^, *B. bifidum* JCM 1254, and *A. caccae* JCM 13470^T^ were used for bacterial mono-culture experiments. All strains were maintained, and experiments were conducted under an anaerobic atmosphere consisting of 85% N_2_, 10% CO_2_, and 5% H_2_ at 37 °C. About 5 μl glycerol stock was initially inoculated to 5 ml of GAM for the primary culture of these strains. As soon as bacterial strains were grown, the cell pellet of each strain was separately obtained by centrifugation at 8000*g* for 1 min, and it was then washed three times with PBS. 5 μl of dissolved cell suspension of pellets in PBS was inoculated into a 200 μl minimal medium containing 1% barley β-glucan in presence or absence of exogenous supply of *Bp*GH16_MLG_ (0.05 mg/ml). The experiment was set up on 300 μl flat-bottomed 96-well microtiter plates. Reading was taken every interval of 8 h at 600 nm on a spectrophotometer.

## Data availability

Structure factors and atomic coordinates of *Bp*GH94_MLG_ have been deposited with the Protein Data Bank with accession code; PDB ID 8BOU. All other data are provided either in the Supporting information or can be available from the corresponding author upon reasonable request.

## Supporting information

This article contains supporting information (1, 3, 38, 39, 40, 48, 55, 83, 85, 87, 88, 89, 90, 91, 92, 93).

## Conflict of interest

The authors declare that they have no conflicts of interest with the contents of this article.
