# Supplementary material for: Utilization of dietary mixed-linkage β-glucans by the Firmicute Blautia producta
Source: J Biol Chem. 2023 May 11;299(6):104806. doi: 10.1016/j.jbc.2023.104806 (PMC10318527; doi:10.1016/j.jbc.2023.104806)
Supplement: Supporting information [file mmc1.docx]

**Utilization of dietary mixed-linkage *β-*glucans by the Firmicute *Blautia producta***

**Ravindra Pal Singh^1,2^*, Jayashree Niharika^1,2^, Raksha Thakur^2^, Ben A. Wagstaff^3^, Gulshan Kumar^2^, Rikuya Kurata^4^, Dhaval Patel^1^, Colin W. Levy^3^, Takatsugu Miyazaki^4,5^, Robert A Field^3^***

1. Gujarat Biotechnology University, Gujarat International Finance Tec (GIFT)-City, Gandhinagar- 382355, Gujarat, India
2. Division of Food and Nutritional Biotechnology, National Agri-Food Biotechnology Institute, SAS Nagar, Punjab, 140306, India
3. Department of Chemistry and Manchester Institute of Biotechnology, The University of Manchester, 131 Princess Street, Manchester M1 7DN, UK
4. Department of Agriculture, Graduate School of Integrated Science and Technology, Shizuoka University, 836 Ohya, Suruga-ku, Shizuoka City, Shizuoka, 422-8529, Japan
5. Research Institute of Green Science and Technology, Shizuoka University, 836 Ohya, Suruga-ku, Shizuoka City, Shizuoka, 422-8529, Japan

***Correspondence:** ravindra.singh@gbu.edu.in (R.P.S.); robert.field@manchester.ac.uk (R.A.F)

**Table of Contents**

|  | MALDI-TOF-MS Analysis …………………………………………………………... | 4 |
| --- | --- | --- |
|  | Proteomic analysis…………………………………………………............................. | 4 |
|  | Enzyme localization with immunofluorescence microscopy………………………… | 5 |
|  | Fluorophore assisted carbohydrate electrophoresis ………………………………….. | 5 |
|  | Crystallization trials with *Bp*SBP_MLG_ ………………………………………………… | 6 |
|  | Crystallography *Bp*GH94_MLG_………………………………………………………… | 6 |
|  | Deletion of solute binding protein by homologues recombination ………………….. | 7 |
|  | Qualitative analysis of short chain fatty acid ………………………………………… | 8 |
|  | Supplementary Table 1……………………………………………………………….. | 9 |
|  | Supplementary Table 2……………………………………………………………….. | 10 |
|  | Supplementary Table 3……………………………………………………………….. | 10 |
|  | Supplementary Table 4……………………………………………………………….. | 10 |
|  | Supplementary Figure 1. Phylogenetic tree of characterized GH16 sequences……………………………………………………………………………… | 11 |
|  | Supplementary Figure 2. Phylogenetic tree with functionally characterized GH94 phosphorylases………………………………………………………………………… | 12 |
|  | Supplementary Figure 3. Validation of polyclonal antibodies raised against BpGH94_MLG_ | 13 |
|  | Supplementary Figure 4. Effect of different buffering pH (A) and temperatures (B) on enzymatic activity of *Bp*GH16_MLG_…………………………………………………….. | 13 |
|  | Supplementary Figure 5. Screening of the *B_P_*GH16_MLG_ against a variety of different substrates for identification of linkage specificities. …..……………………………... | 14 |
|  | Supplementary Figure 6. Michaelis-Menten kinetics of *B_P_*GH16_MLG_ on different *β-* glucans…………………………………………………………………………………. | 15 |
|  | Supplementary Figure 7. Product limit digestibility of *Bp*GH16_MLG_…………………. | 16 |
|  | Supplementary Figure 8. Fluorophore-assisted carbohydrate electrophoresis and ^13^C (DEPT135) NMR spectra of purified limit digest products generated from barely-β-glucan…………………………………………………………………………………. | 17 |
|  | Supplementary Figure 9. Confidence of AlphaFold2 models used in this study……………………………………………………………………………………. | 18 |
|  | Supplementary Figure 10. Isothermal calorimetry analysis……………………………... | 19-20 |
|  | Supplementary Figure 11. Designing of plasmids for making *sbp* mutant in the *B. producta* ………………………………………………………………………………… | 21 |
|  | Supplementary Figure 12. Multiple sequence alignment of *Bp*SBP_MLG_ and its orthologs………………………………………………………………………………… | 22 |
|  | Supplementary Figure 13. Effect of the different pH and temperatures on the enzymatic activity of *B_P_*GH94_MLG_…………………………………………………………………... | 23 |
|  | Supplementary Figure 14. Activity and product limit digestibility of *Bp*GH94_MLG_……………………………………………………………………………… | 24 |
|  | Supplementary Figure 15. Michaelis-Menten kinetics of the *B_P_*GH94_MLG_……………………………………………………………………………….. | 25 |
|  | Supplementary Figure 16. Comparison of the active sites between *Bp*GH94_MLG_ and GH94 phosphorylases…………………………………………………………………………….. | 26 |
|  | Supplementary Figure 17. Effect of different temperatures and buffering pH on the enzymatic activity of *Bp*GH3-AR8_MLG_ and *Bp*GH3-X62_MLG_…………………………………………………………………………………….. | 27 |
|  | Supplementary Figure 18. Cytoplasmic digestion of oligosaccharides generated from barley-β*-*glucan and lichenan……………………………………………………………… | 28 |
|  | Supplementary Figure 19. Michaelis-Menten kinetics of the *Bp*GH3-AR8_MLG_ and *Bp*GH3-X62_MLG_…………………………………………………………………………………….. | 29 |
|  | Supplementary Figure 20. Time dependent degradation of various oligosaccharides by the *Bp*GH3-AR8_MLG_…………………………………………………………………………… | 30 |
|  | Supplementary Figure 21. Time dependent degradation of various oligosaccharides by the *Bp*GH3-X62_MLG_……………………………........................................................................ | 30 |
|  | Supplementary Figure 22. Qualitative analysis of short chain fatty acids…………………………………………………………………………………………. | 31 |
|  | Supplementary Figure 23. 10% SDS PAGE………………………………………………… | 32 |
|  | Supplementary Figure 24. 10% SDS PAGE and Native PAGE for *Bp*GH94_MLG_ | 32 |

1. **MALDI-TOF-MS Analysis**

Matrix assisted laser desorption/ionization- time-of-flight (MALDI-TOF) -mass spectra were recorded in positive ion mode for hydrolyzed products of various β glucans by the *Bp*GH16_MLG_ and *Bp*GH94_MLG_. The analysis was performed on an AB SCIEX 5800 mass spectrometer. For preparation of samples, 2 μl samples (10 mg/ml solution of enzymatic digestion) were mixed with 5 μl 2, 5-dihydroxybenzoic acid (dissolved in 30% acetonitrile) and dried on a target MALDI plate. A 5500 QTRAP mass spectrometer (AB Sciex, Foster City, CA, USA) was also employed to determine the molecular mass (MS) of generated oligosaccharides through the direct injection method using an electrospray ionization (ESI) source.

1. **Proteomic analysis**

The protein in the supernatant was precipitated by adding cold trichloroacetic acid (a final concentration of 20% TCA) and incubated at 1 h on ice. Afterward, precipitated protein was obtained by centrifugation (15,000 × g, 15 min at 4 °C) and washed three times with 500 μl ice-cold 0.01 M HCl in 90% acetone to remove a residual amount of TCA. Precipitated proteins were dissolved with 0.1 M Tris-HCl having 8 M urea (pH 8.5). Protein quantities were then calculated using Bradford assay. Approximately 25 μg protein was reduced with 2 µl of 25 mM dithiothreitol by incubating at 60°C for 30 min. Afterward, 1 μl of 50 mM iodoacetamide was added to the above reaction and incubated for 15 min in the dark at room temperature (RT). Consequently, trypsin digestion of protein was attained by incubating the reaction at 37°C for 16 h using 5 µg trypsin (MS grade, Promega). The next day, the reaction was quenched by adding one volume of 2% (w/v) trifluoroacetic acid, and the obtained peptides were used for mass spectrometry. Analysis of peptides was carried out as reported by [Singh et al. (83](#_ENREF_1)).

Using the manufacturer's protocol, peptides from each replicate of B. producta ATCC 27340 were initially cleaned-up using Oasis HLB 1 cc Vac cartridges (Waters). The SWATH-MS analysis of each sample was performed on a quadrupole-TOF hybrid mass spectrometer (TripleTOF 6600, SCIEX) coupled to an Eksigent Nano LC-425 system. A SWATH-MS method was created using 93 precursor isolation windows which were defined on the basis of precursor m/z frequencies in DDA run using the SWATH Variable Window Calculator (SCIEX), with a limitation window of 5 m/z. Analysis parameters were optimized before any sample was run. Nebulizer gas and curtain gas were maintained at 20 and 25 psi, respectively. The ion spray voltage and temperature were set to 5.5 kV and 250 °C, respectively. For desalting, about 4 μg peptides were loaded on a trap-column (ChromXP C18 CL 5 µm 120 Å, Eksigent, SCIEX), and purification was performed with a flow rate of 10 µl / min for 10 min. Peptides were fractionated on a reverse-phase C18 analytical column (ChromXP C18, 3 µm 120 Å, Eksigent, SCIEX) in a 57 min gradient run with a flow rate of 5 µl/min using water (buffer A) and acetonitrile (buffer B) with 0.1% formic acid as follows: 0-3 min 3% B; 3-38 min linear increase B to 25%; 38-43 min increase B to 32%; 43-45 min increase B to 80%; followed by a ramp to 90% B and re-equilibration to 3% B.

The sample was analyzed in the data-independent acquisition (DIA) mode, which was acquired using Analyst TF 1.7.1 Software (SCIEX). Accumulation time was set to 0.25 s for the MS scan (400–1250 m/z) and 0.025 s for the MS/MS scans (100–1500 m/z). Rolling collision energies were determined for each window on the basis of the m/z range of each SWATH and a charge 2^+^ ion with a collision energy (CE) spread of 5. Total cycle time was 2.6 sec. The SWATH-MS run files were analyzed in Spectronaut^TM^ 15.4 software (Biognosys) using directDIATM library-free workflow. A Pulsar search engine was used for protein identification, enzyme cleavage rule was set to trypsin and carbamidomethylation was set as fixed modification. Protein N-terminal acetylation and oxidation were determined as variable modifications. A protein FASTA file for B. producta from UniProtKB (UP000464715, 5365 protein entries) was used, and protein identification was performed with 0.05 FDR. The Mutated decoy method was used for FDR analysis. The Direct DIATM analysis was executed with default settings, and quantitation was done using the area at the MS2 level. Cross-run data normalization was carried out in which the normalization strategy was set to automatic. Quantitative data was exported as a ‘Run Pivot Report’ and analysis of differential protein expression was performed in Microsoft Excel Sheet.

1. **Enzyme localization with immunofluorescence microscopy**

The cells were grown until they reached mid-exponential phase (A600, about- 0.6) in minimal media containing 1 % w/v barley β*-*glucan as the sole carbon source. Cells were then pelleted by centrifugation and washed 3 times with PBS. Afterward, the cells were fixed by incubating in 4.5 % formaldehyde in PBS for 2 h at RT, washed three times with PBS, and blocked for 1 day at 4 °C in a blocking solution. The blocking solution consisted of 3 % bovine serum albumin and 0.02% NaN3 in PBS. The cells were then incubated separately with polyclonal antibodies raised against BpGH16_MLG_ and BpGH94_MLG_ (GenScript Biotech, Singapore, PTE.LTD) for 3 h at RT (1:100 dilution of the antibody in blocking solution). For secondary labelling, primary polyclonal antibodies labelled cells were pelleted, washed 3 times in 1 mL of PBS, and then resuspended in 0.4 mL goat anti-rabbit IgG Alexa-Fluor 488 (diluted 1:1000 in blocking solution). Suspended cells were incubated 1 h at room temperature in dark condition for proper labelling. The cells were again washed three times and resuspended in 50 µ of PBS containing ProLong Gold Antifade which prevents bleaching of Alexa-Fluor 488 fluorescence. These cells were then mounted on prepared agarose pads on glass slides and capped with coverslips. Imaging was done using a confocal laser scanning microscope (Carl Zeiss LSM880) at 100 × magnification. All chemicals were purchased from Thermo Fisher Scientific.

1. **Fluorophore assisted carbohydrate electrophoresis**

About 50 - 100 µg of all standards and limit-digest products of *Bp*GH16_MLG_ and *Bp*GH94_MLG_ were freeze-dried for FACE. A volume of 3 µl of 0.15 M 7-Amino-1,3-naphthalenedisulfonic acid monopotassium salt monohydrate (ANDS, a solution of acetic acid and water at the ratio of 3:17) or 3 µl of 0.15 M 8-aminopyrene-1,3,6-trisulfonic acid was added to the dried enzymatic reaction, and it was then incubated for 1 h. Following 5 µl freshly made 1 M sodium cyanoborohydride in DMSO was added to every sample and further incubated overnight at 37 ºC in dark condition. Samples were ventilated in a fume hood for 30 min to remove remaining HCN gas before freeze drying, and it was then re-suspended in 20% glycerol. 2 to 10 μL of different samples (standard and limit-digest products) were loaded onto a 37% polyacrylamide gel and migrated at 100 V for 3 h in the dark at 4 °C. The gels were visualized under UV transilluminator.

1. **Crystallization trials with *Bp*SBP_MLG_**

For crystallization trials of *Bp*SBP_MLG_, the purified proteins (*Bp*SBP_MLG_ and *Bp*SBP_ΔN_) were treated with thrombin to remove N-terminal His tag in PBS. Subsequently, the proteins were further purified by anion-exchange chromatography with Mono Q 5/50 GL column (Cytiva, Tokyo, Japan)- pre-equilibrated with 20 mM sodium phosphate buffer (pH 7.0). It was then eluted with a linear gradient of 0–0.5 M NaCl in the same buffer. Fractions containing the recombinant protein were concentrated using Vivaspin 20 centrifugal concentrator (10,000 molecular weight cut-off). It was then again purified by gel filtration chromatography with Superdex 200 increase 10/300 column (Cytiva) equilibrated with 20 mM sodium phosphate buffer (pH 7.0) containing 300 mM NaCl. In some cases, lysine alkylation was introduced using a Reductive Alkylation Kit (Hampton Research, Aliso Viejo, CA, USA) as per the manufacturer’s protocol to facilitate protein crystallization. Before crystallization, purified proteins were concentrated to 20–120 mg/mL using Vivaspin 20 in 10 mM HEPES-NaOH (pH 7.0) buffer. Initial crystallization screening was performed at 4 ºC or 20 ºC using the sitting-drop or hanging-drop vapor diffusion methods. One microliter of protein was mixed with an equal volume of crystallization solutions included in Crystal Screen, Crystal Screen 2, PEG/Ion Screen, and PEG/Ion 2 Screen kits (Hampton Research). Cocrystallization with ligands was also tried by adding 1–10 mM cellotriose, laminaritriose, and G4G3G into the protein solution prior to setting up. Modelling with *Bp*GH16_MLG_ and *Bp*SBP_MLG_ were performed using AlphaFold2/ColabFold (39, 40). Confidence of AlphaFold2 models were predicted with predicted local distance difference test (pLDDT) score which is reported in the figure S9.

For crystallization screening of *Bp*SBP (WP_018595366.1), the expression plasmid for N-terminally truncated protein (*Bp*SBP_ΔN_, 1–32 residues were deleted) was constructed by PCR using KOD one™ DNA polymerase (Toyobo, Osaka, Japan), *Bp*SBP-pET28a(+) plasmid as a template, and primer pairs, *Bp*SBP_ΔN__F and *Bp*SBP_ΔN__R (Table S3). The PCR solution was treated with DpnI and then added to *E. coli* DH5α competent cells, followed by positive clones were selected. The extracted plasmid was sequenced to confirm the deletion.

1. **Crystallography *Bp*GH94_MLG_**

Crystals of *Bp*GH94_MLG_ were prepared by mixing 200 nl of 14 mg mL^−1^ protein in tris-buffered saline, pH 7.5 with equal volumes of precipitant. All trials were conducted by sitting-drop vapour diffusion and incubated at 4 °C. Crystals were formed in 0.1 M carboxylic acids, 0.1 M Imidazole MES buffer pH 6.5, 60% precipitant mix 3 (40% v/v Glycerol, 20% w/v PEG 4000) [Morpheus G3, Molecular Dimensions]. Individual *Bp*GH94_MLG_ crystals were cryoprotected in mother liquor prior to flash cooling in liquid nitrogen. Data were collected from single crystals at Diamond Light Source, and afterward scaled and reduced with Xia2. Preliminary phasing was performed by molecular replacement in Phaser using a search model derived from *Cellvibrio gilvus* Cellobiose Phosphorylase (PDB code: 2CQT). Iterative cycles of rebuilding and refinement were performed in COOT and Phenix.refine, respectively. Structure validation with PDBREDO and MolProbity were integrated into the iterative rebuild and refinement process. Complete data refinement statistics and collection can be found in the Supplementary Table 1. Coordinates and structure factors have been deposited in the Protein Data Bank under accession code 8BOU.

1. **Deletion of solute binding protein by homologues recombination**

Following an established procedure, we employed a markerless gene deletion strategy for developing *B. producta*ΔSBP cells ([4](#_ENREF_4)8). A conditional replication plasmid (pBS423-Δ*rep*A, having spectinomycin-resistant gene) and a replicated plasmid (pTBR101-CM, having chloramphenicol-resistance gene) were purchased from RIKEN BioResource Research Center, Japan. The pBS423-Δ*rep*A was further modified by replacing the spectinomycin-resistant gene with the ampicillin-resistant gene.

The pBS423-Δ*rep*A- Δ*sbp* was constructed by inserting 905 bp 5' and 1151 bp 3' flanking regions but lacking 1.2 kb core region of *Bp*SBP_MLG_. It was inserted between Pst1 sites of the vector. GenScript Biotech (Singapore) PTE LTD carried out synthesis of the 2.2 kb flanking region and insertion into pBS423-Δ*rep*A. Competent cells of *B. producta* were prepared after inoculating 1 % primary culture into 300 ml GAM containing glucose (0.2%), sucrose (0.5 M), and glycine (1%). It was incubated until A600 reached 0.5-0.7 at 37 °C under anaerobic conditions. Afterward, cultures were transferred into 50 ml centrifuge tubes and centrifuged at 3000 × g for 20 min. The supernatant was discarded, and pellets were washed three times with washing solution (0.5 M sucrose and 10% glycerol) and stored at -80°C in aliquots of 50 µl.

The first cross-over event for knocking out was achieved by integrating 0.2 to 1 µg pBS423-Δ*rep*A- Δ*sbp* in 50 µl competent cells of *B. producta* through electroporation using a condition of 25 µF, 2.0 kV, and 200 Ω. Gene Pulser Xcell (Bio-Rad Laboratories, Hercules, CA, USA) was used for this purpose. The treated cells were instantly mixed with 1 ml anaerobically pre-incubated GAM medium containing 0.2 % glucose, 0.5 M sucrose, 1 % glycine, 20 mM MgCl_2_.6H2O, and 2 mM CaCl_2_. It was then anaerobically incubated for 3 h and the cells were spreaded onto GAM agar plate containing 100 µg/ml ampicillin, 0.7 % glucose, 20 % sucrose, 2.5 mM MgCl_2_.6H2O, and 2.5 mM CaCl_2_. The plates were anaerobically incubated at 37 °C until colonies were visible (typically 40 to 72 hours).

After confirmation of the first crossover integration of B. producta - pBS423-ΔrepA- Δsbp by PCR, the second crossover was performed by the pTBR101-CM to excise the integrated pBS423-ΔrepA. During the second crossover integration of the pTBR101-CM, pBS423-ΔrepA was eliminated from transformed cells due to plasmid incompatibility during sub-culturing on GAM agar plate containing 100 µg/ml chloramphenicol. The original sbp (mutant allele) elimination from the genome was confirmed by PCR and sequencing using a set of primers. Once mutant cells were confirmed, growth patterns of native and mutant cells were performed on 1% barley-β-glucan.

1. **Qualitative analysis of short chain fatty acid (SCFAs)**

*B. producta* ATCC 27340 was cultivated in a minimal medium containing 1 % barley-β-glucan until cell density at A600 reached 0.6. Thereafter, cells were removed by centrifugation at 15000 × g for 10 min, and supernatant was collected. The supernatant was diluted 50 % with Milli-Q water (Merck Millipore, 0.22 µm filtered, resistivity 18.1–18.3 MΩ cm) and adjusted pH 2 with formic acid. The supernatant was then filtered using 0.2 µm nylon syringe filters, and about 20 µl fraction was loaded into the Hi-Plex H column (300×7.7 mm; 8 µm particle size, Agilent Tech). Different SCFAs were separated using 0.1% formic acid in Milli-Q water as mobile phase using POSTNOVA size exclusion chromatography system which was connected with SPD-20A (prominence UV/VIS detector). An isocratic flow rate of 0.6 ml/min at 50 °C for 90 min was used for separating different SCFAs. A standard of volatile Acids Mix (Cayman Chemicals Co., Michigan, United States) was used at a concentration range 100-6400 µM for determining retention time and detection of each SCFA.

**Supplementary Table 1:** **Data collection and refinement statistics for *Bp*GH94_MLG_**

| **Data collection** | ***Bp*GH94_MLG_** |
| --- | --- |
| Space group | P 21 21 21 |
| Cell dimensions |  |
| *a*, *b*, *c* (Å) | 98.46, 135.29, 166.99 |
| α, β, γ (°) | 90, 90, 90 |
| Resolution (Å) | 48.46  - 2.32 (2.403  - 2.32) |
| Rmerge | 0.151 (1.699) |
| I / σI | 10.92 (0.80) |
| Completeness (%) | 99.95 (99.92) |
| Multiplicity | 11.7 (12.7) |
| CC1/2 | 0.998 (0.602) |
| CC* | 0.999 (0.867) |
|  |  |
| **Refinement** |  |
| Resolution (Å) |  |
| No. reflections | 96989 (9555) |
| Reflections used for R-free | 4886 (488) |
| Rwork / Rfree | 0.1779 (0.3088)/ 0.2224 (0.3490) |
| No. atoms | 13781 |
| Protein | 13014 |
| Ligand/ion | 62 |
| Solvent | 705 |
| B-factors | 63.41 |
| Protein | 63.43 |
| Ligand/ion | 75.09 |
| Solvent | 61.92 |
| R.m.s. deviations |  |
| Bond lengths (Å) | 0.002 |
| Bond angles (°) | 0.55 |

*Values in parentheses are for the highest-resolution shell.

**Supplementary Table 2:** Primers for quantitative reverse transcription PCR (RT-qPCR) to check gene expression of β-glucan utilization locus in *B. producta*.

| **Primers** | **Sequence 5’ to 3’** |
| --- | --- |
| Blautia-5363-F | AGCATATCCGGCACCTTATG |
| Blautia-5363-R | GTCCGGTTCTCTCAAATCCA |
| Blautia-5364-F | CCGGATGATGCTTCCTATG |
| Blautia-5364-R | GTTGCTTTTCCACGGTTTG |
| Blautia-5365-F | CCGCTCCATCATTGAAGAA |
| Blautia-5365-R | AACGATATGCCTGCTCTGC |
| Blautia-5366-F | ATGGGCTGTTACGACTCCTC |
| Blautia-5366-R | CCAGGAAGTCAGACGCAAAG |
| Blautia-5367-F | ACGAATGGTCAGGGAAGTCC |
| Blautia-5367-R | TCCTCCTCAGTTTGTGTGC |
| Blautia-5368-F | TGGCCATTCTTGTTATGCCG |
| Blautia-5368-R | TGACAACGGAAGAGATGCCT |

**Supplementary Table 3:** A list of primers used for cloning of enzymes and solute binding protein of *B. producta*.

| **Primers** | **Sequence 5’ to 3’** |
| --- | --- |
| *Bp*SBP_F | ACAGCAAATGGGTCGCGCTAGCGGCGCATCTGATTCCCAAAATACAGC |
| *Bp*SBP_R | GGTGGTGGTGGTGGTGCTCGAGTTATTCCGGCCAATGAATCTCCGT |
| *Bp*GH94-2-F | ACAGCAAATGGGTCGCGGATCCATGTGTGCGGTTTTGAACCATACC |
| *Bp*GH94-2-R | GTGGTGGTGGTGGTGCTCGAGTTATCCCATAACTACTCTCACTGTATG |
| β-Glc-AR8 F | CTAGTGGCTAGCGTGGAGAATAAATATGTGATC |
| β-Glc-AR8 R | CTAGTCTCGAGCTAAAATCCTCTGTCCATCTT |
| β-Glc-X62 F | CTTCGCTAGCACATTTACAGGAACTACTTC |
| β-Glc-X62 R | TCACCTCGAGCTAGTTTGATCTTTCTACACT |
| *Bp*SBPΔN_F | CATATGGCTAGCGGCGAAGAAGGAAAAATTATTA |
| *Bp*SBPΔN_R | TAATTTTTCCTTCTTCGCCGCTAGCCATATGGCTGC |

**Supplementary Table 4:** A list of primers used for site-directed mutagenesis experiments on the *Bp*GH16.

| **Primers** | **Sequence 5’ to 3’** |
| --- | --- |
| R331A_F | CACCAGCGGTGCAATCAACACCCAGAAG |
| R331A_R | TAGCTAACATCGCCGCTCTCGTCCACTTTCTT |
| L358A_F | TAAAGGCTTCGCACCGGCGTTTTGG |
| L358A_R | CCCTCCGGCACCTTAATTTTCGCCTC |
| W362A_F | GCCGGCGTTTGCAATGATGCCGA |
| W362A_R | AGGAAGCCTTTACCCTCCGGCACCTTAATTTTC |
| E379A_F | AGTGCGGCGCAATCGATATTATGGAA |
| E379A_R | TCGGCCACTGGCCATACAGG |
| D381A_F | GGCGAGATCGCAATTATGGAAGTTCTGG |
| D381A_R | GCACTTCGGCCACTGGCCA |
| E384A_F | GATATTATGGCAGTTCTGGGCGACAAAAC |
| E384A_R | GATCTCGCCGCACTTCGGC |
| H398A_F | CGGCACCCTGGCATTCGGTGAACCG |
| H398A_R | TGCGCGGTATCGGTTTTG |


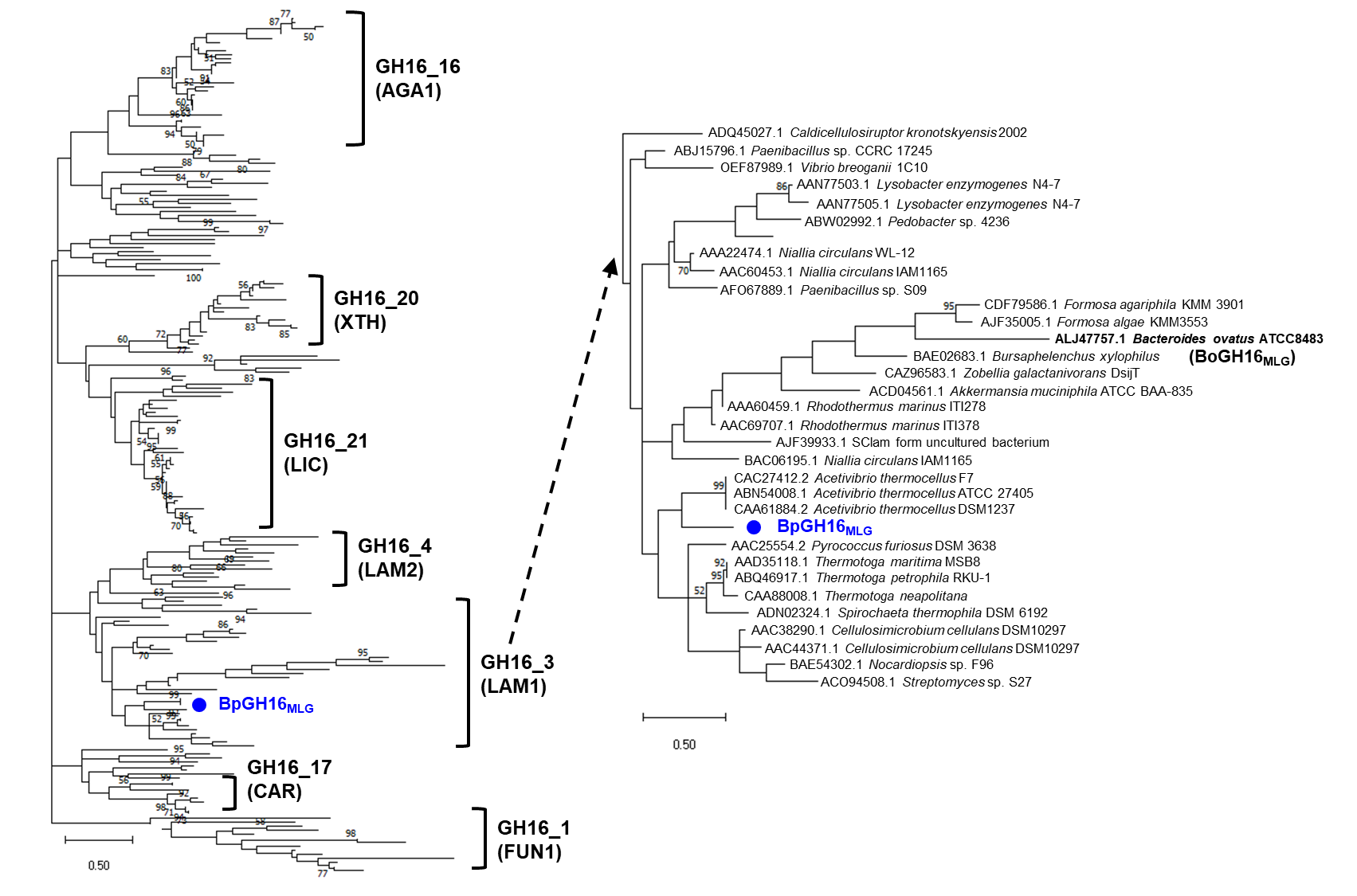


**Supplementary Figure 1. Phylogenetic tree of characterized GH16 sequences.** The 212 characterized sequences listed in the CAZy database were obtained from the NCBI Protein database. The sequence alignment was carried out using MUSCLE and the tree (left) was generated with the Maximum Likelihood method conducted by MEGA X ([87](#_ENREF_5)). Bootstrap values based on 1,000 replicates are shown. Some subfamilies in GH16 are indicated with their mnemonics in parenthesis according to Viborg et al. ([38](#_ENREF_6)). The phylogenetic tree of GH16_3 (right) is depicted with each accession number available at the NCBI Protein database.


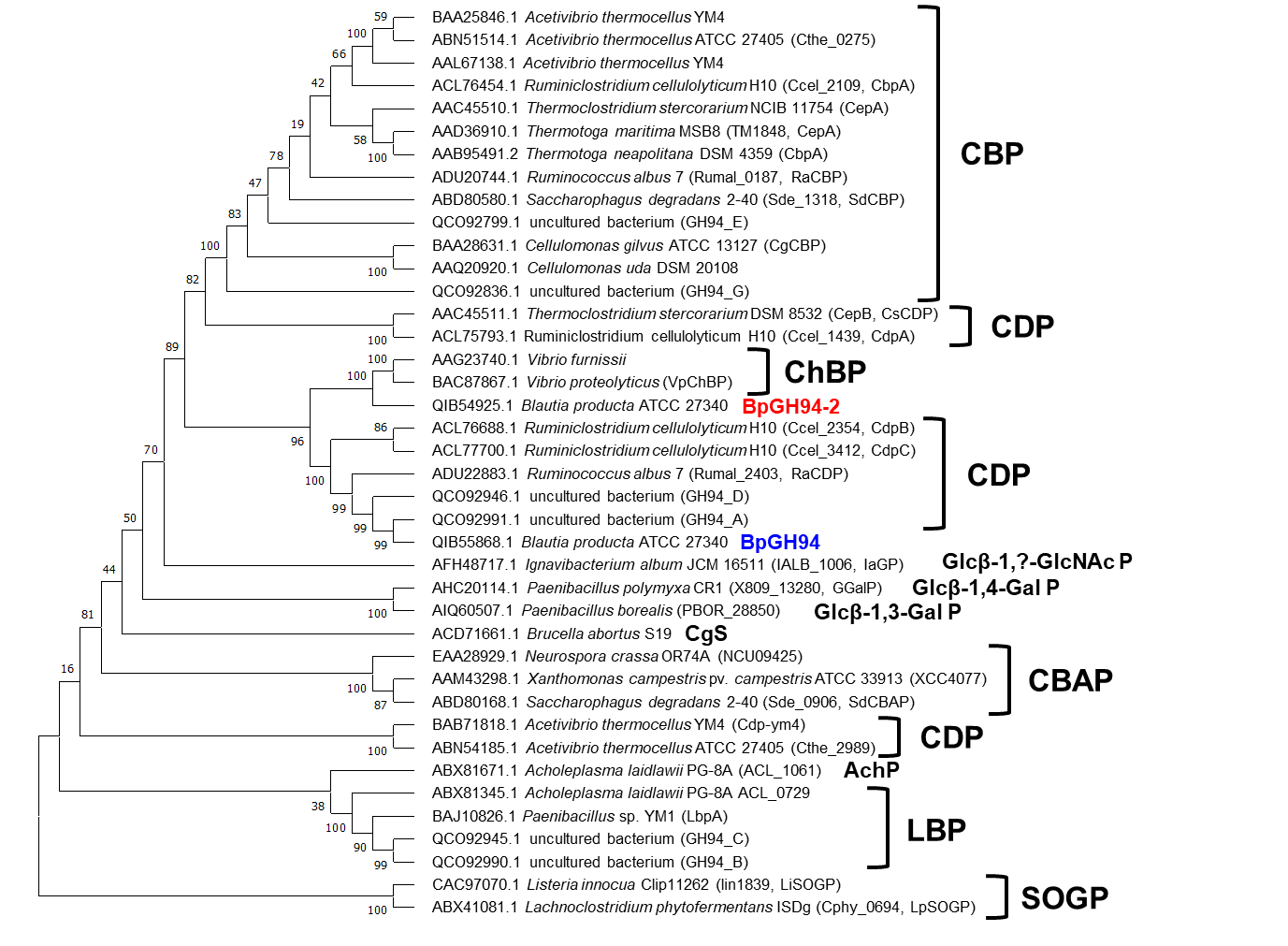


**Supplementary Figure 2.** **Phylogenetic tree with functionally characterized GH94 phosphorylases.** The sequences were obtained from the NCBI Protein database and their accession codes have been described in the phylogenetic tree. Locus tags and protein names have been also described in parentheses. The sequence alignment was performed using the MUSCLE and the tree was generated with the Maximum Likelihood method conducted by MEGA X. Bootstrap values based on 1,000 replicates are shown. Abbreviations used: CBP, cellobiose phosphorylase; CDP, cellodextrin phosphorylase; ChBP, N,N’-diacetylchitobiose phosphorylase; Glcβ-1,?-GlcNAcP, phosphorylase that showed reverse phosphorolytic reaction with α-glucose 1-phosphate donor and GlcNAc acceptor; Glcβ-(1,4)-Gal P, 4-O-β-glucosyl-galactose phosphorylase; Glcβ-(1,3)-Gal P, 3-O-β-glucosyl-galactose (solabiose) phosphorylase; CgS, cyclic β-(1,2)-glucan synthetase; CBAP, cellobionic acid phosphorylase; AchP, acholetin (poly-β-1,3-GlcNAc) phosphorylase; LBP, laminaribiose phosphorylase; SOGP, (1,2)-β-oligoglucan phosphorylase.

B


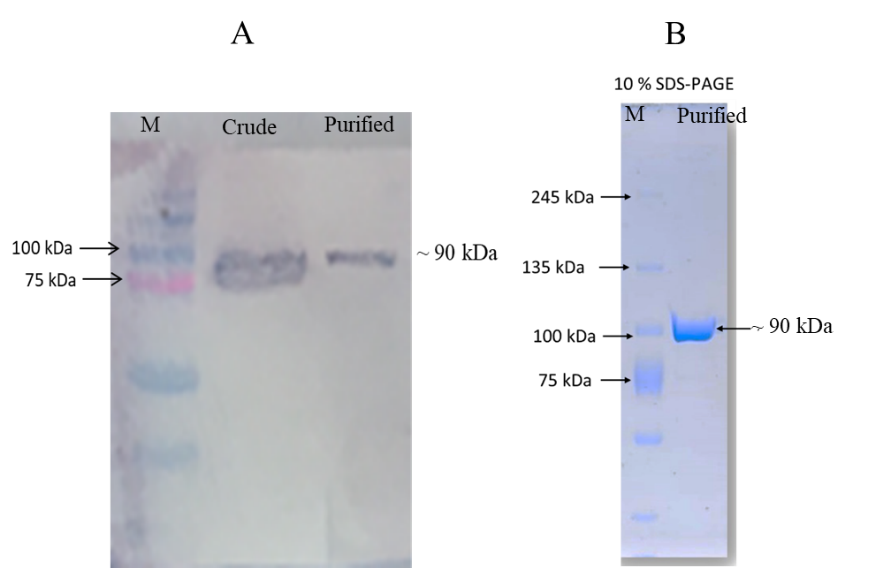


**Supplementary Figure 3. Validation of customized polyclonal antibody for BpGH94_MLG_**. (A) Recombinant BpGH94_MLG_ was first transferred to polyvinylidene difluoride (PVDF) membrane from 10% SDS-PAGE and was then incubated with polyclonal antibodies raised against BpGH94_MLG_ (1:100 dilution of the antibody in blocking solution). After washing blot three times in 1 mL of PBS, it was labelled with secondary goat anti-rabbit IgG HRP. (B) Purified recombinant BpGH94_MLG_ was also run on 10% SDS-PAGE. Panel B of this Fig. S3 is reused in this Fig. S24 as penal A for providing an immediate reference to dimerization nature of a recombinant *Bp*GH94_MLG_.


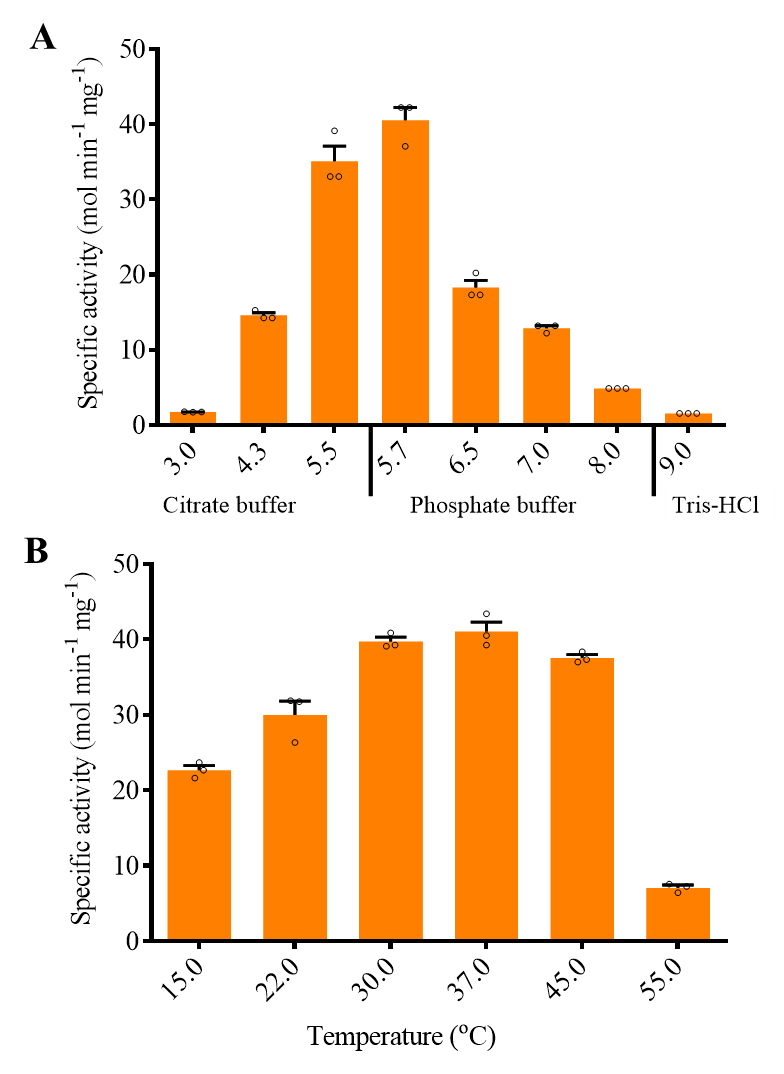


**Supplementary Figure 4.** Effect of different buffering pH (A) and temperatures (B) on enzymatic activity of *Bp*GH16_MLG_. *p*NP-*β*-laminaribioside was used as a substrate at a final concentration of 2 mM, and specific activity was calculated on the released amount of para-nitrophenol.

**
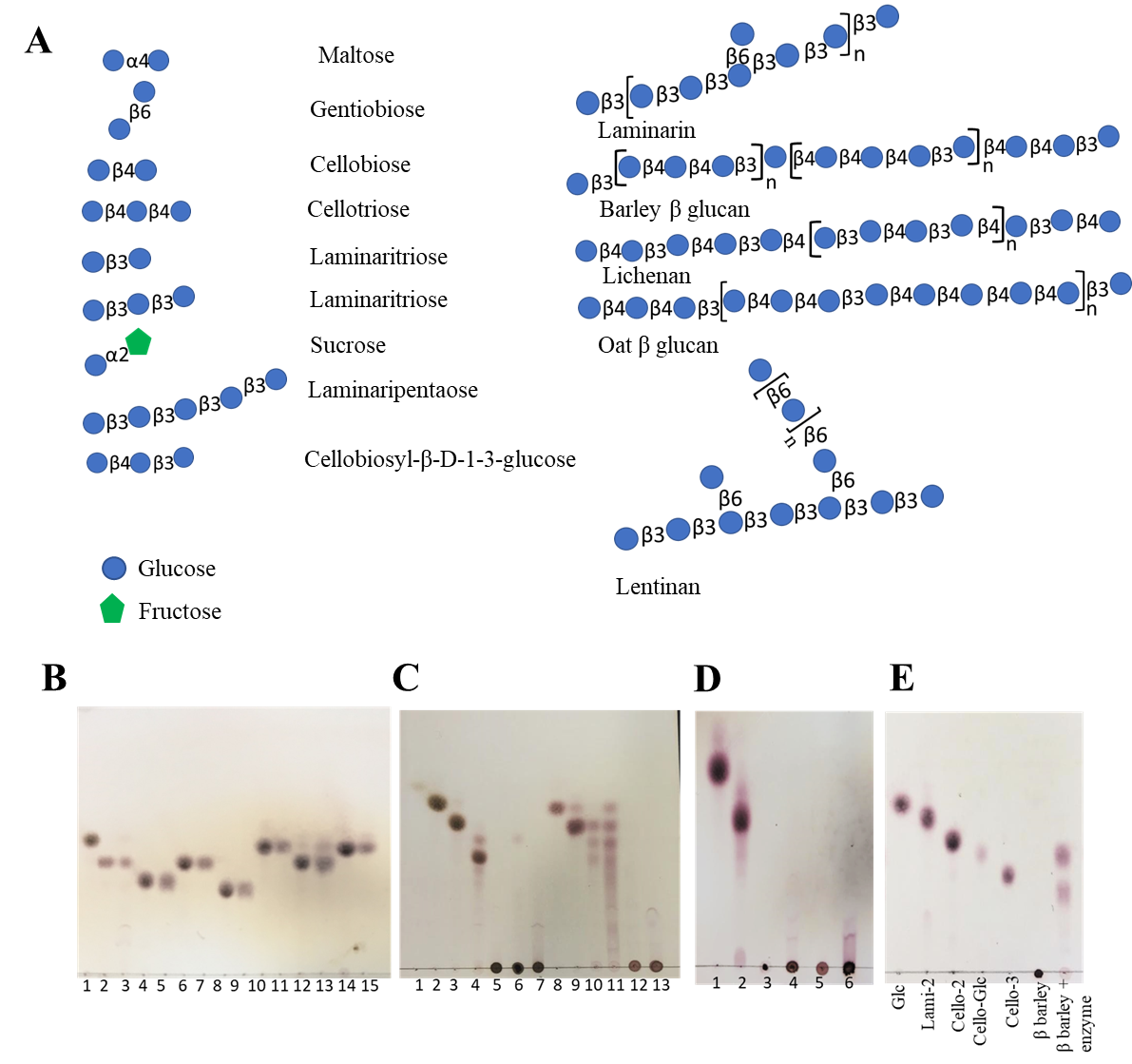
**

**Supplementary Figure 5. Screening of the *B_P_*GH16_MLG_ against a variety of different substrates for identification of linkage specificity.** (**A**) Representative structures of oligosaccharides and polysaccharides as per symbolic nomenclature for glycans (https://www.ncbi.nlm.nih.gov/glycans/snfg.html). Representative structure of laminarin ([1](#_ENREF_7)), barley- and oat β -glucan ([3](#_ENREF_8)), lichenan (88) and lentinan (89) were drawn on the basis of previously published articles.

**(B)** (1) Glc, (2) maltose, (3) maltose + *B_P_*GH16, (4) gentiobiose, (5) gentiobiose + *B_P_*GH16, (6) cellobiose, (7) cellobiose + *B_P_*GH16, (8) cellotriose, (9) cellotriose + *B_P_*GH16, (10) laminaribiose, (11) Laminaribiose + *B_P_*GH16, (12) laminaritriose, (13) laminaritriose + *B_P_*GH16, (14) sucrose and (15) sucrose + *B_P_*GH16.

**(C)** (1) Glc, (2) laminaribiose, (3) laminaritriose, (4) laminaripentaose, (5) laminarin, (6) lichenan, (7) lentinan, (8) laminaribiose + *B_P_*GH16, (9) laminaritriose + *B_P_*GH16, (10) laminaripentaose + *B_P_*GH16, (11) laminarin + *B_P_*GH16, (12) lichenan + *B_P_*GH16 and (13) lentinan + *B_P_*GH16. Lane 7 – control of lentinan revealed some impurities; therefore, it was dialysed against 10 kDa filter membrane and then used in reaction as shown the TLC-C. Spots of digested lichenan, in lane 12, were not properly visualized due to low amount of reaction mixture was loaded. 1 µl of each sample was loaded on the TLC.

**(D)** (1) Laminaribiose, (2) laminaripentaose, (3) oat β-glucan control, (4) oat β-glucan + *B_P_*GH16, (5) lentinan and (6) lentinan + *B_P_*GH16.

**(E)** (1) Glc, (2) laminaribiose, (3) cellobiose, (4) G4G3G, (5) cellotriose, (6) barley-β-glucan and (7) barley-β-glucan + *B_P_*GH16.


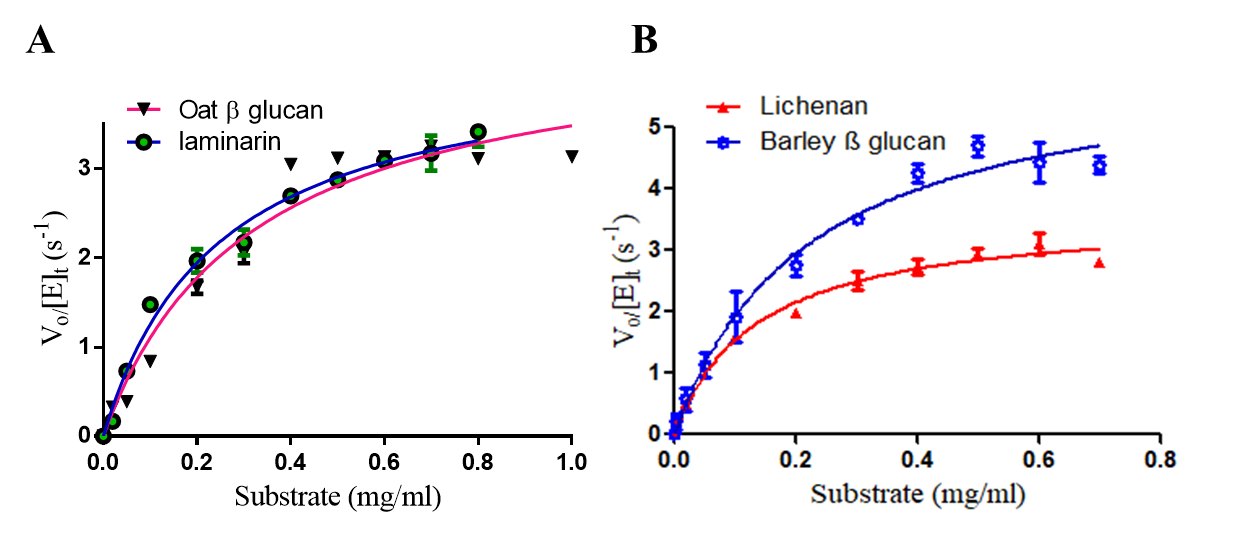


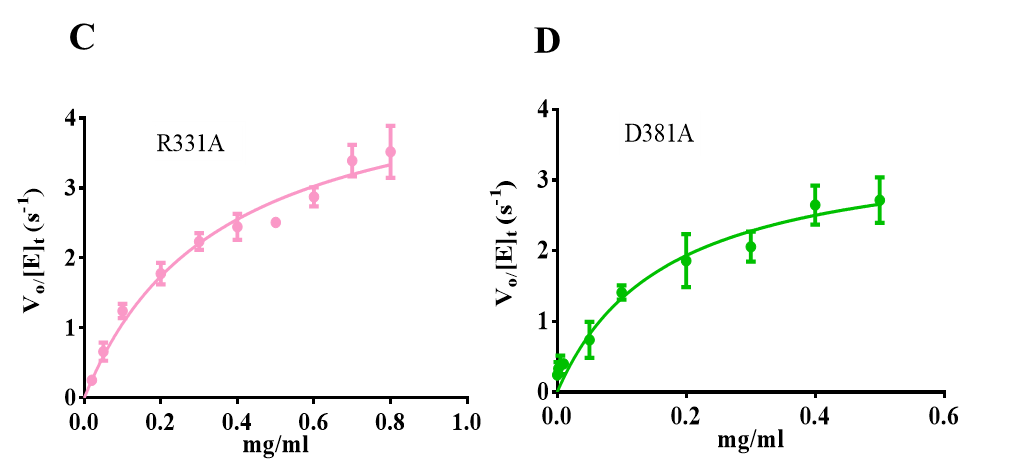


**Supplementary Figure 6.** **Michaelis-Menten kinetics of *B_P_*GH16_MLG_ on different *β-* glucans.** (A) Michaelis-Menten plot demonstrates the enzyme kinetics of oat β glucan and laminarin. (B) Michaelis-Menten plot shows the enzyme kinetics of lichenan and barley-β-glucan. (C and D) Michaelis-Menten plot shows the kinetic parameters on barley-β-glucan with mutant of *Bp*GH16_MLG_. Enzymatic assays were determined with 3,5- dinitrosalicylic acid (DNS) assay (85). It was performed with three biological replicates.


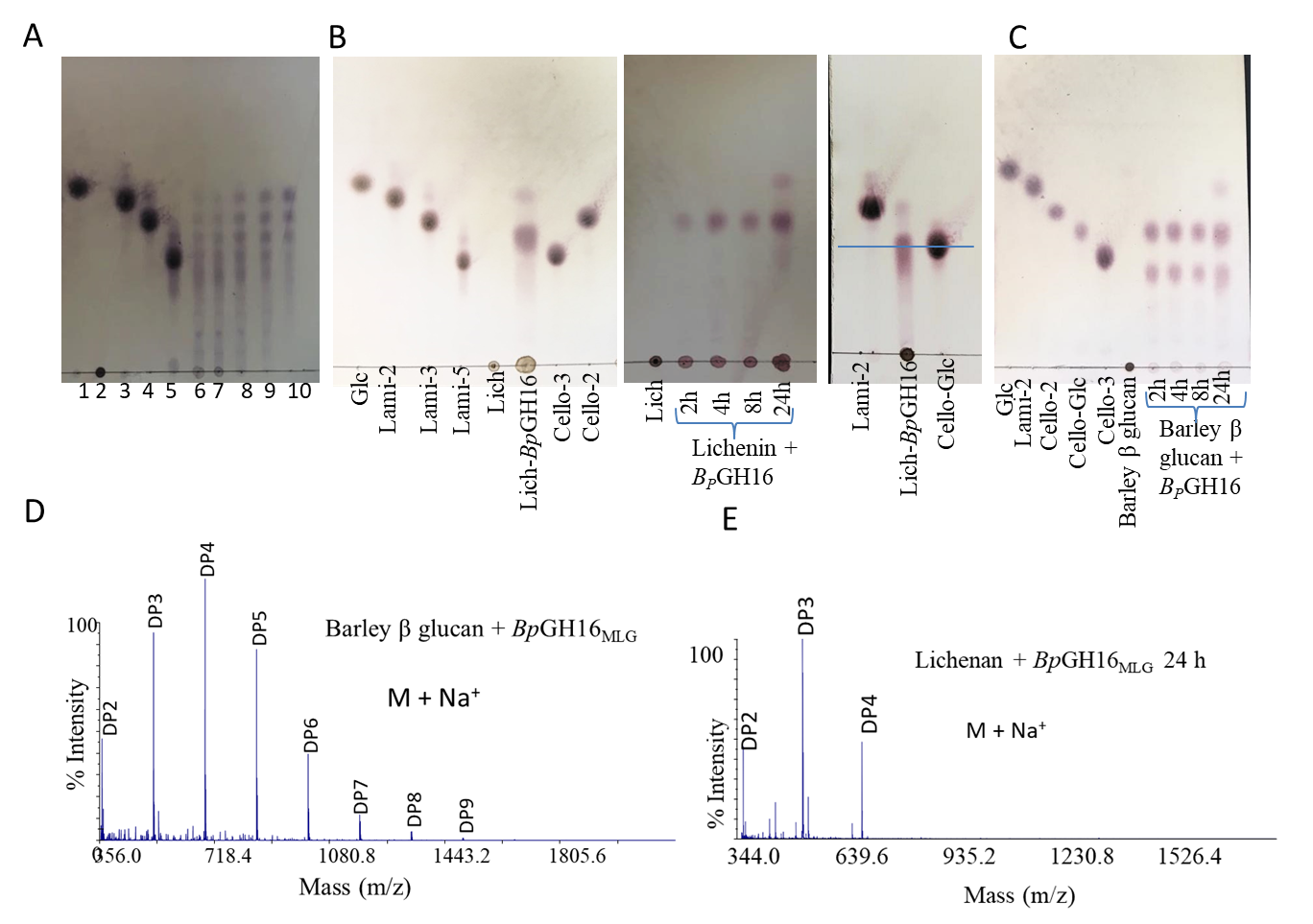


**Supplementary Figure 7. Product limit digestibility of *Bp*GH16_MLG_.** Limit digest production of (A) laminarin and laminaripentaose, (B) lichenan (Lich), and (C) barley-β-glucan with *Bp*GH16_MLG_ after incubating at 37 °C. In panel A (1) glucose (Glc), (2) laminarin, (3) laminaribiose (lami-2) (4) laminaritriose (lami-3), (5) laminaripentaose (lami-5), (6) laminarin digested for 1h, (7) laminarin digested for 2h, (8) laminarin digested for 4h, (9) laminarin digested for 8h, and (10) laminarin digested for 24h. Cello2/3; - cellobiose/cellotriose, and cello-Glc; - G4G3G. (D and E) MALDI-MS analysis shows hydrolysis products of barley-β-glucan and lichenan by *Bp*GH16_MLG_. TLC analysis suggested that there were initially considerable various length oligosaccharides generated from every substrate, which were gradually converted into shorter-chain oligosaccharides during the course of 24 h incubating with *Bp*GH16_MLG_.


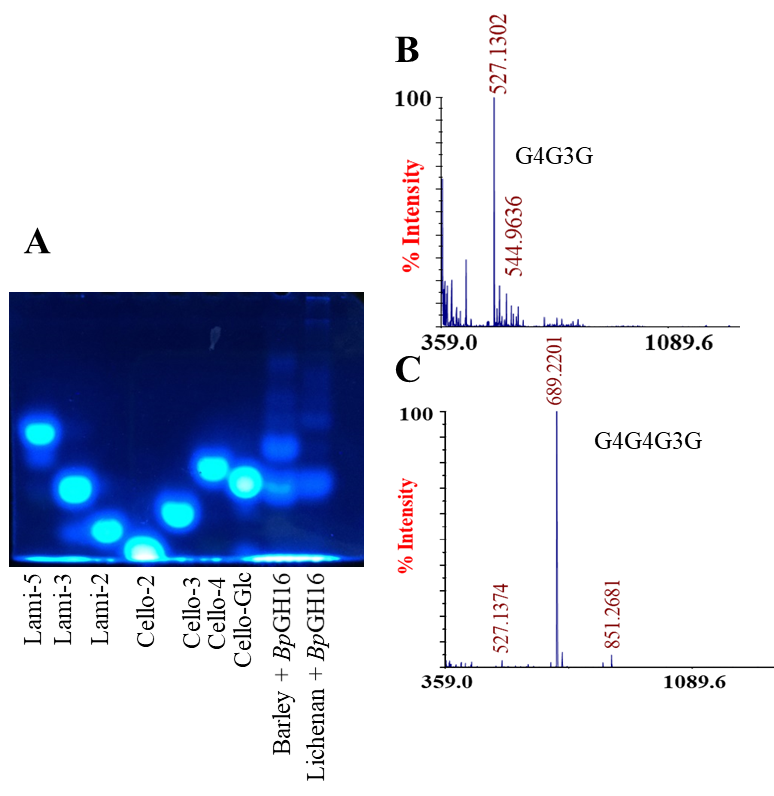


D


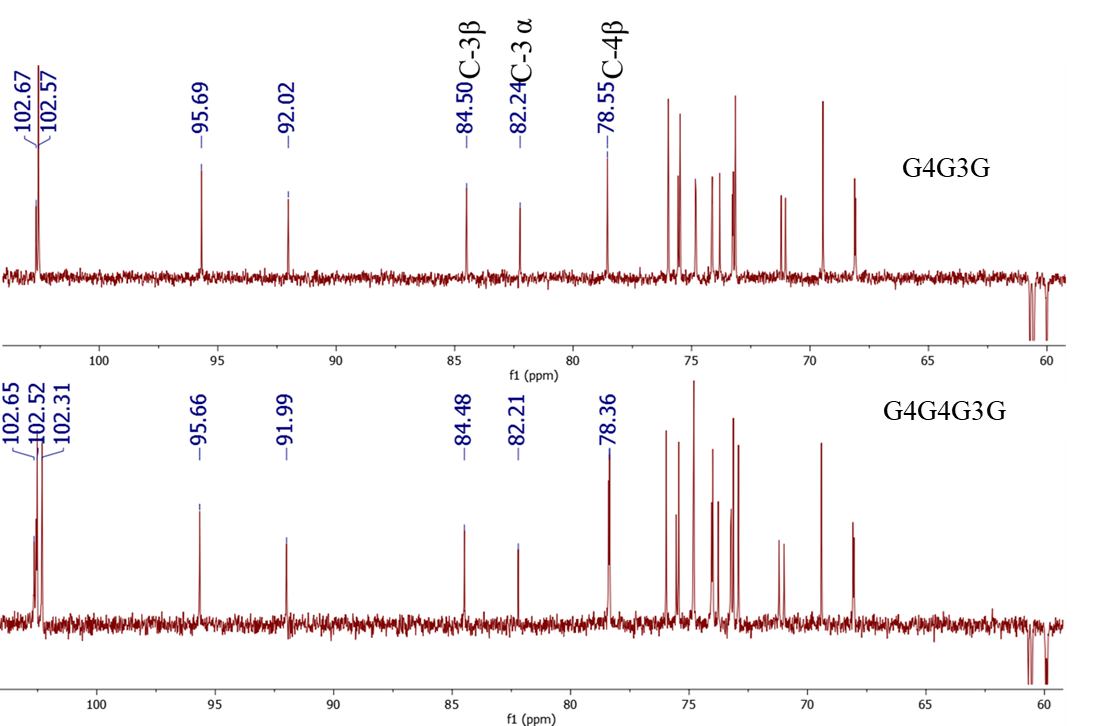


**Supplementary Figure 8.** **Fluorophore-assisted carbohydrate electrophoresis and ^13^C (DEPT135) NMR spectra of purified limit digest products generated from barely-β-glucan.** (A) Oligosaccharides obtained from the *Bp*GH16_MLG_ hydrolyzed barley-β-glucan and lichenan were fluorescently tagged and run onto a 37% polyacrylamide gel electrophoresis. Purification of oligosaccharides generated from barley-β-glucan was achieved by Toyopearl HW-40 resin and HPLC using Shodex sugar-KS-802 (8 x 300 mm) column. (B and C) MALDI-TOF-MS analysis of purified products. (D) ^13^C (DEPT135)-NMR spectra (500 MHz, D_2_O) of purified limit digest products generated from barely-β-glucan. Supplementary Figure 8 and panel A confirmed that the limit digested product of lichenan by the action of *Bp*GH16_MLG_ was G4G3G; therefore, purification and NMR were not performed for this trisaccharide. Limit digestion of both oligosaccharides of barley-β-glucan were separated by GPC and obtained molecule weight was 527.1302 (M+Na)^+^ and 689.2201 (M+Na)^+^ (B and C). ^13^C-DEPT-NMR characterized both oligosaccharides. Signature peaks at ~84 and ~78 ppm for these oligosaccharides were indicators of β-(1,3) and β-(1,4) linkages, respectively (D). An additional peak at 102.31 ppm and higher signal intensity of a peak at ~78 ppm in a G4G4G3G as compared to G4G3G suggests that second digested product of barley-β-glucan has an additional carbon with β-(1,4) linkage. Based on these analyses, barley-β-glucan digested products were identified as G4G3G and G4G4G3G. 200 mg barley-β-glucan was hydrolysed by *Bp*GH16_MLG_ and 20, 60 and 55 mg products of G4G3G, G4G3G + G4G4G3G and G4G4G3G were obtained after purification, respectively. Panel A was also used in Fig. 3A. It was re-used to provide an immediate reference for panel S8B.

**
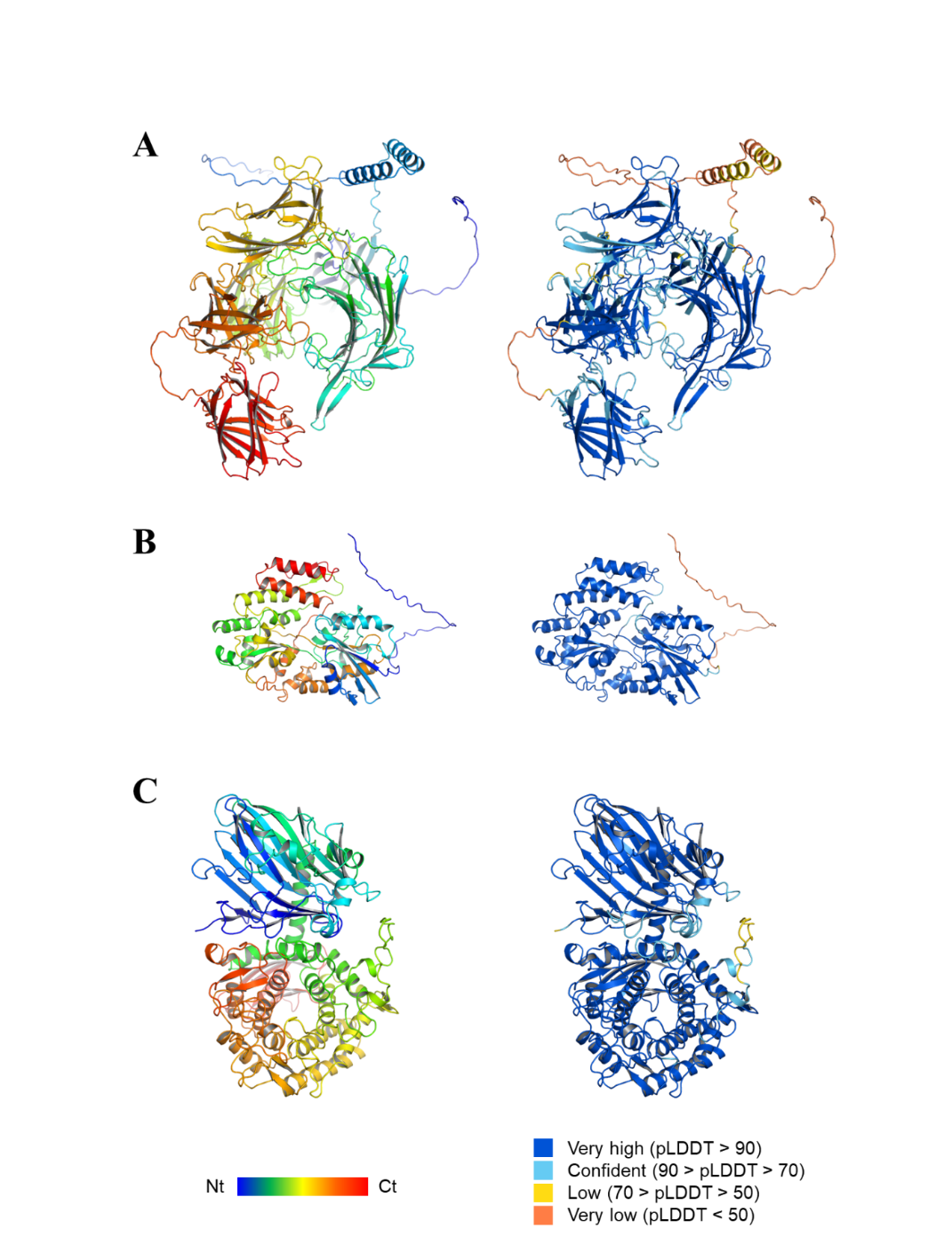
**

**Supplementary Figure 9. Confidence of AlphaFold2 models used in this study.** The ribbon models of (A) *Bp*GH16_MLG_, (B) *Bp*SBP_MLG_, and (C) *Bp*GH94-2 are colored in *rainbow* from N-terminus (Nt, *blue*) to C terminus (Ct, *red*) in left panels and are colored based on pLDDT score in right panels.


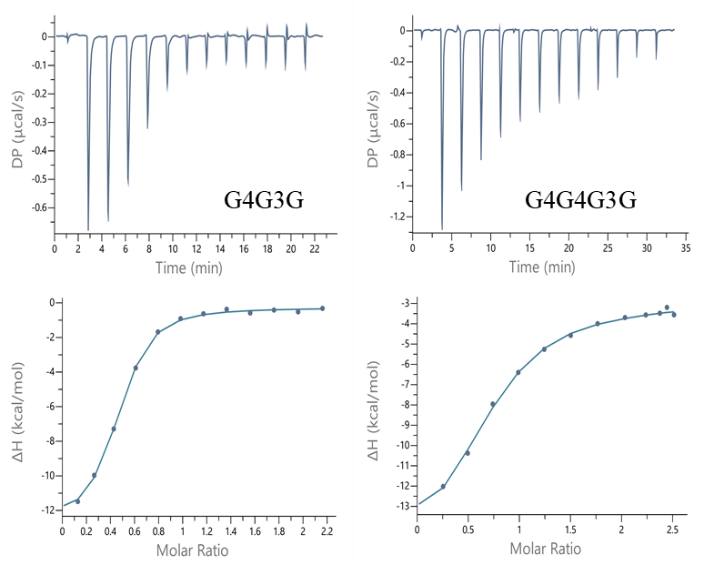


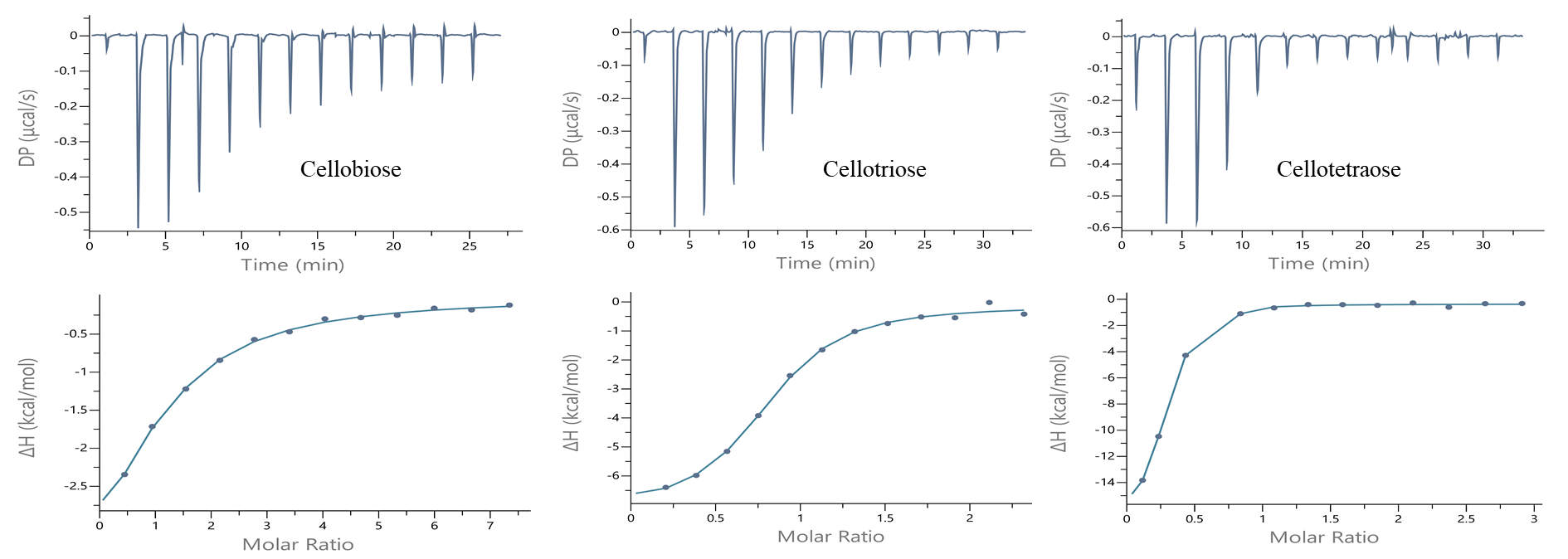


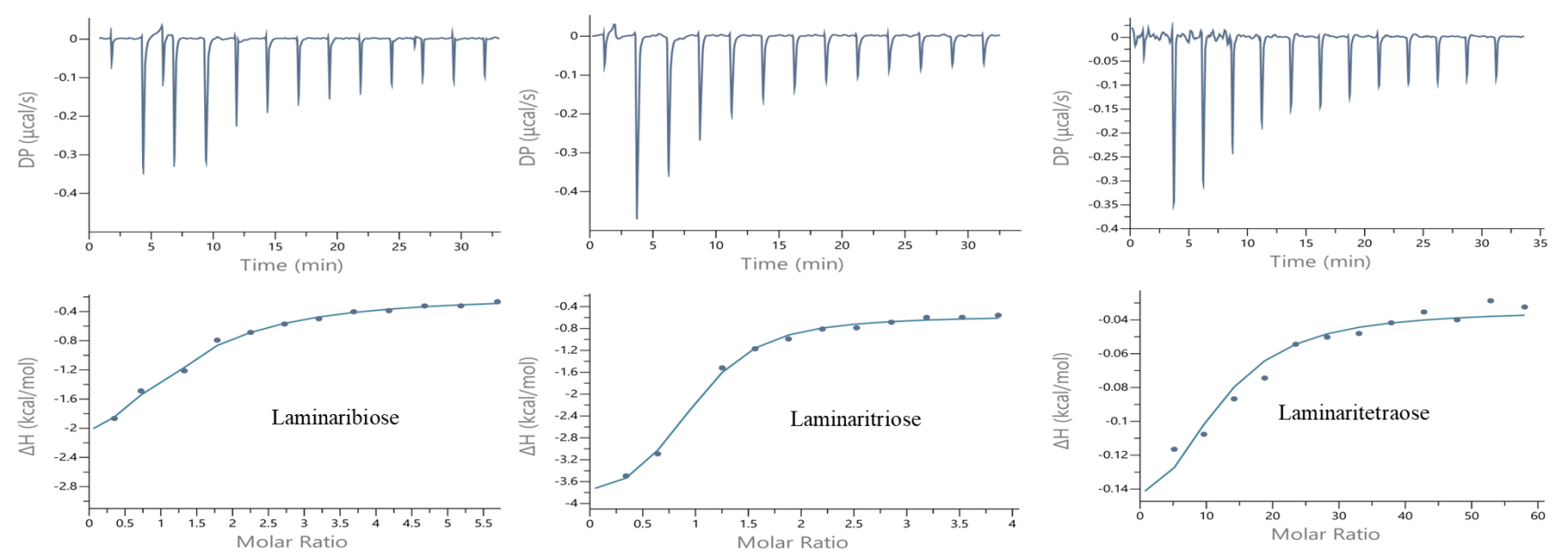


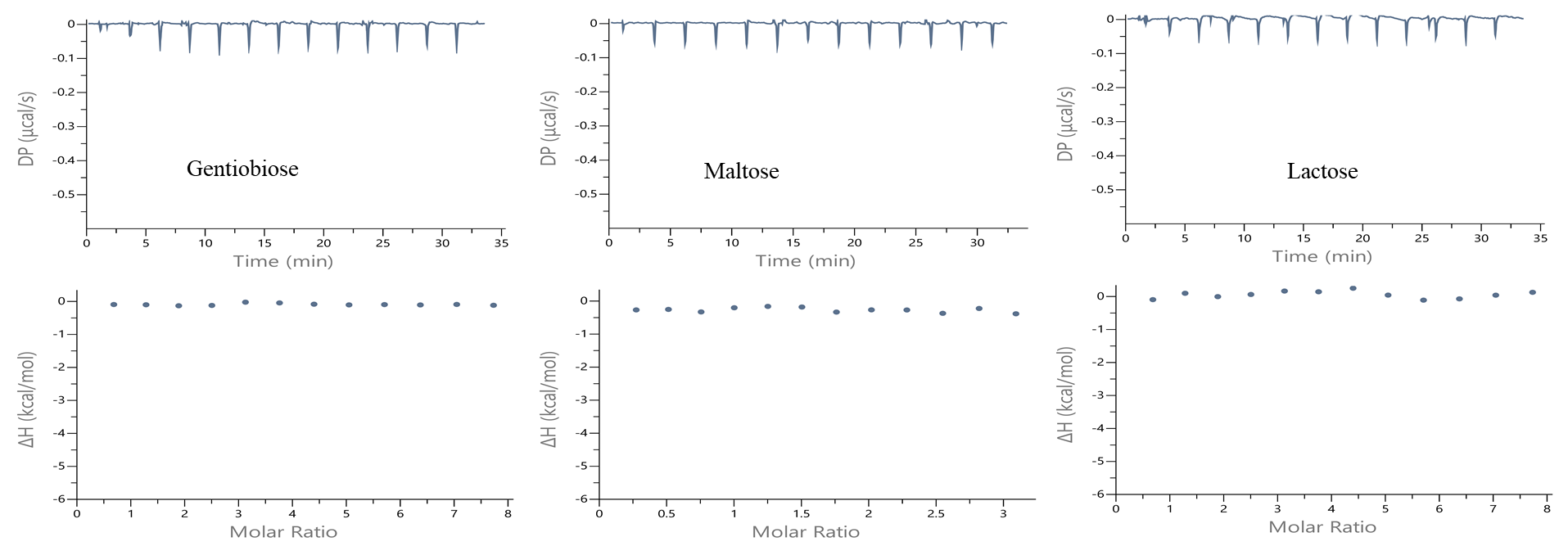


**Supplementary Figure 10.** **Isothermal calorimetry analysis (ITC)**. ITC determines affinities of *Bp*SBP_MLG_ with different oligosaccharides in sodium phosphate buffer (pH 7, 10 mM).


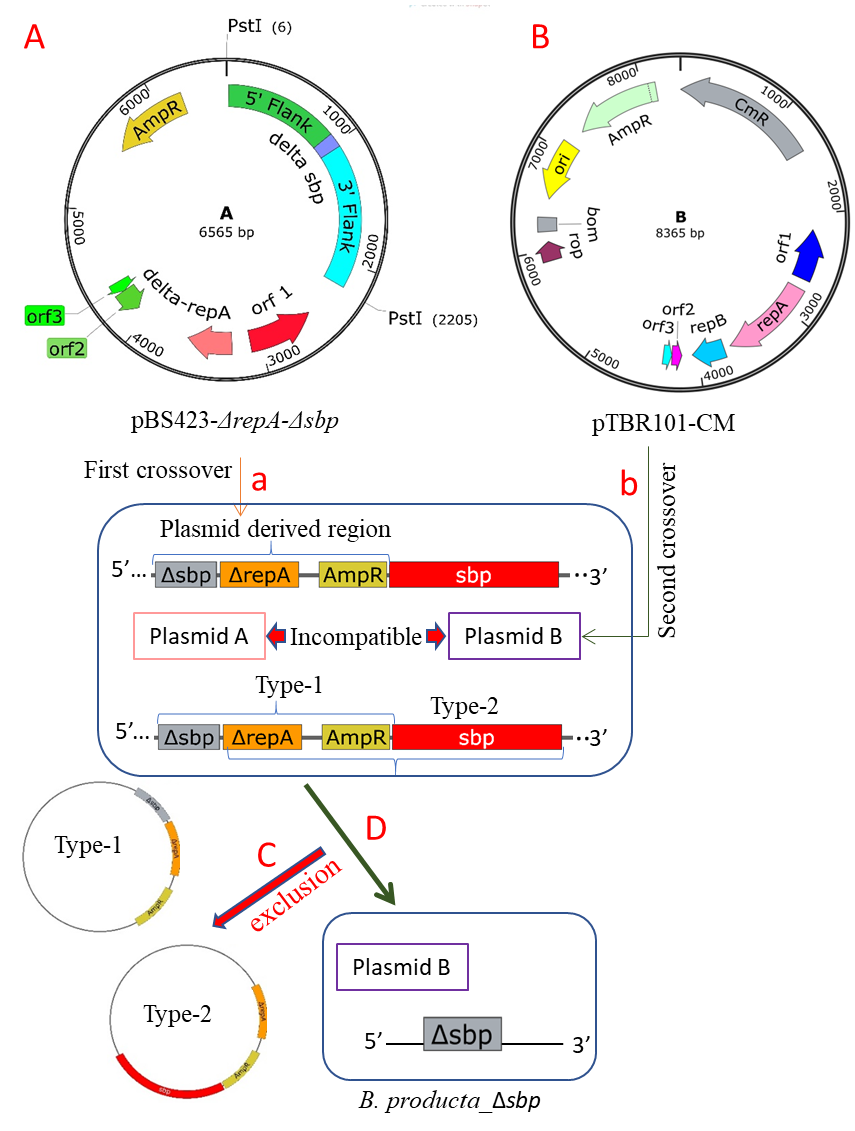


**Supplementary Figure 11. Designing of plasmids for making *sbp* mutant in the *B. producta*.** (A) The pBS423-Δ*repA-*Δ*sbp* was first transformed into the bacterium that was integrated into the genome at the homologous region. During this first crossover (a), the genome harbors both the gene alleles (solute Δ*sbp* and sbp) as mentioned with plasmid-derived regions. (B) RepA^+^ plasmid (pTBR101-CM) was supplied during the second crossover (b) that results in a supply of the plasmid replication protein (RepA) to the bacterial cell. The pBS423-Δ*repA* is expected to excise from the genome in the presence of RepA with *sbp* region due to incompatibility between two plasmids (C and D). Thus, *B. producta* Δ*sbp* is mutated during the second crossover event.


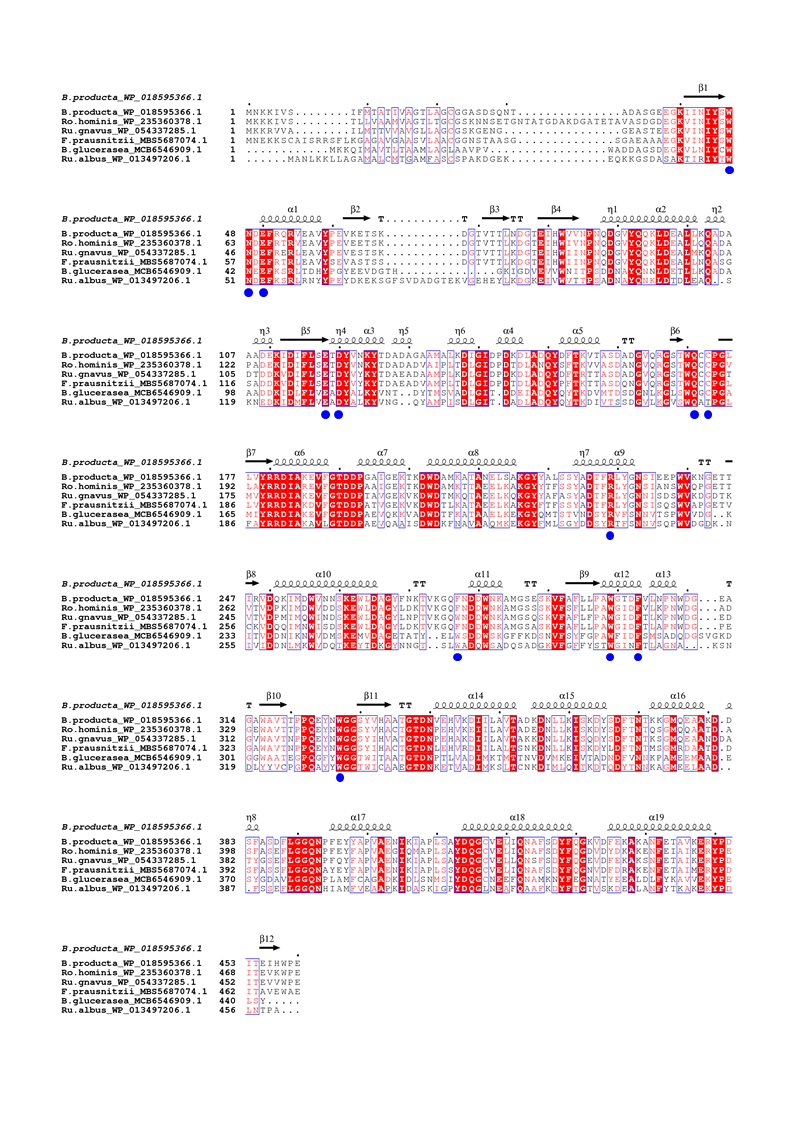
**Supplementary Figure 12. Multiple sequence alignment of *Bp*SBP_MLG_ and its orthologs.** The amino acid sequences of orthologs from *Roseburia hominis* (WP_235360378.1, sequence identity = 80.7%), *Ruminococcus gnavus* (WP_054337285.1, sequence identity = 78.4%), *Ruminococcus albus* (WP_013497206.1, sequence identity = 42.9%), *Blautia glucerasea* (MCB6546909.1, sequence identity = 44.7%), and *Faecalibacterium prausnitzii* (MBS5687074.1, sequence identity = 74.8%) are used. The alignment was carried out using ClustalOmega and the figure was generated using ESPript 3.0. Amino acid residues shown in the Fig. 4 are indicated with blue circles. The secondary structures of *Bp*SBP_MLG_ in the AlphaFold2 model are indicated above the amino acid sequence.


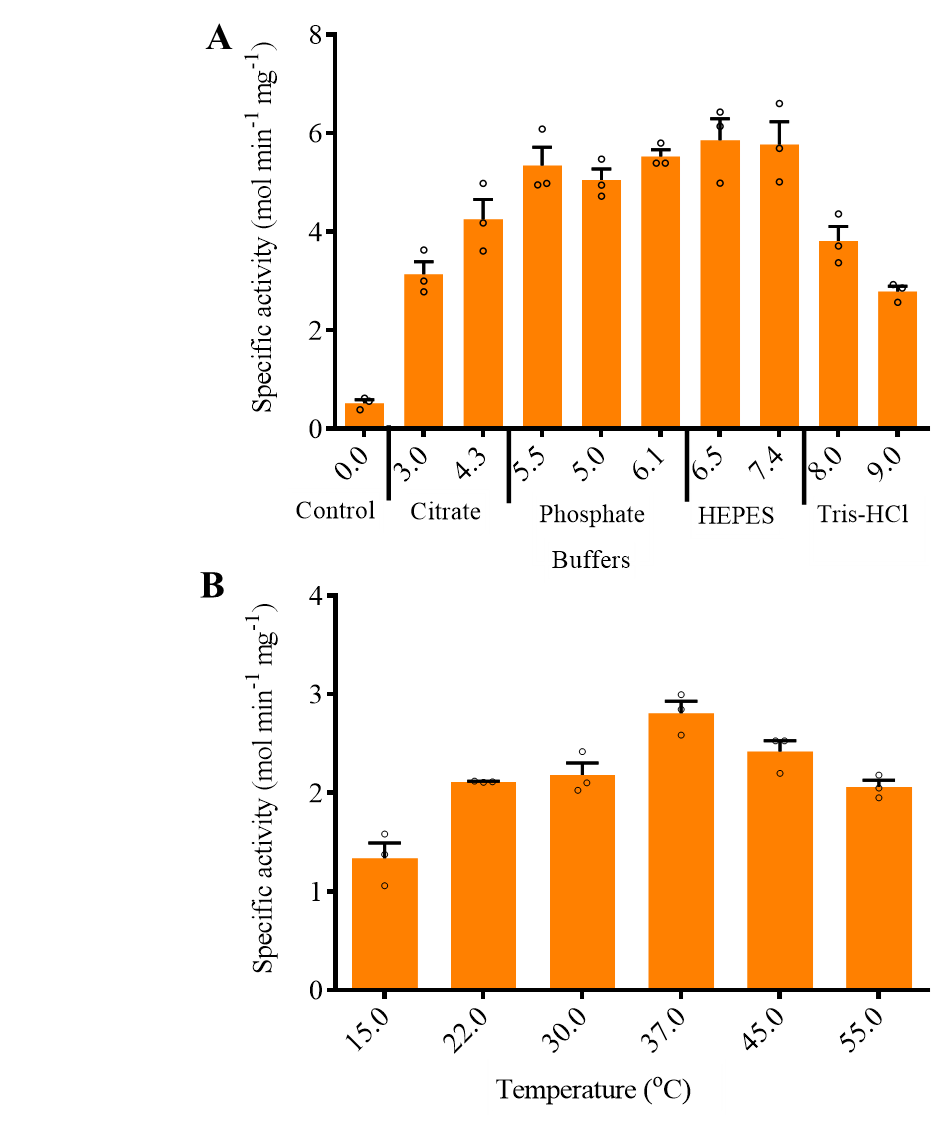


**Supplementary Figure 13.** Effect of the different pH (A) and temperatures (B) on the enzymatic activity of *B_P_*GH94_MLG_. A phosphate release assay was used to measure released inorganic phosphate.


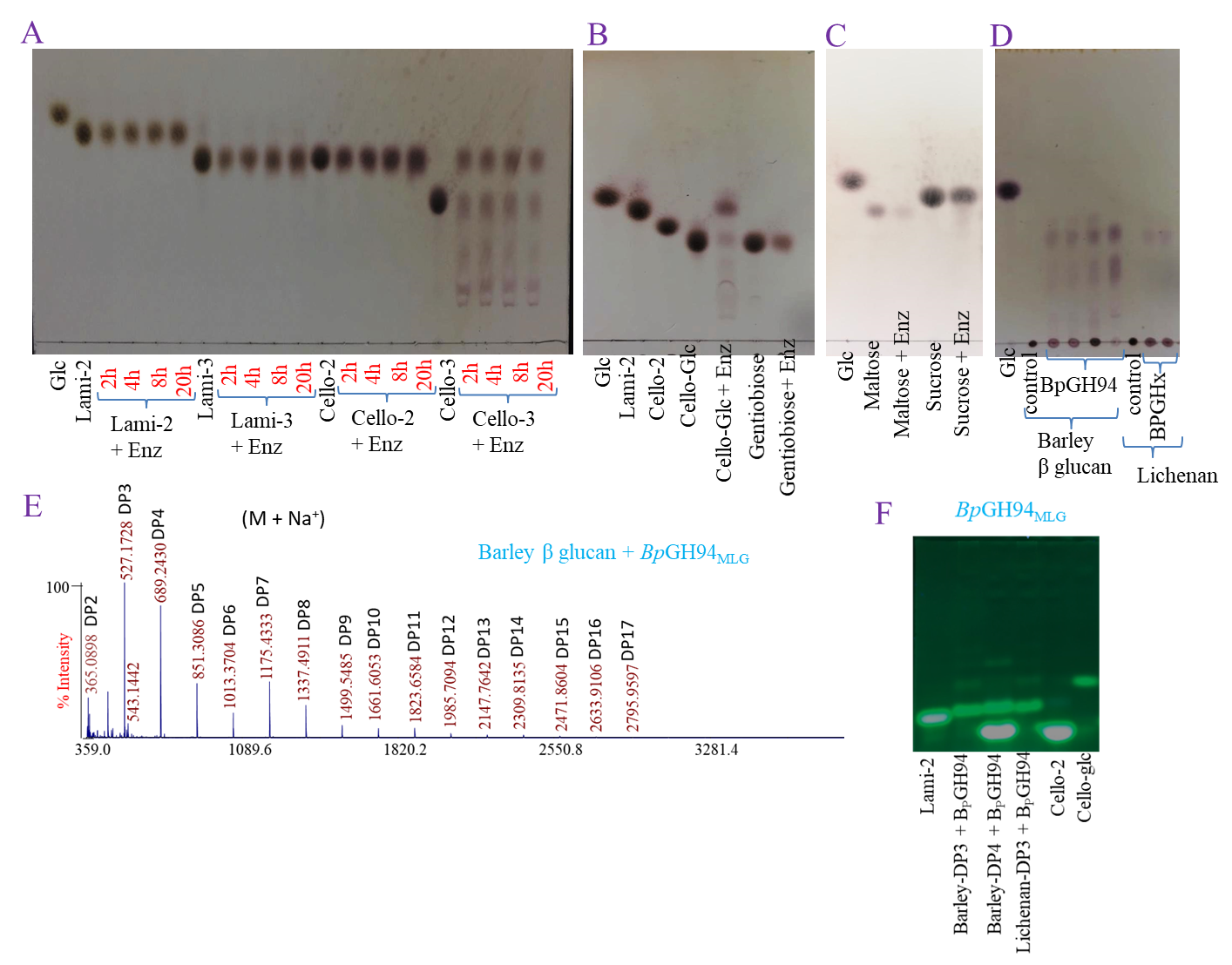


**Supplementary Figure 14. Activity and product limit digest of *Bp*GH94_MLG_.** (A) Thin layer chromatography (TLC) analysis of the phosphorolysis reaction performed with the *Bp*GH94_MLG_ in the presence of 10 mM laminaribiose (Lami-2), laminaritriose (Lami-3), cellobiose (Cello-2) and cellotriose (Cello-3), 10 mM inorganic phosphate (IP) and 20 µl (100 µg/ml) of the enzyme using optimized conditions. MALDI-TOF-MS analyzed produced products. (B) TLC of cellobiose-β-(1,3)-glucose (Cello-Glc) and gentiobiose. (C) TLC of maltose and sucrose. (D) TLC of barley- β-glucan and lichenan. The MALDI-TOF-MS profiles of barley-β-glucan, which were phosphorolysis by *Bp*GH94_MLG,_ highlighting that it can cleave longer chain glucans. (F) The *Bp*GH16_MLG_ hydrolyzed purified products of barley-β-glucan and lichenan were fluorescently tagged and run onto a 37% polyacrylamide gel. Oligosaccharides generated from barley-β-glucan and lichenan by *Bp*GH16_MLG_ were purified using Toyopearl HW-40 resin and HPLC using Shodex sugar-KS-802 (8 x 300 mm) column. 80.6 mg purified G4G3G was obtained from 100 mg lichenan after hydrolyzing with *Bp*GH16_MLG._ 200 mg barley-β-glucan was hydrolysed by *Bp*GH16_MLG_ and 20, 60 and 55 mg products of G4G3G, G4G3G + G4G4G3G and G4G4G3G were obtained after purification, respectively. Glc-1-P was not observed on the FACE image due to it was probably run out of gel along with fluorescent dye.

**Supplementary Figure 15. Michaelis-Menten kinetics of the *B_P_*GH94_MLG_.** Michaelis-Menten plot demonstrates the enzymatic phosphorolysis of different oligosaccharides using *p*-hydroxybenzoic acid hydrazide (PAHBAH) assay. It was performed with three biological replicates.


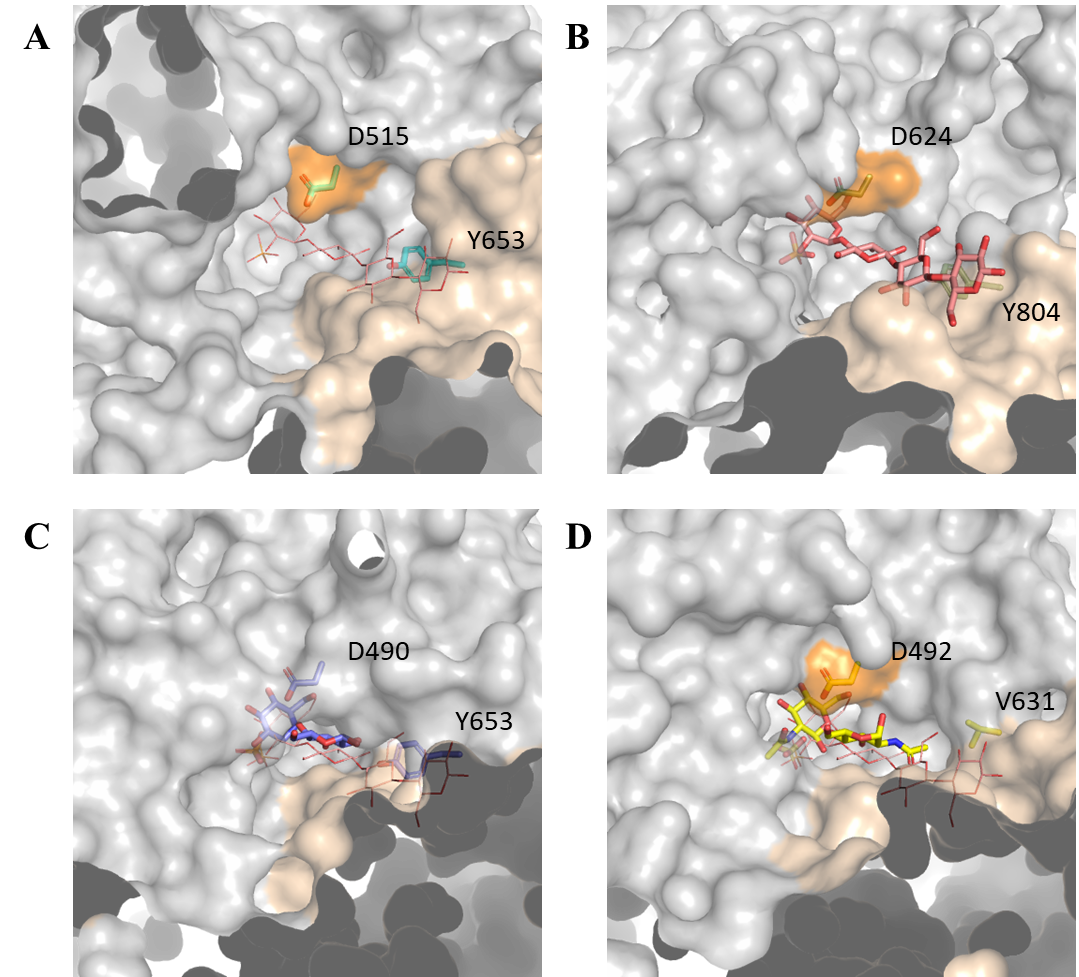


**Supplementary Figure 16. Comparison of the active sites between *Bp*GH94_MLG_ and GH94 phosphorylases.** (A) Molecular surfaces of *Bp*GH94_MLG_, (B) *Rt*CDP complexed with cellotetraose and phosphate (PDB 5NZ8), (C) *Cg*CBP (PDB 3QG0) complexed with 1-deoxynojirimycin, glucose and phosphate, and (D) *Vp*ChBP complexed with GlcNAc and sulfate (PDB 1V7X) are shown in gray and each dimeric counterpart in wheat. Side chains of catalytic acid residues (Asp) and residues corresponding to Y653 of *Bp*GH94_MLG_ and ligands are shown as a stick model. Thin stick model of cellotetraose (pink) in *Rt*CDP is superimposed to other GH94 structures. Solvent-accessible areas of catalytic acid residues are shown in orange.

**
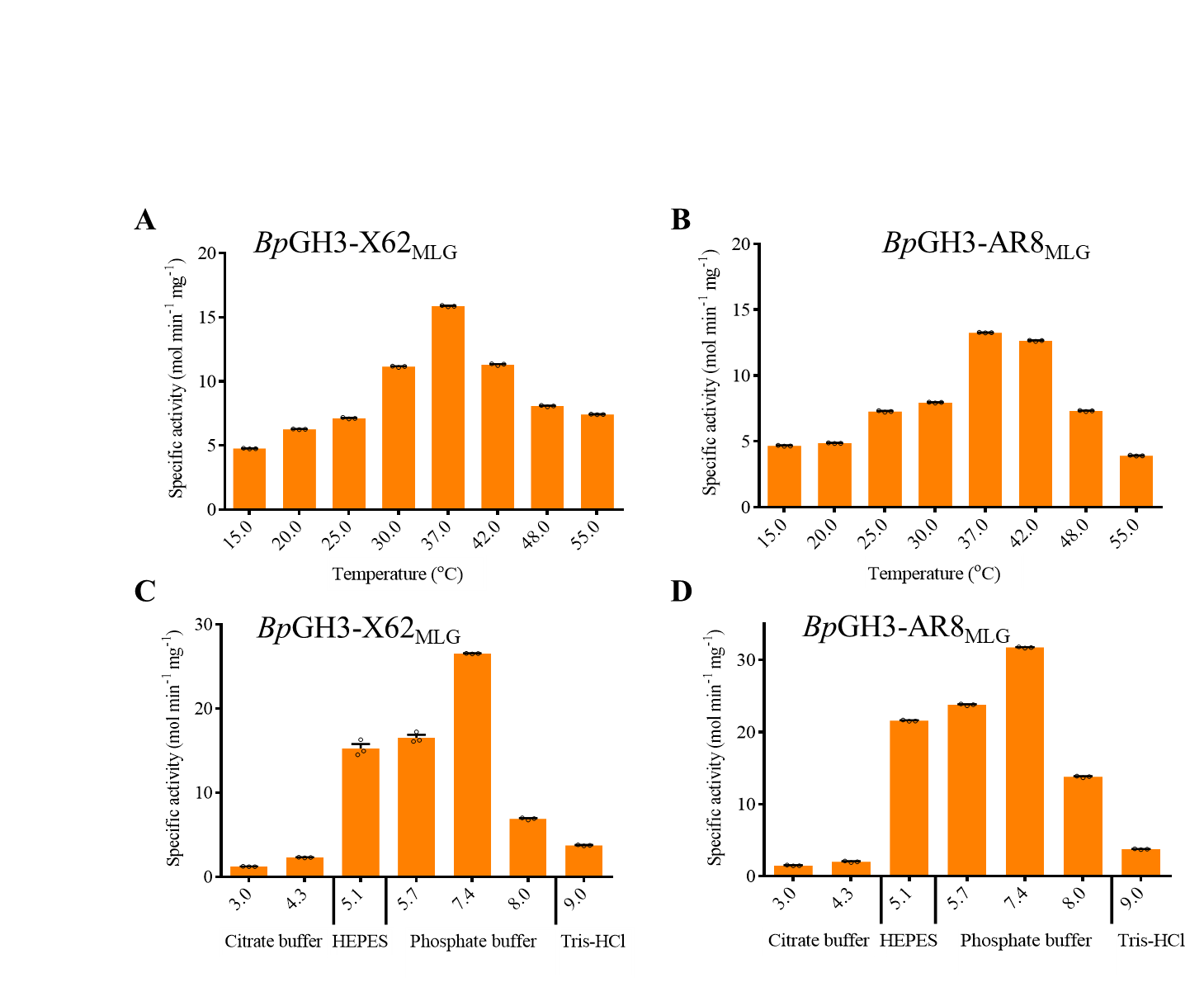
**

**Supplementary Figure 17.** Effect of different temperatures (A and B) and buffering pH (C and D) on the enzymatic activity of *Bp*GH3 using *p*NP-β-Glc (final concentration was 2 mM). specific activity was calculated on the released amount of para-nitrophenol.


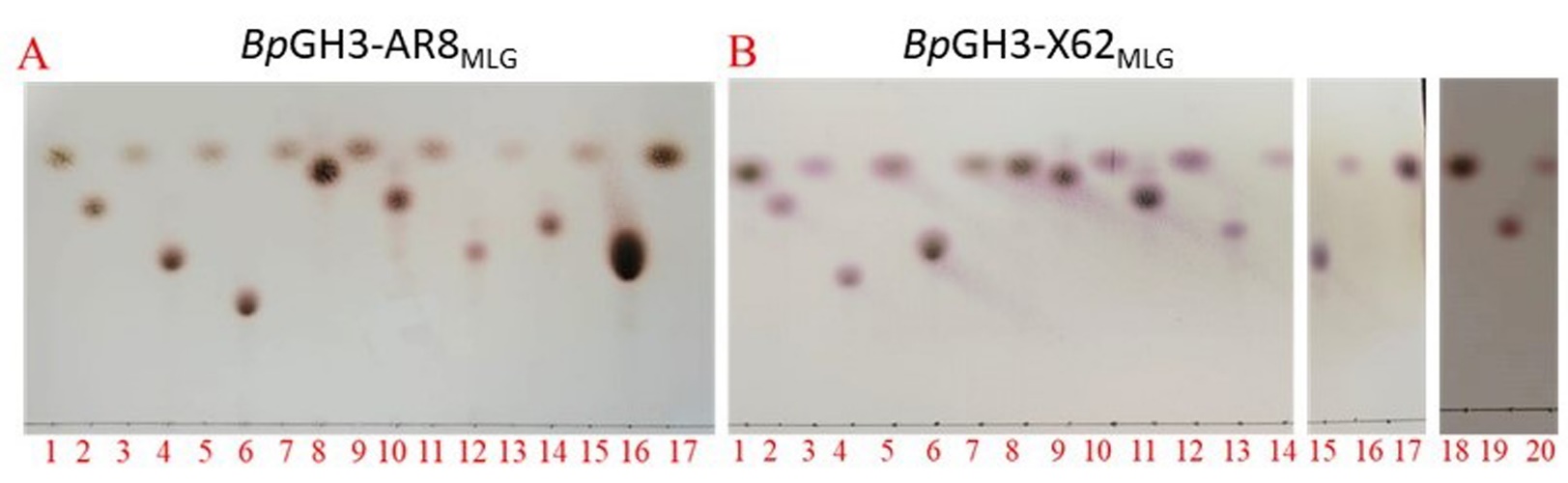


**Supplementary Figure 18.** **Cytoplasmic digestion of oligosaccharides generated from barley β*-*glucan and lichenan.** Cytoplasmic (A) *Bp*GH3-AR8_MLG_ and (B) *Bp*GH3-X62_MLG_ hydrolyzed β-(1,3), β-(1,4), and β-(1,6) linked oligosaccharides. **Numbers represent (A**): (1) Glucose, (2) Cellobiose, (3) Cellobiose + *Bp*GH3-AR8_MLG_, (4) Cellotriose, (5) Cellotriose + *Bp*GH3-AR8_MLG_, (6) Cellotetraose, (7) Cellotetraose + *Bp*GH3-AR8_MLG_, (8) Laminaribiose, (9) Laminaribiose + *Bp*GH3-AR8_MLG_, (10) Laminaritriose, (11) Laminaritriose + *Bp*GH3-AR8_MLG_, (12) G4G4G3G, (13) G4G4G3G + *Bp*GH3-AR8_MLG_, (14) G4G3G, (15) G4G3G + *Bp*GH3-AR8_MLG_, (16) Gentiobiose, (17) Gentiobiose + *Bp*GH3-AR8_MLG._ **In (B)-** (1) Glucose, (2) Cellobiose, (3) Cellobiose + *Bp*GH3-X62_MLG,_ (4) Cellotetraose, (5) Cellotetraose + *Bp*GH3-X62_MLG,_ (6) Cellotriose (7) Cellotriose + *Bp*GH3-X62_MLG,_ (8) Glucose, (9) Laminaribiose, (10) Laminaribiose + *Bp*GH3-X62_MLG,_ (11) Laminaritriose, (12) Laminaritriose + *Bp*GH3-X62_MLG,_ (13) G4G4G3G, (14) G4G4G3G + *Bp*GH3-X62_MLG,_ (15) G4G3G, (16) G4G3G + *Bp*GH3-X62_MLG,_ (17) Glucose, (18) Glucose, (19) Gentiobiose and (20) Gentiobiose + *Bp*GH3-X62_MLG._


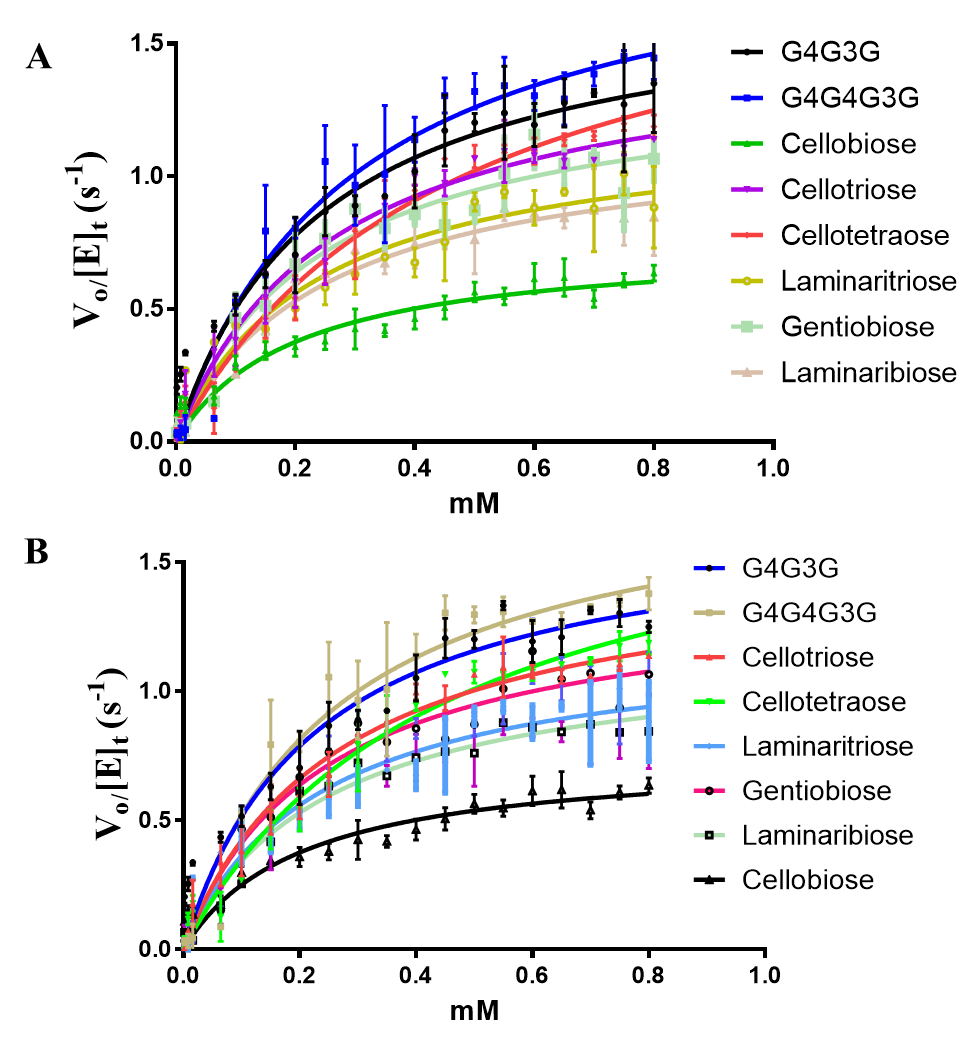


**Supplementary Figure 19. Michaelis-Menten kinetics of the *Bp*GH3-AR8_MLG_ (A) and *Bp*GH3-X62_MLG_ (B).** Michaelis-Menten plot demonstrates the enzymatic hydrolysis of different oligosaccharides using *p*-hydroxybenzoic acid hydrazide (PAHBAH) assay. Kinetics parameters were performed with three biological replicates.


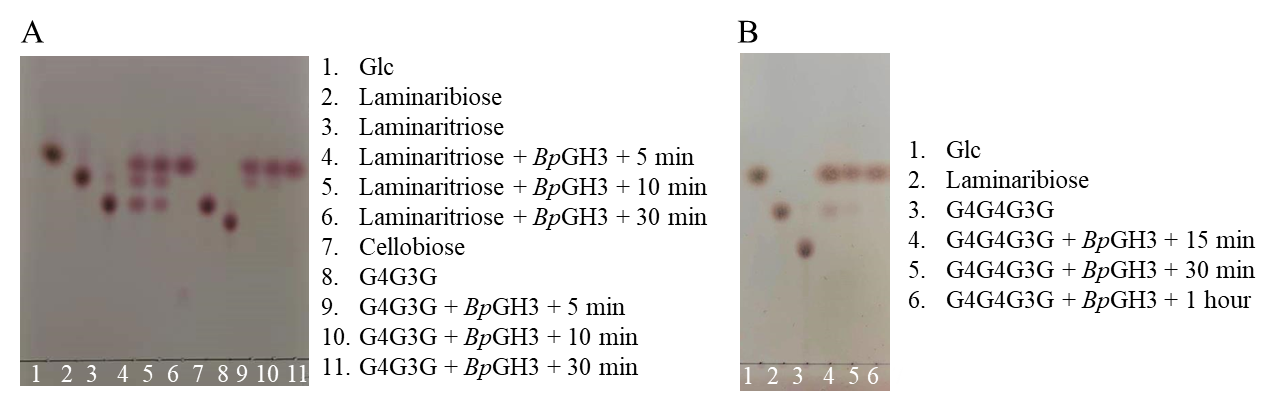


**Supplementary Figure 20:** Time dependent degradation of various oligosaccharides by the *Bp*GH3-AR8_MLG._ (A) Time dependent degradation of laminaritriose and G4G3G. (B) Time dependent degradation of G4G4G3G.

**
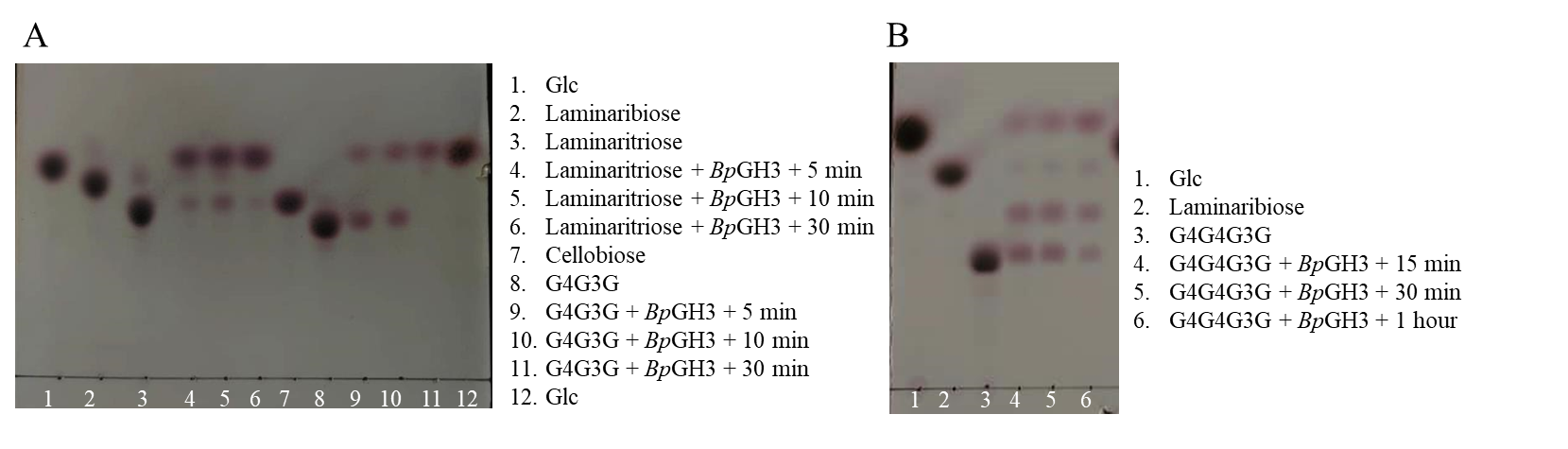
**

**Supplementary Figure 21.** Time-dependent degradation of various oligosaccharides by *Bp*GH3-X62_MLG._ (A) Time-dependent degradation of laminaritriose and G4G3G. (B) Time-dependent degradation of G4G4G3G.


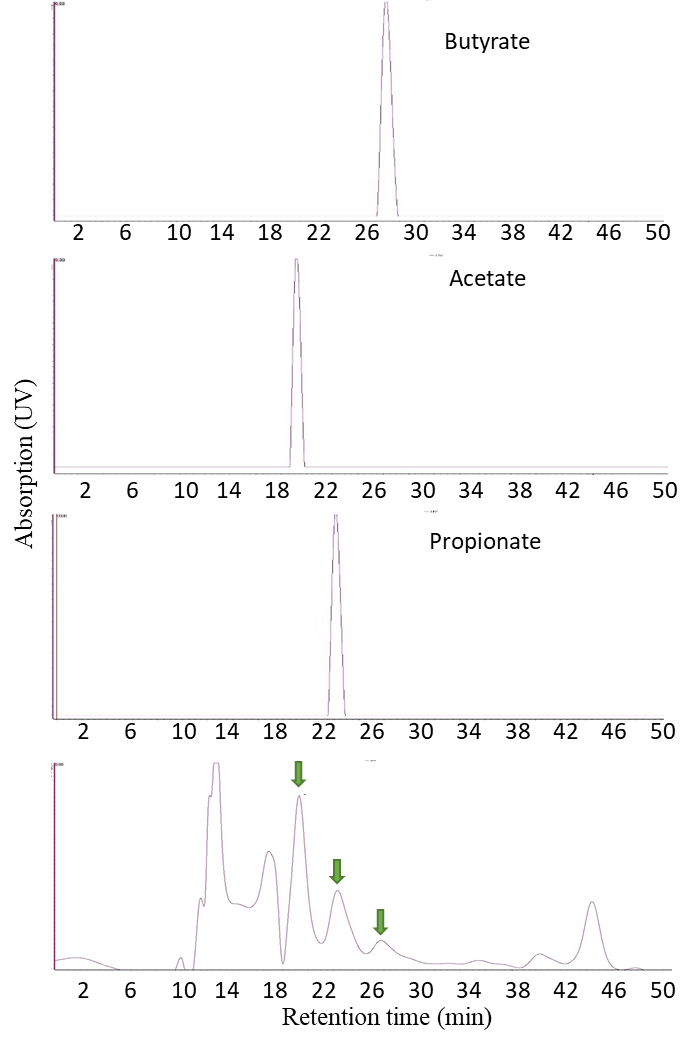


**Supplementary Figure 22.** Qualitative analysis of short chain fatty acids (SCFAs) produced by *B. producta* ATCC 27340 when it was grown on a minimal medium containing 1% barley-β-glucan. Peaks were confirmed by retention time of standards. Acetate, propionate and butyrate were present in supernatant in the ratio of 6.5:2.5:1.


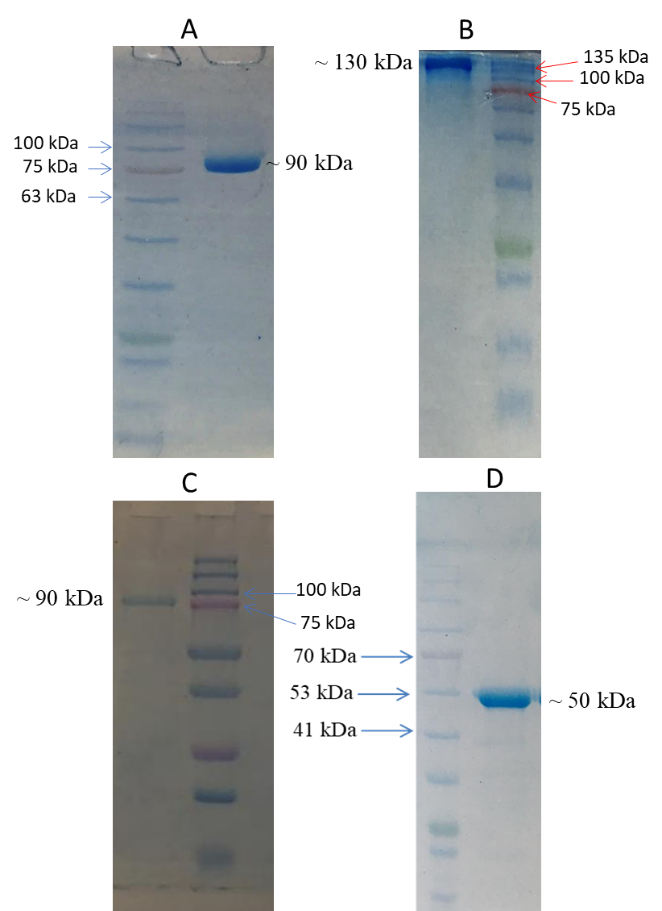


C D


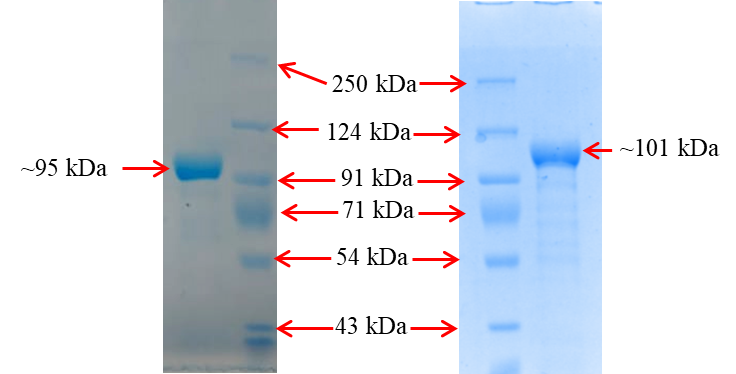


**Supplementary Figure 23.** 10% sodium dodecyl-sulfate polyacrylamide gel electrophoresis (SDS-PAGE) - (A) *Bp*GH94_MLG_, (B) *Bp*GH16_MLG_, (C) *Bp*GH3-X62_MLG_, and (D) *Bp*GH3-AR8_MLG_.


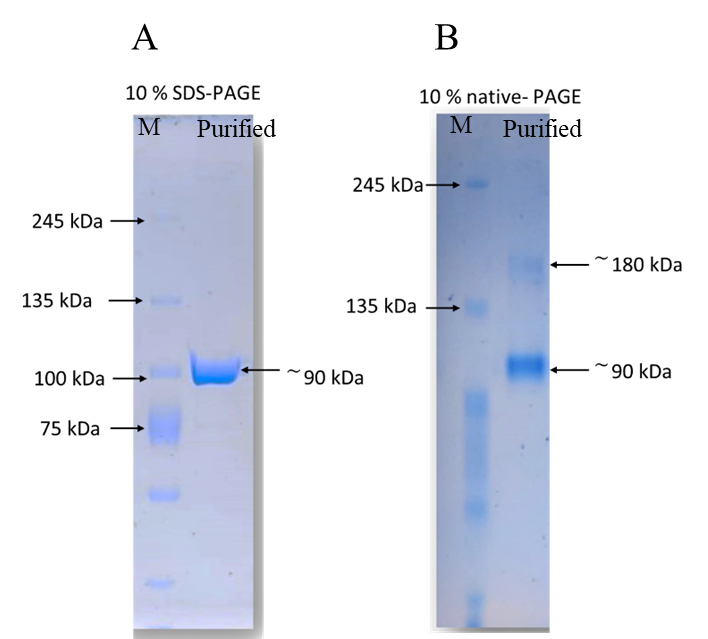


**Supplementary Figure 24.** 10% SDS-PAGE (A) and 10% native-PAGE for BpGH94_MLG_ (B). Native-PAGE confirmed that BpGH94_MLG_ exists in dimer form as it was observed in crystal structure Fig. 5A. Dimerization of other enzymes belong to GH94 families was also reported (55, 90-93). Panel B of Fig. S3 is reused in this Fig. as panel A for providing an immediate reference to dimerization nature of a recombinant *Bp*GH94_MLG_.

**References**

1. Zhang, H., and Row, K. H. (2015) Extraction and separation of polysaccharides from *Laminaria japonica* by size-exclusion chromatography. J. Chromatogr. Sci. **53**, 498-502
2. Aman, P., and Graham, H. (1987) Mixed-linked beta-(1,3), (1,4)-D-glucans in the cell walls of barley and oats--chemistry and nutrition. Scand. J. Gastroenterol. Suppl. **129**, 42-51
3. Viborg, A. H., Terrapon, N., Lombard, V., Michel, G., Czjzek, M., Henrissat, B. *et al.* (2019) A subfamily roadmap of the evolutionarily diverse glycoside hydrolase family 16 (GH16). *J. Biol. Chem.* **294**, 15973-15986
4. Jumper, J., Evans, R., Pritzel, A., Green, T., Figurnov, M., Ronneberger, O. *et al.* (2021) Highly accurate protein structure prediction with AlphaFold. *Nature* **596**, 583-589
5. Mirdita, M., Schütze, K., Moriwaki, Y., Heo, L., Ovchinnikov, S., and Steinegger, M. (2022) ColabFold: making protein folding accessible to all. *Nat. Methods* **19**, 679-682
6. Hirayama, Y., Sakanaka, M., Fukuma, H., Murayama, H., Kano, Y., Fukiya, S. *et al.* (2012) Development of a double-crossover markerless gene deletion system in *Bifidobacterium longum*: functional analysis of the alpha-galactosidase gene for raffinose assimilation. *Appl. Environ. Microbiol.* **78**, 4984-4994
7. Sawano, T., Saburi, W., Hamura, K., Matsui, H., and Mori, H. (2013) Characterization of *Ruminococcus albus* cellodextrin phosphorylase and identification of a key phenylalanine residue for acceptor specificity and affinity to the phosphate group. *FEBS Lett.* **280,** 4463-4473
8. Singh, R. P., Bhaiyya, R., Thakur, R., Niharika, J., Singh, C., Latousakis, D. et al. (2022) Biochemical basis of xylooligosaccharide utilisation by gut bacteria. *Int. J. Mol. Sci*. **23(6)**, 2992
9. McKee, L. S. (2017) Measuring enzyme kinetics of glycoside hydrolases using the 3,5-dinitrosalicylic acid assay. *Methods Mol. Biol.* **1588**, 27-36
10. Kumar, S., Stecher, G., Li, M., Knyaz, C., and Tamura, K. (2018) MEGA X: Molecular evolutionary genetics analysis across computing platforms. *Mol. Biol. Evol.* **35**, 1547-1549
11. Olafsdottir, E. S., and Ingolfsdottir, K. (2001) Polysaccharides from lichens: structural characteristics and biological activity. *Planta Med.* **67**, 199-208
12. Morales, D., Rutckeviski, R., Villalva, M., Abreu, H., Soler-Rivas, C., Santoyo, S. *et al.* (2020) Isolation and comparison of alpha- and beta-D-glucans from shiitake mushrooms (*Lentinula edodes*) with different biological activities. *Carbohydr. Polym.* **229**, 115521
13. Hamura, K., Saburi, W., Abe, S., Morimoto, N., Taguchi, H., Mori, H. *et al.* (2012) Enzymatic characteristics of cellobiose phosphorylase from *Ruminococcus albus* NE1 and kinetic mechanism of unusual substrate inhibition in reverse phosphorolysis. *Biosci. Biotechnol. Biochem*. **76**, 812-818
14. Honda, Y., Kitaoka, M., and Hayashi, K. (2004) Reaction mechanism of chitobiose phosphorylase from *Vibrio proteolyticus*: identification of family 36 glycosyltransferase in *Vibrio*. *Biochem. J*. **377**, 225-232
15. Kitaoka, M., Sasaki, T., and Taniguchi, H. (1992) Phosphorolytic reaction of *cellvibrio gilvus* cellobiose phosphorylase. *Biosci. Biotechnol. Biochem*. **56**, 652-655
16. Nidetzky, B., Eis, C., and Albert, M. (2000) Role of non-covalent enzyme-substrate interactions in the reaction catalysed by cellobiose phosphorylase from *Cellulomonas uda.* *Biochem. J*. **351 Pt 3**, 649-659
